# Correlation between ketones and mental fatigue in high fat‐induced obese and non‐obese rats

**DOI:** 10.14814/phy2.14930

**Published:** 2021-07-01

**Authors:** Paige Niepoetter, Carrie Butts‐Wilmsmeyer, Sepideh Kaviani, Coral Viernow, Hannah Ruholl, Chaya Gopalan

**Affiliations:** ^1^ Department of Nurse Anesthesia Southern Illinois University Edwardsville Edwardsville IL USA; ^2^ Department of Biological Sciences Southern Illinois University Edwardsville Edwardsville IL USA; ^3^ Center for Predictive Analytics Southern Illinois University Edwardsville Edwardsville IL USA; ^4^ Department of Applied Health Southern Illinois University Edwardsville Edwardsville IL USA

**Keywords:** cognition, fatigue, high‐fat diet, obesity, Sprague Dawley rats

## Abstract

Obesity, often caused by a diet high in calories and low physical activity, may induce physical fatigue, as experienced via decreased locomotor activity and mental fatigue such as impaired cognition. This study aims to evaluate glucose and ketone levels secondary to high‐fat diet (HFD) exposure and signs of physical and mental fatigue. Fifty‐four 7‐week‐old male Sprague Dawley rats (*Rattus norvegicus*) were assigned to either an HFD (*n* = 28) or a standard diet (SD; *n* = 26) for a 6‐week period during which body weight, blood glucose, and ketones were measured twice per week. An open field (OF) paradigm was used to measure locomotor activity, while novel object recognition (NOR) test was used as an indicator of cognition. Animals in the HFD group weighed more than SD rats (8.4 g; *p *< 0.05) starting at Day 11, blood glucose levels were higher in the HFD group versus SD rats (3.9 mg/dl; *p *< 0.05) beginning in Week 5, and ketones were lower for the HFD versus the SD group throughout the study (0.34 mmol/L on average; *p *< 0.05). Although there was no significant difference in locomotor activity between the HFD and SD groups (*p* = 0.12), regardless of diet, higher ketone levels were associated with increased NOR time and ratio between the familiar and novel objects (*p *< 0.01). Thus, this study provides evidence that an increased level of ketones is associated with greater cognitive performance and a lesser probability of experiencing mental fatigue.

## Graphical abstract

This study measured glucose and ketone levels secondary to high‐fat diet (HFD) exposure and physical and mental fatigue in rats. Higher ketone levels were associated with increased novel object recognition time and the ratio between the familiar and novel objects (*p* < 0.01) thus providing evidence that an increased level of ketones is associated with decreased mental fatigue.

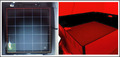

## 1 INTRODUCTION

In the United States, more than 40% of adults are classified as obese, which has been linked to the development of cardiovascular disease (CVD), type 2 diabetes, and various cancers, while also negatively affecting mental health (Apovian, 2016; de Wit et al., 2010; Hales et al., 2017). More specifically, obesity has been associated with impaired memory, compromised learning, executive functions, and brain atrophy (Solas et al., 2017). Chronic conditions such as Alzheimer's disease and dementia have also been associated with obesity (Solas et al., 2017). A meta‐analysis of 17 studies with 204,507 subjects found that individuals with obesity were 1.26 times more likely to experience depression than non‐obese individuals (de Wit et al., 2010). Yet another study with 1,423 participants found that male individuals with obesity, in particular, experienced adverse effects on cognitive performance (Elias et al., 2005). Many causes contribute to obesity, including lifestyle choices such as reduced physical activity and a diet high in saturated fats (Apovian, 2016; Pistell et al., 2010). Since behavioral and environmental factors are seen as significant contributors to the development of obesity, a diet‐induced obesity (DIO) model using a high‐fat diet (HFD) was utilized in this study to mimic the condition of obesity.

One of the lesser discussed consequences of HFD consumption and obesity is fatigue (Lim et al., 2005). Mental fatigue, commonly defined as “tiredness” and “feeling an absence of energy,” is a psychobiological state that is associated with impaired cognition (Boksem & Tops, 2008). Compromised mental performance that might occur secondary to obesity could be detrimental to the individual's well‐being and longevity (Elias et al., 2005). Studies using a DIO model in rodents have linked obesity via HFD to mental fatigue (Underwood & Thompson, 2016; Wang et al., 2017). Underwood and Thompson fed 3‐week‐old male Long Evans rats an HFD (58% kcal from fat) for 12 weeks, which resulted in obesity and increased glucose levels. At the end of the study, rats were subjected to spatial object recognition testing (SOR), which involved exposing rats to 2 objects for 5 min, holding the rats for a 30‐min intertrial interval, and then returning the rats to the apparatus to explore the object that is either in the same space as before or a novel space. Time spent with the objects in either the familiar space or novel space was compared as a measure of spatial memory. Rats fed an HFD performed worse during SOR testing than those fed SD (Underwood & Thompson, 2016). In a separate study, an HFD (60% kcal fat) fed for 3 weeks produced worse outcomes in novel object recognition (NOR) and Y maze testing in the 5‐week‐old mice compared to mice fed an SD (Wang et al., 2017). These trends suggest that DIO could cause adverse cognitive outcomes. It is speculated that insulin resistance and activation of proinflammatory pathways that follow the induction of obesity may be responsible for these effects (Wang et al., 2017).

There are studies aiming to understand how to reverse the adverse cognitive effects caused by obesity. Ketone levels appear to have an impact on cognition (Cunnane et al., 2016; Maalouf et al., 2009). In a study by Hernandez et al. (2018), rats fed a ketogenic diet (KD) containing 75% kcal fat for 12 weeks experienced elevated ketone levels. After exposure to this treatment, rats underwent a dual working memory/bi‐conditional association task as a measure of cognition. It was found that rats with elevated ketone levels (due to receiving a KD) had better cognitive performance than rats receiving SD (Hernandez et al., 2018). There are multiple proposed mechanisms of action of ketones for the reported mental benefits. One of them appears to be limited neuronal apoptosis and yet another mechanism is the ability of ketone in increasing energy production by promoting mitochondrial reproduction in neurons (Cunnane et al., 2016; Maalouf et al., 2009).

Physical fatigue is another effect of obesity and is commonly associated with decreased locomotor activity (Resnick et al., 2006; Xu et al., 2018). Physical fatigue studies in humans often use self‐assessments, such as the multidimensional fatigue symptom inventory (MFSI), a self‐reported assessment of general, emotional, physical, and mental fatigue (Lim et al., 2005). Since the evaluation of fatigue often incorporates a subjective component, examining this effect in rodents can be difficult, and studies are still underway to confirm the best approach (Zaretsky et al., 2018).

To precisely understand the negative impacts of obesity on mental and physical fatigue, it is essential first to establish any metabolic changes that precipitate and may mitigate these effects. This study aimed to use a DIO model to investigate if metabolic changes related to obesity such as blood glucose and ketone levels and body weight may cause signs of fatigue, including decreased locomotor activity and impaired cognition in rats fed an HFD. This information will help lay the foundation for future studies involving rodent models for DIO and fatigue at cellular and molecular levels.

## 2 MATERIALS AND METHODS

### 2.1 Animals

Seven‐week‐old male Sprague Dawley rats (*n* = 54) were acquired from Envigo Labs, Indianapolis, IN. Animals were housed individually under controlled laboratory conditions (12‐h light/dark cycle with lights on at 7:00 pm at a room temperature of 20.0–22.2℃) in solid‐bottom cages with aspen chip bedding. Body mass was manually collected weekly using a digital scale (Table 22). Blood glucose and ketone levels were obtained twice a week throughout the study via the tail prick method (Fluttert et al., 2000). All protocols described were approved by the Southern Illinois University Edwardsville Institutional Animal Care and Use Committee (040618‐CG2).

### 2.2 Diet

Upon arrival, animals were randomly placed into one of the two diet groups: 28 rats were put on a HFD and 26 rats were put on a standard diet (SD). The HFD consisted of 60% of energy from fat, 20% from carbohydrate, and 20% from protein (formula D12492 from Research Diets Inc.) and an energy density of 5.21 kcal/g of food. Fat sources included lard and soybean oil. The SD (diet identification 5663, Mazuri rat chow) contained 17% of energy from fat, 56% from carbohydrate, and 27% from protein. The SD had an energy density of 3.41 kcal/g of food. Animals had access to food and water *ad libitum*. Diet and chow characteristics are summarized in Table 1.

**TABLE 1:** Macronutrient composition

	HFD	SD
Fat (kcal)	60%	17%
Carbohydrate (kcal)	20%	56%
Protein (kcal)	20%	27%
Energy Density (kcal/g)	5.21	3.41
Fat source	Lard, soybean oil	Flaxseed oil, polyunsaturated fatty acids

Abbreviations: HFD, high‐fat diet; kcal, kilocalories; kcal/g, kilocalories per gram of food; SD, standard diet.

### 2.3 Blood testing of glucose and ketone levels

Capillary blood sampling was used to obtain overnight fasting glucose and β‐hydroxybutyrate (ketone) levels between 7:00 and 9:00 am on Days 3 and 6 each week. Blood samples for this testing were obtained by pricking the rats’ tail veins using 26‐gauge lancets (Fluttert et al., 2000). Results were obtained immediately (Table 2) using a *Keto*‐*Mojo* (Napa Valley, CA) glucose and ketone meter (model TD‐4279).

**TABLE 2:** Baseline averaged physical parameters

	HFD	SD
Body mass (g)	219 ± 16	215 ± 16
Glucose (mg/dl)	93 ± 8	101 ± 6
Ketone (mmol/L)	0.67 ± 0.4	0.98 ± 0.3

Abbreviations: g, grams; HFD, high‐fat diet; mg/dl, milligram per deciliter; mmol/L, millimole per liter; SD, standard diet.

#### 2.3.1 Behavioral testing

In rodent models, physical fatigue is inferred by measuring locomotor activity, which is often obtained using OF paradigm because the ANY‐maze software is able to capture the distance traveled by the animal as well as the speed with which the animal travels within the arena. Mental fatigue can be determined indirectly by measuring recognition memory during NOR testing (Antunes & Biala, 2012; Gould et al., 2009). OF testing is a commonly used behavioral test that measures exploratory behavior, anxiety, and locomotor activity. During this test, movements that are recorded consist of line crossings, distance/time moving, rearing, and freezing (Gould et al., 2009). For this study, the comparison of the distance traveled by the rats while in the arena between the SD and HFD groups was used as an indirect measure of physical fatigue. This method was included with the hopes of adding to the repertoire of behavioral tests utilized to assess fatigue in rats.

The open field apparatus consisted of a 100 cm × 100 cm opaque Plexiglass arena divided into central and peripheral zones. The animal explored the arena freely for 6 min, tracked using ANY‐maze video tracking system (Stoelting, Wood Dale, Illinois). OF was used to record distance traveled (meters) concerning the natural physical activity of the animal. OF was measured daily over 8 days in the dark under red light conditions during their night cycle.

Upon completing the 8‐day OF testing, the animals underwent a NOR study to measure mental fatigue for an additional 8 days. Motivation to learn and remember is based on animals’ natural tendency to explore unfamiliar objects (Antunes & Biala, 2012). A set of five objects of different shapes, colors, and dimensions were used. During the familiarization phase, one object was placed in the opaque Plexiglass arena described above, and the animal was allowed to investigate the object for 5 min freely. The animal was then returned to a holding cage for an inter‐exposure interval (IEI) to await being returned to the arena. The first IEI, the 0‐h test, occurred immediately after the 5 min familiarization phase. The following IEIs were as follows: 24‐h (1 day), 72‐h (3 days), and 168‐h (7 days). Once the animal was returned to the arena after each IEI, they were allowed to investigate either the previous and now familiar object or a novel object. The duration spent investigating the new object versus the familiar object defines the animal's recognition of the old object and provides a numerical value to memory.

#### 2.3.2 Statistical analyses

Using G*Power 3.1.9.4 and the following parameters (repeated‐measures ANOVA, 0.05, and weak to moderate correlation among repeated measurements = 0.20), it was determined that the sample size needed to obtain a power of at least 0.80 was 16 animals for a large effect of diet and 54 for a moderate effect of diet. It was hypothesized that diet would exhibit a large or moderately large effect ($\eta^{2\;}=0.3)$, but 54 total samples were included as a precaution. Prior to analysis, the expected effect size was uncertain. To best estimate the sample size needed to achieve an a priori power of 80%, we assumed a relatively large partial eta squared of 0.30. A 10% increase in body weight while being on HFD compared to standard diet‐fed controls was considered as an obese rat. Any animals that did not gain weight (21%) with HFD were excluded from statistical analyses.

#### 2.3.3 ANOVA of individual variables

All variables were analyzed using a repeated‐measures analysis of variance (ANOVA) in PROC MIXED of SAS (version 9.4), although slight variations in this repeated‐measures ANOVA were present due to small variation in the data collection. Average weekly ketone and glucose concentrations were analyzed using repeated‐measures ANOVA, except the daily time measurement was replaced with a weekly term.

The OF distance was collected daily for 8 days, thereby allowing a measures ANOVA with a first‐order autoregressive variance–covariance structure to be used, except the smaller range of days lent itself to a linear as opposed to the quadratic effect of time. The novel preference was calculated in two different ways, first as the total amount of time spent with the novel object, and second as the natural log of the ratio of time spent with the novel object to the time spent with the familiar object. This variable was collected at Days 31 (0), 32 (1), 34 (3), and 38 (7) in the experiment. Although this design also lends itself to repeated‐measures ANOVA, as otherwise previously described, the unequal spacing of the time points prohibited using an autoregressive model, as previously used, and therefore necessitated the use of a more complex variance–covariance structure.

#### 2.3.4 Correlation and discriminant analysis

The Pearson correlations between all pairwise comparisons of variables were calculated using PROC CORR in SAS. The average weekly glucose, ketone, and weight at Week 3 and the inflection time point for glucose were subjected to a linear discriminant analysis using PROC DISCRIM. Specifically, whether the covariance matrices could be pooled was tested using a chi‐squared test of homogeneity within covariance matrices (*α* = 0.05), and the following linear discriminant model was fit using a multivariate normal distribution. The misclassification error rate was calculated based on the cross‐validation error rate.

## 3 RESULTS

### 3.1 Physiological characteristics

**FIGURE 1:**
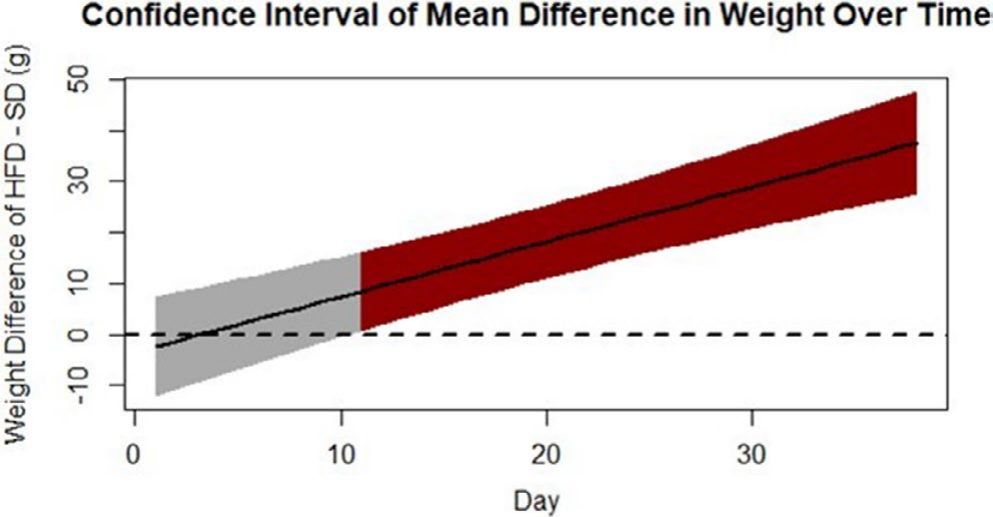
Significance of mean difference in weight between high‐fat diet and standard diet groups. The solid line shown is the mean difference in weight between the diet groups over time. The dashed line indicates 0 (i.e., no difference in weight). The polygon represents the confidence interval for the difference in the weights across time. The polygon turns red when the mean difference in weight between the diet groups becomes significantly different from 0

A significant difference between the HFD and SD groups was found in all physiological variables (Table 3). However, the timing of significant differences between groups varied depending on the response variable under study. For instance, there was a non‐significant difference in weight until Day 11 of the study, at which point the HFD was 8.4 g heavier than the SD group (*p *< 0.05). As the study progressed, the difference in weights between these groups continued to increase, ultimately reaching a difference of 37.6 g by Day 38 of the study (*p* < 0.01; Figure 1). Differences in glucose did not emerge between the two groups until Week 5 of the study and persisted through Week 6. In contrast, time was not a significant factor in explaining variation in ketone levels since the levels were consistently higher in the SD group than the HFD group throughout the study. Neither the weekly glucose nor the ketones exhibited a quadratic function over time.

**TABLE 3:** Diet group‐by‐time interactions

Units	Weight gain		Glucose		Ketones		Total time with novel object		Time ratio of novel object to familiar object	
	(g)		(g/dl)		(mmol/L)		(sec)		(unitless)	
Model term	*F*	*p*	*F*	*p*	*F*	*p*	*F*	*p*	*F*	*p*
Diet group	0.5	0.49^a^	7.6	0.01	4.7	0.03	0.8	0.39	2.9	0.1
Time	751.1	<0.01	1.3	0.25^a^	5.6	0.02	12.6	<0.01	16.5	<0.01
Diet group‐by‐time interaction	31.8	<0.01	10	<0.01	0	0.88	1	0.39	1.6	0.2
Time^2^	151.8	<0.01	NA^b^	NA	NA	NA	NA	NA	NA	NA

^a^ Though the main effect of the diet group appears to be nonsignificant, the diet group‐by‐time interaction is highly significant, indicating that the two diet groups respond differently over time.

^b^ NA indicates that the quadratic effect of time was not significant.

### 3.2 Behavioral characteristics

The OF distance was not significantly different between the two groups (*p* = 0.12), nor were there significant differences between the HFD and SD groups both in terms of the total amount of time spent and the ratio of the time spent with the novel object in comparison to the familiar object. Although there were no differences between the SD and HFD groups in the NOR study, there were significant differences between the study days. When examining the ratio of the time spent with the novel object in comparison to the familiar object, individuals in all groups, on average, spent significantly more time with the novel object than the familiar object on Day 0 than Day 1 (*p* < 0.05), on Day 3 compared to Day 1 (*p *< 0.01), and on Day 3 compared to Day 7 (*p *< 0.01). When examining the total time spent with the novel object, individuals spent significantly more time with the novel object on Day 3 compared to any of the other days (*p *< 0.01), averaging a difference of 32.6 s; individuals also spent significantly more time with the novel object on Day 7 as compared to Day 1 (*p *< 0.05), averaging a difference of 5.3 s.

### 3.3 Correlation and discriminant analysis

**FIGURE 2:**
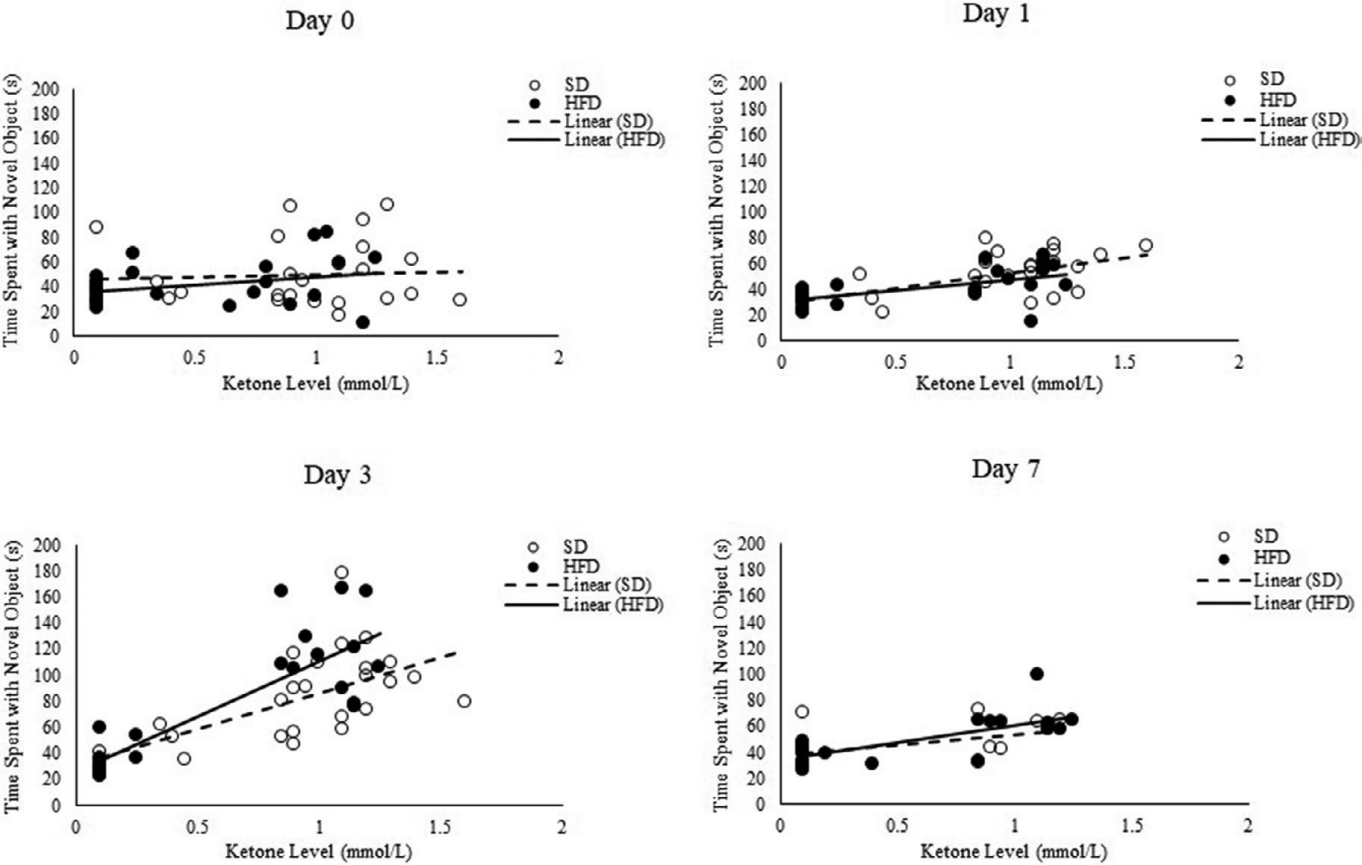
This figure shows the amount of time spent with the novel object (*Y*‐axis) against ketone levels (*X*‐axis); HFD: high‐fat diet; SD: standard diet; s: seconds; mmol/L: millimoles per liter

A summary of all correlations can be found in Table 4. Notably, in both the HFD and SD groups, ketone concentrations were positively correlated with the amount of time spent with the novel object in the NOR test (*r* = 0.57 for SD and 0.62 for HFD). Therefore, although the difference between SD and HFD groups in the NOR test was non‐significant, this correlation suggests that diet may be related to mental fatigue by altering ketone levels. Further studies must be conducted to elucidate causal mechanisms that relate to these two factors. Indeed, an ANOVA which replaced the diet factor with a term that categorized individuals based on above/below median ketone levels indicated that ketone concentrations were significantly associated with the longer time spent with the novel object (*p* < 0.01; Figure 2).

**TABLE 4:** Correlations between diet group and variables

	Weight	Glucose	Ketones	Open field distance	Total time with novel object	Time ratio of novel object to familiar object
Weight		0.32	−0.19	−0.02	−0.16	0.001
Glucose	−0.08		0.16	0.23	−0.21	−0.42
Ketones	0.08	0.06		0.44	0.62	0.18
Open field distance	−0.11	0.11	0.33		−0.28	−0.2
Total time with novel object	−0.01	0.05	0.57	0.65		0.62
Time ratio of novel object to familiar object	0.35	−0.07	0.47	−0.07	0.62	

Note

Table 4 shows the correlation between variables by diet group. The HFD group is shown in the upper triangle, whereas the SD group is shown in the lower triangle.

Abbreviations: HFD, high‐fat diet; SD, standard diet.

Furthermore, these differences were most pronounced at Day 3 (difference of 53.1 s on average, *p* < 0.01; Figure 2). Ketone concentrations were also moderately correlated with the OF distance traveled in both groups, but other correlations, such as weight and glucose, were group dependent. A linear discriminant analysis (LDA) was used to understand specific variables that drove differences between the SD and HFD groups. Of the variables measured, ketone concentrations and weight drove these classifications, with the HFD group having a higher weight and lower ketone concentration on average and the SD group having the opposite characteristic. The LDA correctly classified individuals into their respective dietary groups with an accuracy of 82%. Therefore, this analysis elucidates the direct effect of diet not only on weight but also on ketone concentrations. Further, of all physiological variables measured as part of this study, the ketones were most correlated with the behavioral variables.

## 4 DISCUSSION

This study was designed to measure the effect of 60% HFD‐induced obesity and the likelihood of experiencing the symptoms of physical and mental fatigue via decreased locomotor activity and impaired cognitions expected, 6 weeks of being fed an HFD caused a significant increase in body mass starting at Day 11 and higher glucose levels starting at Week 5, thus confirming that 60% kcal from fat being fed for 6 weeks was capable of inducing obesity. Our findings on changes in the body mass and blood glucose levels are similar to other studies (Ghibaudi et al., 2002; Marques et al., 2016). Ghibaudi et al. demonstrated a significant increase in body mass on a 45% HFD after 7 weeks of the diet. Higher plasma glucose levels were also seen in the same study with HFD‐fed rats after 12 weeks of this regimen (Ghibaudi et al., 2002). Another study by Marques et al. utilized an HFD consisting of 40% energy from fat over 17 weeks and reported a significant weight gain and hyperglycemia in Wistar and Sprague Dawley rats (Marques et al., 2016).

Ketone levels were significantly higher in the SD group compared to the HFD group throughout the study period. The correlation analyses revealed that ketone levels were moderately associated with the traveled distance measured through OF testing with no significant difference between the two groups. Further, during NOR testing, higher ketone levels were associated with significantly more time with the novel object. Hernandez et al. (2018) reported similar findings using a KD to demonstrate that rats with higher ketone levels performed better during dual working memory/ bi‐conditional association task (Hernandez et al., 2018). Overall, these results suggest that higher ketone levels are associated with better cognitive performance regardless of diet, indicating a reduced likelihood of experiencing mental fatigue.

Diet formulation should be high in fats, moderate in proteins, and very low in carbohydrates to achieve ketosis (Masood et al., 2020). Specifically, carbohydrates need to be significantly limited at 5%–10% kcal and protein levels should be between 30% and 35% kcal (Masood et al., 2020). The HFD used in this study contained 60% kcal from fat, 20% kcal from carbohydrates, and 20% kcal from proteins. Though fat content was sufficient in the HFD used in this study to induce obesity, the presence of carbohydrates at 20% kcal was presumably sufficient to prevent ketosis.

The type of fat used in the HFD and SD diets could have led to different ketone levels in the present study. While the HFD used lard and soybean oil, the SD used polyunsaturated fatty acids. Each lipid type produces differing phenotypic variations (Gajda, 2008). In a study by Buettner et al. (2007), a 42% kcal fat HFD was fed to male Wistar rats, with each diet containing differing fat sources including lard, olive oil, coconut fat, and fish oil over 12 weeks. This study found that the characteristic effects of an HFD, including weight gain and increased glucose and ketone levels, are most associated with HFDs that utilize saturated fats such as those with lard or olive oil. Free fatty acids in the plasma were elevated only in the olive oil, lard, and coconut oil groups, whereas the fish oil group did not differ from controls (Buettner et al., 2007). These differences can affect body weight and glucose concentrations as well. Ikemoto et al. (1996) evaluated different HFDs containing palm, lard, rapeseed, soybean, fish, perilla, and safflower oils. Each HFD contained 60% kcal fat and was fed to mice over 19 weeks. Bodyweight gain was greatest in the soybean oil group and least significant in the fish oil group. Glucose levels 30 min post‐glucose loading were highest in the safflower oil group and lowest in the palm oil group suggesting the possibility of variation based on differing lipids (Ikemoto et al., 1996).

Regardless of the type of diet, the rats with higher ketone concentrations were associated with greater time spent with the novel versus a familiar object in NOR, with more time spent with the novel object suggesting improved cognition. This association between higher ketone levels and improved cognition is consistent with previous studies (Maalouf et al., 2009). In a study by Murray et al. (2016), male rats were fed a high ketone ester diet (standard chow diet supplemented with (R)‐3‐hydroxybutyl (R)‐3‐hydroxybutyrate as 30% of calories) for 40 days. After 36 days of receiving the ketone diet, rats underwent radial arm maze testing as a measure of memory. Rats fed the ketone diet traveled through the maze at a rate of 38% faster with fewer mistakes than rats being fed all other diet types, including the Western diet, standard chow, or a high carbohydrate diet. Plasma ꞵ‐hydroxybutyrate levels were significantly higher in rats being fed the ketone diet versus all other groups (Murray et al., 2016). Several theories discuss how ketones exert such effects. For instance, the neuroprotective properties of the ketone bodies (Maalouf et al., 2009) might reduce neuronal apoptosis and brain edema and increased levels of neurotrophins as a way of positively impacting cognitive function (Kramer & Bressan, 2018). Despite the many possible mechanisms responsible for this positive effect, increased ketone levels have a direct role in enhancing brain function and thus preventing mental fatigue. Our findings support this because rats with lower ketone levels and increased body weight performed worse during NOR, suggesting mental fatigue, whereas rats with higher ketone levels had a greater performance, indicating resistance to mental fatigue.

Regardless of the treatment group, rats with higher ketone levels generally traveled a greater distance during OF, as shown by the moderate association of ketones found during correlation analyses, which is consistent with others’ findings. A study by Nozawa et al. (2009) displayed the benefits of increased ketone levels in combating physical fatigue. Mice exposed to bonito extract, a substance that increases ketone levels, were put through a forced swimming test and forced walking model to test for physical fatigue. Mice on bonito extract exhibited resistance to physical fatigue during testing due to increased ketone levels (Nozawa et al., 2009). This finding was reiterated in the study by Murray et al. (2016), which utilized a treadmill test that gradually increased the speed at which the treadmill ran until the rats were fatigued and could no longer maintain the pace of the belt as a measure of physical fatigue. Just as before, the rats fed a 30% ketone diet exhibited higher plasma ketone levels and ran 32% further than control rats (Murray et al., 2016). Thus, the positive effects of higher ketone levels appear to reduce the likelihood of experiencing mental fatigue and physical fatigue.

High‐fat diet led to a significant increase in body mass and glucose levels in the male Sprague Dawley rats. Increased ketone levels led to increased physical and mental performance during OF and NOR testing regardless of diet group, suggesting that adverse effects of obesity can potentially be mitigated if optimal ketone levels are achieved. Future studies combining lifestyle modifications to the HFD could shed light on the relationship between energy expenditure and physical and cognitive processes.

## DISCLOSURES

No conflicts of interest are stated for this study. This research did not receive any specific grant from funding agencies in the public, commercial, or not‐for‐profit sectors.

## AUTHOR CONTRIBUTIONS

Paige Niepoetter: animal work, data collection and analysis, study design, manuscript preparation. Carrie Butts‐Wilmsmeyer: data analysis and manuscript preparation. Sepideh Kaviani: manuscript preparation. Coral Viernow: animal work and data collection. Hannah Ruholl^:^ animal work and data collection. Chaya Gopalan: study design, animal work, data collection, data analysis, manuscript preparation.

## Supporting information



Supplementary Material
