# Supplementary material for: Correlation between ketones and mental fatigue in high fat‐induced obese and non‐obese rats
Source: Physiol Rep. 2021 Jul 1;9(13):e14930. doi: 10.14814/phy2.14930 (PMC8248918; doi:10.14814/phy2.14930)
Supplement: Supplementary file 1 — Supplementary Material [file PHY2-9-e14930-s001.doc]

| **Model Information** | |
| --- | --- |
| **Data Set** | WORK.NOR |
| **Dependent Variable** | transratio |
| **Covariance Structure** | Unstructured |
| **Subject Effect** | ID(Group) |
| **Estimation Method** | REML |
| **Residual Variance Method** | None |
| **Fixed Effects SE Method** | Model-Based |
| **Degrees of Freedom Method** | Between-Within |

| **Class Level Information** | | |
| --- | --- | --- |
| **Class** | **Levels** | **Values** |
| **Group** | 2 | HFD SD |
| **ID** | 48 | C1 C10 C11 C12 C13 C14 C15 C16 C17 C18 C19 C2 C20 C21 C22 C23 C24 C25 C26 C3 C4 C5 C6 C7 C8 C9 T1 T10 T11 T12 T13 T14 T15 T17 T2 T21 T22 T23 T24 T26 T27 T3 T4 T5 T6 T7 T8 T9 |
| **NORday** | 4 | 0 1 3 7 |

| **Dimensions** | |
| --- | --- |
| **Covariance Parameters** | 10 |
| **Columns in X** | 15 |
| **Columns in Z** | 0 |
| **Subjects** | 48 |
| **Max Obs per Subject** | 4 |

| **Number of Observations** | |
| --- | --- |
| **Number of Observations Read** | 192 |
| **Number of Observations Used** | 191 |
| **Number of Observations Not Used** | 1 |

| **Iteration History** | | | |
| --- | --- | --- | --- |
| **Iteration** | **Evaluations** | **-2 Res Log Like** | **Criterion** |
| **0** | 1 | 411.83460272 |  |
| **1** | 2 | 368.66206342 | 0.00002153 |
| **2** | 1 | 368.66171399 | 0.00000000 |

| Convergence criteria met. |
| --- |

| **Covariance Parameter Estimates** | | |
| --- | --- | --- |
| **Cov Parm** | **Subject** | **Estimate** |
| **UN(1,1)** | ID(Group) | 0.9269 |
| **UN(2,1)** | ID(Group) | -0.01245 |
| **UN(2,2)** | ID(Group) | 0.2123 |
| **UN(3,1)** | ID(Group) | 0.01255 |
| **UN(3,2)** | ID(Group) | -0.01831 |
| **UN(3,3)** | ID(Group) | 0.4755 |
| **UN(4,1)** | ID(Group) | 0.1419 |
| **UN(4,2)** | ID(Group) | 0.04323 |
| **UN(4,3)** | ID(Group) | 0.1848 |
| **UN(4,4)** | ID(Group) | 0.3379 |

| **Fit Statistics** | |
| --- | --- |
| **-2 Res Log Likelihood** | 368.7 |
| **AIC (Smaller is Better)** | 388.7 |
| **AICC (Smaller is Better)** | 389.9 |
| **BIC (Smaller is Better)** | 407.4 |

| **Null Model Likelihood Ratio Test** | | |
| --- | --- | --- |
| **DF** | **Chi-Square** | **Pr > ChiSq** |
| 9 | 43.17 | <.0001 |

| **Type 3 Tests of Fixed Effects** | | | | |
| --- | --- | --- | --- | --- |
| **Effect** | **Num DF** | **Den DF** | **F Value** | **Pr > F** |
| **Group** | 1 | 46 | 2.88 | 0.0963 |
| **NORday** | 3 | 46 | 16.51 | <.0001 |
| **Group*NORday** | 3 | 46 | 1.63 | 0.1956 |

| **Least Squares Means** | | | | | | | |
| --- | --- | --- | --- | --- | --- | --- | --- |
| **Effect** | **Group** | **NORday** | **Estimate** | **Standard Error** | **DF** | **t Value** | **Pr > |t|** |
| **NORday** |  | 0 | 0.4650 | 0.1406 | 46 | 3.31 | 0.0018 |
| **NORday** |  | 1 | 0.01538 | 0.06674 | 46 | 0.23 | 0.8188 |
| **NORday** |  | 3 | 0.7827 | 0.09988 | 46 | 7.84 | <.0001 |
| **NORday** |  | 7 | 0.2243 | 0.08420 | 46 | 2.66 | 0.0106 |
| **Group** | HFD |  | 0.2714 | 0.08687 | 46 | 3.12 | 0.0031 |
| **Group** | SD |  | 0.4723 | 0.08042 | 46 | 5.87 | <.0001 |

| **Differences of Least Squares Means** | | | | | | | | | | | |
| --- | --- | --- | --- | --- | --- | --- | --- | --- | --- | --- | --- |
| **Effect** | **Group** | **NORday** | **_Group** | **_NORday** | **Estimate** | **Standard Error** | **DF** | **t Value** | **Pr > |t|** | **Adjustment** | **Adj P** |
| **NORday** |  | 0 |  | 1 | 0.4496 | 0.1573 | 46 | 2.86 | 0.0064 | Tukey-Kramer | 0.0313 |
| **NORday** |  | 0 |  | 3 | -0.3177 | 0.1709 | 46 | -1.86 | 0.0695 | Tukey-Kramer | 0.2599 |
| **NORday** |  | 0 |  | 7 | 0.2406 | 0.1446 | 46 | 1.66 | 0.1029 | Tukey-Kramer | 0.3540 |
| **NORday** |  | 1 |  | 3 | -0.7673 | 0.1233 | 46 | -6.22 | <.0001 | Tukey-Kramer | <.0001 |
| **NORday** |  | 1 |  | 7 | -0.2089 | 0.09864 | 46 | -2.12 | 0.0396 | Tukey-Kramer | 0.1626 |
| **NORday** |  | 3 |  | 7 | 0.5584 | 0.09649 | 46 | 5.79 | <.0001 | Tukey-Kramer | <.0001 |
| **Group** | HFD |  | SD |  | -0.2010 | 0.1184 | 46 | -1.70 | 0.0963 |  | . |

| **Moments** | | | |
| --- | --- | --- | --- |
| **N** | 191 | **Sum Weights** | 191 |
| **Mean** | 0.0033626 | **Sum Observations** | 0.64225719 |
| **Std Deviation** | 0.68271505 | **Variance** | 0.46609984 |
| **Skewness** | 0.20038556 | **Kurtosis** | 0.51851161 |
| **Uncorrected SS** | 88.5611301 | **Corrected SS** | 88.5589705 |
| **Coeff Variation** | 20303.171 | **Std Error Mean** | 0.04939953 |

| **Basic Statistical Measures** | | | |
| --- | --- | --- | --- |
| **Location** | | **Variability** | |
| **Mean** | 0.00336 | **Std Deviation** | 0.68272 |
| **Median** | -0.00917 | **Variance** | 0.46610 |
| **Mode** | . | **Range** | 4.22524 |
|  |  | **Interquartile Range** | 0.84850 |

| **Tests for Location: Mu0=0** | | | | |
| --- | --- | --- | --- | --- |
| **Test** | **Statistic** | | **p Value** | |
| **Student's t** | **t** | 0.06807 | **Pr > |t|** | 0.9458 |
| **Sign** | **M** | -0.5 | **Pr >= |M|** | 1.0000 |
| **Signed Rank** | **S** | -92 | **Pr >= |S|** | 0.9046 |

| **Tests for Normality** | | | | |
| --- | --- | --- | --- | --- |
| **Test** | **Statistic** | | **p Value** | |
| **Shapiro-Wilk** | **W** | 0.993732 | **Pr < W** | 0.5971 |
| **Kolmogorov-Smirnov** | **D** | 0.038823 | **Pr > D** | >0.1500 |
| **Cramer-von Mises** | **W-Sq** | 0.046989 | **Pr > W-Sq** | >0.2500 |
| **Anderson-Darling** | **A-Sq** | 0.30352 | **Pr > A-Sq** | >0.2500 |

| **Quantiles (Definition 5)** | |
| --- | --- |
| **Level** | **Quantile** |
| **100% Max** | 2.18500081 |
| **99%** | 1.93365384 |
| **95%** | 1.11390249 |
| **90%** | 0.80602138 |
| **75% Q3** | 0.41614085 |
| **50% Median** | -0.00916666 |
| **25% Q1** | -0.43236060 |
| **10%** | -0.85086297 |
| **5%** | -1.07803419 |
| **1%** | -1.57812843 |
| **0% Min** | -2.04023431 |

| **Extreme Observations** | | | |
| --- | --- | --- | --- |
| **Lowest** | | **Highest** | |
| **Value** | **Obs** | **Value** | **Obs** |
| -2.04023 | 29 | 1.49435 | 177 |
| -1.57813 | 137 | 1.66671 | 73 |
| -1.48126 | 37 | 1.84099 | 65 |
| -1.28142 | 59 | 1.93365 | 81 |
| -1.23433 | 104 | 2.18500 | 169 |

| **Missing Values** | | | |
| --- | --- | --- | --- |
| **Missing Value** | **Count** | **Percent Of** | |
|  |  | **All Obs** | **Missing Obs** |
| . | 1 | 0.52 | 100.00 |

| **Model Information** | |
| --- | --- |
| **Data Set** | WORK.NOR |
| **Dependent Variable** | timeA |
| **Covariance Structure** | Unstructured |
| **Subject Effect** | ID(Group) |
| **Estimation Method** | REML |
| **Residual Variance Method** | None |
| **Fixed Effects SE Method** | Model-Based |
| **Degrees of Freedom Method** | Between-Within |

| **Class Level Information** | | |
| --- | --- | --- |
| **Class** | **Levels** | **Values** |
| **Group** | 2 | HFD SD |
| **ID** | 48 | C1 C10 C11 C12 C13 C14 C15 C16 C17 C18 C19 C2 C20 C21 C22 C23 C24 C25 C26 C3 C4 C5 C6 C7 C8 C9 T1 T10 T11 T12 T13 T14 T15 T17 T2 T21 T22 T23 T24 T26 T27 T3 T4 T5 T6 T7 T8 T9 |
| **NORday** | 4 | 0 1 3 7 |

| **Dimensions** | |
| --- | --- |
| **Covariance Parameters** | 10 |
| **Columns in X** | 16 |
| **Columns in Z** | 0 |
| **Subjects** | 48 |
| **Max Obs per Subject** | 4 |

| **Number of Observations** | |
| --- | --- |
| **Number of Observations Read** | 192 |
| **Number of Observations Used** | 176 |
| **Number of Observations Not Used** | 16 |

| **Iteration History** | | | |
| --- | --- | --- | --- |
| **Iteration** | **Evaluations** | **-2 Res Log Like** | **Criterion** |
| **0** | 1 | 1456.41653015 |  |
| **1** | 2 | 1423.42128061 | 0.00011149 |
| **2** | 1 | 1423.35608108 | 0.00000072 |
| **3** | 1 | 1423.35567491 | 0.00000000 |

| Convergence criteria met. |
| --- |

| **Covariance Parameter Estimates** | | |
| --- | --- | --- |
| **Cov Parm** | **Subject** | **Estimate** |
| **UN(1,1)** | ID(Group) | 384.91 |
| **UN(2,1)** | ID(Group) | 119.00 |
| **UN(2,2)** | ID(Group) | 331.76 |
| **UN(3,1)** | ID(Group) | 85.1026 |
| **UN(3,2)** | ID(Group) | 51.9723 |
| **UN(3,3)** | ID(Group) | 143.99 |
| **UN(4,1)** | ID(Group) | 111.01 |
| **UN(4,2)** | ID(Group) | 78.6040 |
| **UN(4,3)** | ID(Group) | 121.48 |
| **UN(4,4)** | ID(Group) | 404.27 |

| **Fit Statistics** | |
| --- | --- |
| **-2 Res Log Likelihood** | 1423.4 |
| **AIC (Smaller is Better)** | 1443.4 |
| **AICC (Smaller is Better)** | 1444.8 |
| **BIC (Smaller is Better)** | 1462.1 |

| **Null Model Likelihood Ratio Test** | | |
| --- | --- | --- |
| **DF** | **Chi-Square** | **Pr > ChiSq** |
| 9 | 33.06 | 0.0001 |

| **Type 3 Tests of Fixed Effects** | | | | |
| --- | --- | --- | --- | --- |
| **Effect** | **Num DF** | **Den DF** | **F Value** | **Pr > F** |
| **Group** | 1 | 46 | 1.48 | 0.2304 |
| **NORday** | 3 | 46 | 16.00 | <.0001 |
| **Group*NORday** | 3 | 46 | 2.20 | 0.1011 |
| **wk_ketone** | 1 | 46 | 2.50 | 0.1208 |

| **Least Squares Means** | | | | | | |
| --- | --- | --- | --- | --- | --- | --- |
| **Effect** | **NORday** | **Estimate** | **Standard Error** | **DF** | **t Value** | **Pr > |t|** |
| **NORday** | 0 | 32.1732 | 2.8417 | 46 | 11.32 | <.0001 |
| **NORday** | 1 | 46.7676 | 2.6409 | 46 | 17.71 | <.0001 |
| **NORday** | 3 | 33.0683 | 1.7422 | 46 | 18.98 | <.0001 |
| **NORday** | 7 | 48.1174 | 3.4827 | 46 | 13.82 | <.0001 |

| **Differences of Least Squares Means** | | | | | | | |
| --- | --- | --- | --- | --- | --- | --- | --- |
| **Effect** | **NORday** | **_NORday** | **Estimate** | **Standard Error** | **DF** | **t Value** | **Pr > |t|** |
| **NORday** | 0 | 1 | -14.5943 | 3.1713 | 46 | -4.60 | <.0001 |
| **NORday** | 0 | 3 | -0.8950 | 2.7459 | 46 | -0.33 | 0.7459 |
| **NORday** | 0 | 7 | -15.9442 | 3.9429 | 46 | -4.04 | 0.0002 |
| **NORday** | 1 | 3 | 13.6993 | 2.7929 | 46 | 4.91 | <.0001 |
| **NORday** | 1 | 7 | -1.3499 | 3.9768 | 46 | -0.34 | 0.7358 |
| **NORday** | 3 | 7 | -15.0492 | 3.1744 | 46 | -4.74 | <.0001 |

| **Moments** | | | |
| --- | --- | --- | --- |
| **N** | 176 | **Sum Weights** | 176 |
| **Mean** | 0.35695185 | **Sum Observations** | 62.8235255 |
| **Std Deviation** | 17.1813717 | **Variance** | 295.199533 |
| **Skewness** | 0.49335019 | **Kurtosis** | -0.2198316 |
| **Uncorrected SS** | 51682.3433 | **Corrected SS** | 51659.9183 |
| **Coeff Variation** | 4813.35836 | **Std Error Mean** | 1.29509462 |

| **Basic Statistical Measures** | | | |
| --- | --- | --- | --- |
| **Location** | | **Variability** | |
| **Mean** | 0.35695 | **Std Deviation** | 17.18137 |
| **Median** | -2.10332 | **Variance** | 295.19953 |
| **Mode** | . | **Range** | 83.07026 |
|  |  | **Interquartile Range** | 23.09500 |

| **Tests for Location: Mu0=0** | | | | |
| --- | --- | --- | --- | --- |
| **Test** | **Statistic** | | **p Value** | |
| **Student's t** | **t** | 0.275618 | **Pr > |t|** | 0.7832 |
| **Sign** | **M** | -8 | **Pr >= |M|** | 0.2581 |
| **Signed Rank** | **S** | -217 | **Pr >= |S|** | 0.7495 |

| **Tests for Normality** | | | | |
| --- | --- | --- | --- | --- |
| **Test** | **Statistic** | | **p Value** | |
| **Shapiro-Wilk** | **W** | 0.974743 | **Pr < W** | 0.0027 |
| **Kolmogorov-Smirnov** | **D** | 0.101489 | **Pr > D** | <0.0100 |
| **Cramer-von Mises** | **W-Sq** | 0.214098 | **Pr > W-Sq** | <0.0050 |
| **Anderson-Darling** | **A-Sq** | 1.229286 | **Pr > A-Sq** | <0.0050 |

| **Quantiles (Definition 5)** | |
| --- | --- |
| **Level** | **Quantile** |
| **100% Max** | 52.54100 |
| **99%** | 46.49890 |
| **95%** | 32.43097 |
| **90%** | 25.69396 |
| **75% Q3** | 11.67131 |
| **50% Median** | -2.10332 |
| **25% Q1** | -11.42368 |
| **10%** | -20.90604 |
| **5%** | -25.45258 |
| **1%** | -29.50385 |
| **0% Min** | -30.52926 |

| **Extreme Observations** | | | |
| --- | --- | --- | --- |
| **Lowest** | | **Highest** | |
| **Value** | **Obs** | **Value** | **Obs** |
| -30.5293 | 8 | 34.4906 | 37 |
| -29.5038 | 141 | 36.0179 | 21 |
| -28.8466 | 78 | 41.7339 | 103 |
| -28.6598 | 81 | 46.4989 | 60 |
| -28.6060 | 65 | 52.5410 | 25 |

| **Missing Values** | | | |
| --- | --- | --- | --- |
| **Missing Value** | **Count** | **Percent Of** | |
|  |  | **All Obs** | **Missing Obs** |
| . | 16 | 8.33 | 100.00 |

| **Model Information** | |
| --- | --- |
| **Data Set** | WORK.NOR |
| **Dependent Variable** | timeX |
| **Covariance Structure** | Unstructured |
| **Subject Effect** | ID(Group) |
| **Estimation Method** | REML |
| **Residual Variance Method** | None |
| **Fixed Effects SE Method** | Model-Based |
| **Degrees of Freedom Method** | Between-Within |

| **Class Level Information** | | |
| --- | --- | --- |
| **Class** | **Levels** | **Values** |
| **Group** | 2 | HFD SD |
| **ID** | 48 | C1 C10 C11 C12 C13 C14 C15 C16 C17 C18 C19 C2 C20 C21 C22 C23 C24 C25 C26 C3 C4 C5 C6 C7 C8 C9 T1 T10 T11 T12 T13 T14 T15 T17 T2 T21 T22 T23 T24 T26 T27 T3 T4 T5 T6 T7 T8 T9 |
| **NORday** | 4 | 0 1 3 7 |

| **Dimensions** | |
| --- | --- |
| **Covariance Parameters** | 10 |
| **Columns in X** | 15 |
| **Columns in Z** | 0 |
| **Subjects** | 48 |
| **Max Obs per Subject** | 4 |

| **Number of Observations** | |
| --- | --- |
| **Number of Observations Read** | 192 |
| **Number of Observations Used** | 191 |
| **Number of Observations Not Used** | 1 |

| **Iteration History** | | | |
| --- | --- | --- | --- |
| **Iteration** | **Evaluations** | **-2 Res Log Like** | **Criterion** |
| **0** | 1 | 1745.68008312 |  |
| **1** | 2 | 1644.44842080 | 0.00000000 |

| Convergence criteria met. |
| --- |

| **Covariance Parameter Estimates** | | |
| --- | --- | --- |
| **Cov Parm** | **Subject** | **Estimate** |
| **UN(1,1)** | ID(Group) | 534.39 |
| **UN(2,1)** | ID(Group) | 46.6156 |
| **UN(2,2)** | ID(Group) | 237.43 |
| **UN(3,1)** | ID(Group) | 269.84 |
| **UN(3,2)** | ID(Group) | 234.86 |
| **UN(3,3)** | ID(Group) | 1796.59 |
| **UN(4,1)** | ID(Group) | 112.47 |
| **UN(4,2)** | ID(Group) | 105.03 |
| **UN(4,3)** | ID(Group) | 403.16 |
| **UN(4,4)** | ID(Group) | 259.35 |

| **Fit Statistics** | |
| --- | --- |
| **-2 Res Log Likelihood** | 1644.4 |
| **AIC (Smaller is Better)** | 1664.4 |
| **AICC (Smaller is Better)** | 1665.7 |
| **BIC (Smaller is Better)** | 1683.2 |

| **Null Model Likelihood Ratio Test** | | |
| --- | --- | --- |
| **DF** | **Chi-Square** | **Pr > ChiSq** |
| 9 | 101.23 | <.0001 |

| **Type 3 Tests of Fixed Effects** | | | | |
| --- | --- | --- | --- | --- |
| **Effect** | **Num DF** | **Den DF** | **F Value** | **Pr > F** |
| **Group** | 1 | 46 | 0.76 | 0.3893 |
| **NORday** | 3 | 46 | 12.60 | <.0001 |
| **Group*NORday** | 3 | 46 | 1.02 | 0.3909 |

| **Least Squares Means** | | | | | | |
| --- | --- | --- | --- | --- | --- | --- |
| **Effect** | **NORday** | **Estimate** | **Standard Error** | **DF** | **t Value** | **Pr > |t|** |
| **NORday** | 0 | 46.2440 | 3.3756 | 46 | 13.70 | <.0001 |
| **NORday** | 1 | 46.0299 | 2.2318 | 46 | 20.62 | <.0001 |
| **NORday** | 3 | 80.4636 | 6.1393 | 46 | 13.11 | <.0001 |
| **NORday** | 7 | 51.3226 | 2.3326 | 46 | 22.00 | <.0001 |

| **Differences of Least Squares Means** | | | | | | | |
| --- | --- | --- | --- | --- | --- | --- | --- |
| **Effect** | **NORday** | **_NORday** | **Estimate** | **Standard Error** | **DF** | **t Value** | **Pr > |t|** |
| **NORday** | 0 | 1 | 0.2141 | 3.7973 | 46 | 0.06 | 0.9553 |
| **NORday** | 0 | 3 | -34.2197 | 6.1452 | 46 | -5.57 | <.0001 |
| **NORday** | 0 | 7 | -5.0786 | 3.4809 | 46 | -1.46 | 0.1514 |
| **NORday** | 1 | 3 | -34.4337 | 5.7287 | 46 | -6.01 | <.0001 |
| **NORday** | 1 | 7 | -5.2927 | 2.4525 | 46 | -2.16 | 0.0362 |
| **NORday** | 3 | 7 | 29.1411 | 5.1201 | 46 | 5.69 | <.0001 |

| **Analysis Variable : wk_ketone** | | |
| --- | --- | --- |
| **Minimum** | **Median** | **Maximum** |
| 0.1000000 | 0.9000000 | 1.6000000 |

| **Obs** | **ID** | **_TYPE_** | **_FREQ_** | **_STAT_** | **wk_ketone** |
| --- | --- | --- | --- | --- | --- |
| **1** | C1 | 0 | 4 | MEAN | 1.0625 |
| **2** | C10 | 0 | 4 | MEAN | 0.2875 |
| **3** | C11 | 0 | 4 | MEAN | 0.1000 |
| **4** | C12 | 0 | 4 | MEAN | 0.3625 |
| **5** | C13 | 0 | 4 | MEAN | 1.1000 |
| **6** | C14 | 0 | 4 | MEAN | 1.6000 |
| **7** | C15 | 0 | 4 | MEAN | 1.3000 |
| **8** | C16 | 0 | 4 | MEAN | 1.1000 |
| **9** | C17 | 0 | 4 | MEAN | 1.4000 |
| **10** | C18 | 0 | 4 | MEAN | 1.0000 |
| **11** | C19 | 0 | 4 | MEAN | 1.1000 |
| **12** | C2 | 0 | 4 | MEAN | 0.9875 |
| **13** | C20 | 0 | 4 | MEAN | 0.9000 |
| **14** | C21 | 0 | 4 | MEAN | 1.3000 |
| **15** | C22 | 0 | 4 | MEAN | 0.9000 |
| **16** | C23 | 0 | 4 | MEAN | 1.2000 |
| **17** | C24 | 0 | 4 | MEAN | 1.2000 |
| **18** | C25 | 0 | 4 | MEAN | 0.9000 |
| **19** | C26 | 0 | 4 | MEAN | 1.2000 |
| **20** | C3 | 0 | 4 | MEAN | 1.0625 |
| **21** | C4 | 0 | 4 | MEAN | 0.8500 |
| **22** | C5 | 0 | 4 | MEAN | 0.8500 |
| **23** | C6 | 0 | 4 | MEAN | 1.1125 |
| **24** | C7 | 0 | 4 | MEAN | 0.1000 |
| **25** | C8 | 0 | 4 | MEAN | 0.1000 |
| **26** | C9 | 0 | 4 | MEAN | 0.3250 |
| **27** | T1 | 0 | 4 | MEAN | 1.0875 |
| **28** | T10 | 0 | 4 | MEAN | 0.8625 |
| **29** | T11 | 0 | 4 | MEAN | 0.1750 |
| **30** | T12 | 0 | 4 | MEAN | 0.1000 |
| **31** | T13 | 0 | 4 | MEAN | 0.2125 |
| **32** | T14 | 0 | 4 | MEAN | 0.1000 |
| **33** | T15 | 0 | 4 | MEAN | 0.1000 |
| **34** | T17 | 0 | 4 | MEAN | 0.2125 |
| **35** | T2 | 0 | 4 | MEAN | 0.8375 |
| **36** | T21 | 0 | 4 | MEAN | 0.1250 |
| **37** | T22 | 0 | 4 | MEAN | 0.1000 |
| **38** | T23 | 0 | 4 | MEAN | 0.1000 |
| **39** | T24 | 0 | 4 | MEAN | 0.1000 |
| **40** | T26 | 0 | 4 | MEAN | 1.0000 |
| **41** | T27 | 0 | 4 | MEAN | 1.1000 |
| **42** | T3 | 0 | 4 | MEAN | 0.9000 |
| **43** | T4 | 0 | 4 | MEAN | 1.1125 |
| **44** | T5 | 0 | 4 | MEAN | 1.1000 |
| **45** | T6 | 0 | 4 | MEAN | 0.9875 |
| **46** | T7 | 0 | 4 | MEAN | 1.0625 |
| **47** | T8 | 0 | 4 | MEAN | 1.1625 |
| **48** | T9 | 0 | 4 | MEAN | 0.9875 |

| **Analysis Variable : wk_ketone** | | |
| --- | --- | --- |
| **Minimum** | **Median** | **Maximum** |
| 0.1000000 | 0.9437500 | 1.6000000 |

| **Model Information** | |
| --- | --- |
| **Data Set** | WORK.NOR2 |
| **Dependent Variable** | timeX |
| **Covariance Structure** | Unstructured |
| **Subject Effect** | ID(Group) |
| **Estimation Method** | REML |
| **Residual Variance Method** | None |
| **Fixed Effects SE Method** | Model-Based |
| **Degrees of Freedom Method** | Between-Within |

| **Class Level Information** | | |
| --- | --- | --- |
| **Class** | **Levels** | **Values** |
| **Group** | 2 | HFD SD |
| **ID** | 48 | C1 C10 C11 C12 C13 C14 C15 C16 C17 C18 C19 C2 C20 C21 C22 C23 C24 C25 C26 C3 C4 C5 C6 C7 C8 C9 T1 T10 T11 T12 T13 T14 T15 T17 T2 T21 T22 T23 T24 T26 T27 T3 T4 T5 T6 T7 T8 T9 |
| **NORday** | 4 | 0 1 3 7 |
| **ketone_group** | 2 | High Low |

| **Dimensions** | |
| --- | --- |
| **Covariance Parameters** | 10 |
| **Columns in X** | 29 |
| **Columns in Z** | 0 |
| **Subjects** | 48 |
| **Max Obs per Subject** | 4 |

| **Number of Observations** | |
| --- | --- |
| **Number of Observations Read** | 192 |
| **Number of Observations Used** | 191 |
| **Number of Observations Not Used** | 1 |

| **Iteration History** | | | |
| --- | --- | --- | --- |
| **Iteration** | **Evaluations** | **-2 Res Log Like** | **Criterion** |
| **0** | 1 | 1655.03746095 |  |
| **1** | 2 | 1587.95961257 | 0.00000015 |
| **2** | 1 | 1587.95951940 | 0.00000000 |

| Convergence criteria met. |
| --- |

| **Covariance Parameter Estimates** | | |
| --- | --- | --- |
| **Cov Parm** | **Subject** | **Estimate** |
| **UN(1,1)** | ID(Group) | 539.82 |
| **UN(2,1)** | ID(Group) | 40.0344 |
| **UN(2,2)** | ID(Group) | 205.52 |
| **UN(3,1)** | ID(Group) | 239.84 |
| **UN(3,2)** | ID(Group) | 70.4447 |
| **UN(3,3)** | ID(Group) | 1119.44 |
| **UN(4,1)** | ID(Group) | 99.4679 |
| **UN(4,2)** | ID(Group) | 58.1874 |
| **UN(4,3)** | ID(Group) | 215.80 |
| **UN(4,4)** | ID(Group) | 203.42 |

| **Fit Statistics** | |
| --- | --- |
| **-2 Res Log Likelihood** | 1588.0 |
| **AIC (Smaller is Better)** | 1608.0 |
| **AICC (Smaller is Better)** | 1609.3 |
| **BIC (Smaller is Better)** | 1626.7 |

| **Null Model Likelihood Ratio Test** | | |
| --- | --- | --- |
| **DF** | **Chi-Square** | **Pr > ChiSq** |
| 9 | 67.08 | <.0001 |

| **Type 3 Tests of Fixed Effects** | | | | |
| --- | --- | --- | --- | --- |
| **Effect** | **Num DF** | **Den DF** | **F Value** | **Pr > F** |
| **Group** | 1 | 44 | 0.05 | 0.8324 |
| **ketone_group** | 1 | 44 | 22.93 | <.0001 |
| **Group*ketone_group** | 1 | 44 | 2.25 | 0.1410 |
| **NORday** | 3 | 44 | 18.89 | <.0001 |
| **Group*NORday** | 3 | 44 | 1.47 | 0.2368 |
| **NORday*ketone_group** | 3 | 44 | 8.71 | 0.0001 |

| **Least Squares Means** | | | | | | | |
| --- | --- | --- | --- | --- | --- | --- | --- |
| **Effect** | **ketone_group** | **NORday** | **Estimate** | **Standard Error** | **DF** | **t Value** | **Pr > |t|** |
| **NORday*ketone_group** | High | 0 | 47.9701 | 4.9072 | 44 | 9.78 | <.0001 |
| **NORday*ketone_group** | Low | 0 | 45.2389 | 4.7531 | 44 | 9.52 | <.0001 |
| **NORday*ketone_group** | High | 1 | 53.0696 | 3.0028 | 44 | 17.67 | <.0001 |
| **NORday*ketone_group** | Low | 1 | 39.9943 | 2.9346 | 44 | 13.63 | <.0001 |
| **NORday*ketone_group** | High | 3 | 107.79 | 6.9520 | 44 | 15.51 | <.0001 |
| **NORday*ketone_group** | Low | 3 | 54.6995 | 6.8432 | 44 | 7.99 | <.0001 |
| **NORday*ketone_group** | High | 7 | 59.0815 | 2.9877 | 44 | 19.77 | <.0001 |
| **NORday*ketone_group** | Low | 7 | 44.5875 | 2.9196 | 44 | 15.27 | <.0001 |

| **Differences of Least Squares Means** | | | | | | | | | |
| --- | --- | --- | --- | --- | --- | --- | --- | --- | --- |
| **Effect** | **ketone_group** | **NORday** | **_ketone_group** | **_NORday** | **Estimate** | **Standard Error** | **DF** | **t Value** | **Pr > |t|** |
| **NORday*ketone_group** | High | 0 | Low | 0 | 2.7312 | 6.8559 | 44 | 0.40 | 0.6923 |
| **NORday*ketone_group** | High | 0 | High | 1 | -5.0996 | 5.4149 | 44 | -0.94 | 0.3515 |
| **NORday*ketone_group** | High | 0 | Low | 1 | 7.9758 | 5.7117 | 44 | 1.40 | 0.1696 |
| **NORday*ketone_group** | High | 0 | High | 3 | -59.8215 | 7.1725 | 44 | -8.34 | <.0001 |
| **NORday*ketone_group** | High | 0 | Low | 3 | -6.7294 | 8.4274 | 44 | -0.80 | 0.4289 |
| **NORday*ketone_group** | High | 0 | High | 7 | -11.1115 | 4.9110 | 44 | -2.26 | 0.0287 |
| **NORday*ketone_group** | High | 0 | Low | 7 | 3.3825 | 5.7087 | 44 | 0.59 | 0.5565 |
| **NORday*ketone_group** | Low | 0 | High | 1 | -7.8307 | 5.6155 | 44 | -1.39 | 0.1702 |
| **NORday*ketone_group** | Low | 0 | Low | 1 | 5.2446 | 5.2745 | 44 | 0.99 | 0.3255 |
| **NORday*ketone_group** | Low | 0 | High | 3 | -62.5527 | 8.4277 | 44 | -7.42 | <.0001 |
| **NORday*ketone_group** | Low | 0 | Low | 3 | -9.4605 | 7.0233 | 44 | -1.35 | 0.1849 |
| **NORday*ketone_group** | Low | 0 | High | 7 | -13.8426 | 5.6123 | 44 | -2.47 | 0.0176 |
| **NORday*ketone_group** | Low | 0 | Low | 7 | 0.6514 | 4.7710 | 44 | 0.14 | 0.8920 |
| **NORday*ketone_group** | High | 1 | Low | 1 | 13.0753 | 4.2074 | 44 | 3.11 | 0.0033 |
| **NORday*ketone_group** | High | 1 | High | 3 | -54.7220 | 7.1368 | 44 | -7.67 | <.0001 |
| **NORday*ketone_group** | High | 1 | Low | 3 | -1.6298 | 7.4698 | 44 | -0.22 | 0.8283 |
| **NORday*ketone_group** | High | 1 | High | 7 | -6.0119 | 3.5475 | 44 | -1.69 | 0.0972 |
| **NORday*ketone_group** | High | 1 | Low | 7 | 8.4821 | 4.1812 | 44 | 2.03 | 0.0486 |
| **NORday*ketone_group** | Low | 1 | High | 3 | -67.7973 | 7.5428 | 44 | -8.99 | <.0001 |
| **NORday*ketone_group** | Low | 1 | Low | 3 | -14.7052 | 7.0366 | 44 | -2.09 | 0.0424 |
| **NORday*ketone_group** | Low | 1 | High | 7 | -19.0873 | 4.1809 | 44 | -4.57 | <.0001 |
| **NORday*ketone_group** | Low | 1 | Low | 7 | -4.5933 | 3.4977 | 44 | -1.31 | 0.1959 |
| **NORday*ketone_group** | High | 3 | Low | 3 | 53.0922 | 9.8007 | 44 | 5.42 | <.0001 |
| **NORday*ketone_group** | High | 3 | High | 7 | 48.7101 | 6.1918 | 44 | 7.87 | <.0001 |
| **NORday*ketone_group** | High | 3 | Low | 7 | 63.2041 | 7.5456 | 44 | 8.38 | <.0001 |
| **NORday*ketone_group** | Low | 3 | High | 7 | -4.3821 | 7.4725 | 44 | -0.59 | 0.5606 |
| **NORday*ketone_group** | Low | 3 | Low | 7 | 10.1119 | 6.1049 | 44 | 1.66 | 0.1048 |
| **NORday*ketone_group** | High | 7 | Low | 7 | 14.4940 | 4.1860 | 44 | 3.46 | 0.0012 |

| **Model Information** | |
| --- | --- |
| **Data Set** | WORK.NOR2 |
| **Dependent Variable** | timeX |
| **Covariance Structure** | Unstructured |
| **Subject Effect** | ID(gk) |
| **Estimation Method** | REML |
| **Residual Variance Method** | None |
| **Fixed Effects SE Method** | Model-Based |
| **Degrees of Freedom Method** | Between-Within |

| **Class Level Information** | | |
| --- | --- | --- |
| **Class** | **Levels** | **Values** |
| **gk** | 4 | HFD_High HFD_Low SD_High SD_Low |
| **ID** | 48 | C1 C10 C11 C12 C13 C14 C15 C16 C17 C18 C19 C2 C20 C21 C22 C23 C24 C25 C26 C3 C4 C5 C6 C7 C8 C9 T1 T10 T11 T12 T13 T14 T15 T17 T2 T21 T22 T23 T24 T26 T27 T3 T4 T5 T6 T7 T8 T9 |
| **NORday** | 4 | 0 1 3 7 |

| **Dimensions** | |
| --- | --- |
| **Covariance Parameters** | 10 |
| **Columns in X** | 25 |
| **Columns in Z** | 0 |
| **Subjects** | 48 |
| **Max Obs per Subject** | 4 |

| **Number of Observations** | |
| --- | --- |
| **Number of Observations Read** | 192 |
| **Number of Observations Used** | 191 |
| **Number of Observations Not Used** | 1 |

| **Iteration History** | | | |
| --- | --- | --- | --- |
| **Iteration** | **Evaluations** | **-2 Res Log Like** | **Criterion** |
| **0** | 1 | 1632.08442912 |  |
| **1** | 2 | 1565.25358352 | 0.00000000 |

| Convergence criteria met. |
| --- |

| **Covariance Parameter Estimates** | | |
| --- | --- | --- |
| **Cov Parm** | **Subject** | **Estimate** |
| **UN(1,1)** | ID(gk) | 549.35 |
| **UN(2,1)** | ID(gk) | 38.7153 |
| **UN(2,2)** | ID(gk) | 204.95 |
| **UN(3,1)** | ID(gk) | 242.88 |
| **UN(3,2)** | ID(gk) | 67.6290 |
| **UN(3,3)** | ID(gk) | 1140.87 |
| **UN(4,1)** | ID(gk) | 98.4272 |
| **UN(4,2)** | ID(gk) | 58.9465 |
| **UN(4,3)** | ID(gk) | 219.33 |
| **UN(4,4)** | ID(gk) | 203.17 |

| **Fit Statistics** | |
| --- | --- |
| **-2 Res Log Likelihood** | 1565.3 |
| **AIC (Smaller is Better)** | 1585.3 |
| **AICC (Smaller is Better)** | 1586.6 |
| **BIC (Smaller is Better)** | 1604.0 |

| **Null Model Likelihood Ratio Test** | | |
| --- | --- | --- |
| **DF** | **Chi-Square** | **Pr > ChiSq** |
| 9 | 66.83 | <.0001 |

| **Type 3 Tests of Fixed Effects** | | | | |
| --- | --- | --- | --- | --- |
| **Effect** | **Num DF** | **Den DF** | **F Value** | **Pr > F** |
| **gk** | 3 | 44 | 8.00 | 0.0002 |
| **NORday** | 3 | 44 | 17.83 | <.0001 |
| **gk*NORday** | 9 | 44 | 3.38 | 0.0031 |

| **Least Squares Means** | | | | | | | |
| --- | --- | --- | --- | --- | --- | --- | --- |
| **Effect** | **gk** | **NORday** | **Estimate** | **Standard Error** | **DF** | **t Value** | **Pr > |t|** |
| **gk*NORday** | HFD_High | 0 | 47.6444 | 7.8127 | 44 | 6.10 | <.0001 |
| **gk*NORday** | HFD_High | 1 | 50.0222 | 4.7720 | 44 | 10.48 | <.0001 |
| **gk*NORday** | HFD_High | 3 | 113.16 | 11.2589 | 44 | 10.05 | <.0001 |
| **gk*NORday** | HFD_High | 7 | 64.0222 | 4.7513 | 44 | 13.47 | <.0001 |
| **gk*NORday** | HFD_Low | 0 | 39.2615 | 6.5006 | 44 | 6.04 | <.0001 |
| **gk*NORday** | HFD_Low | 1 | 35.3692 | 3.9705 | 44 | 8.91 | <.0001 |
| **gk*NORday** | HFD_Low | 3 | 57.3462 | 9.3680 | 44 | 6.12 | <.0001 |
| **gk*NORday** | HFD_Low | 7 | 41.5923 | 3.9533 | 44 | 10.52 | <.0001 |
| **gk*NORday** | SD_High | 0 | 48.4762 | 6.2379 | 44 | 7.77 | <.0001 |
| **gk*NORday** | SD_High | 1 | 55.3333 | 3.6964 | 44 | 14.97 | <.0001 |
| **gk*NORday** | SD_High | 3 | 101.91 | 8.7211 | 44 | 11.69 | <.0001 |
| **gk*NORday** | SD_High | 7 | 54.8600 | 3.6804 | 44 | 14.91 | <.0001 |
| **gk*NORday** | SD_Low | 0 | 51.2727 | 7.0669 | 44 | 7.26 | <.0001 |
| **gk*NORday** | SD_Low | 1 | 44.3727 | 4.3164 | 44 | 10.28 | <.0001 |
| **gk*NORday** | SD_Low | 3 | 51.8909 | 10.1841 | 44 | 5.10 | <.0001 |
| **gk*NORday** | SD_Low | 7 | 47.8091 | 4.2977 | 44 | 11.12 | <.0001 |

| **Differences of Least Squares Means** | | | | | | | | | |
| --- | --- | --- | --- | --- | --- | --- | --- | --- | --- |
| **Effect** | **gk** | **NORday** | **_gk** | **_NORday** | **Estimate** | **Standard Error** | **DF** | **t Value** | **Pr > |t|** |
| **gk*NORday** | HFD_High | 0 | HFD_High | 1 | -2.3778 | 8.6722 | 44 | -0.27 | 0.7852 |
| **gk*NORday** | HFD_High | 0 | HFD_High | 3 | -65.5111 | 11.5684 | 44 | -5.66 | <.0001 |
| **gk*NORday** | HFD_High | 0 | HFD_High | 7 | -16.3778 | 7.8575 | 44 | -2.08 | 0.0430 |
| **gk*NORday** | HFD_High | 0 | HFD_Low | 0 | 8.3829 | 10.1635 | 44 | 0.82 | 0.4139 |
| **gk*NORday** | HFD_High | 0 | HFD_Low | 1 | 12.2752 | 8.7638 | 44 | 1.40 | 0.1683 |
| **gk*NORday** | HFD_High | 0 | HFD_Low | 3 | -9.7017 | 12.1983 | 44 | -0.80 | 0.4307 |
| **gk*NORday** | HFD_High | 0 | HFD_Low | 7 | 6.0521 | 8.7560 | 44 | 0.69 | 0.4931 |
| **gk*NORday** | HFD_High | 0 | SD_High | 0 | -0.8318 | 9.9975 | 44 | -0.08 | 0.9341 |
| **gk*NORday** | HFD_High | 0 | SD_High | 1 | -7.6889 | 8.6430 | 44 | -0.89 | 0.3785 |
| **gk*NORday** | HFD_High | 0 | SD_High | 3 | -54.2689 | 11.7088 | 44 | -4.63 | <.0001 |
| **gk*NORday** | HFD_High | 0 | SD_High | 7 | -7.2156 | 8.6362 | 44 | -0.84 | 0.4079 |
| **gk*NORday** | HFD_High | 0 | SD_Low | 0 | -3.6283 | 10.5347 | 44 | -0.34 | 0.7322 |
| **gk*NORday** | HFD_High | 0 | SD_Low | 1 | 3.2717 | 8.9258 | 44 | 0.37 | 0.7157 |
| **gk*NORday** | HFD_High | 0 | SD_Low | 3 | -4.2465 | 12.8356 | 44 | -0.33 | 0.7423 |
| **gk*NORday** | HFD_High | 0 | SD_Low | 7 | -0.1646 | 8.9168 | 44 | -0.02 | 0.9854 |
| **gk*NORday** | HFD_High | 1 | HFD_High | 3 | -63.1333 | 11.5977 | 44 | -5.44 | <.0001 |
| **gk*NORday** | HFD_High | 1 | HFD_High | 7 | -14.0000 | 5.6787 | 44 | -2.47 | 0.0177 |
| **gk*NORday** | HFD_High | 1 | HFD_Low | 0 | 10.7607 | 8.0641 | 44 | 1.33 | 0.1889 |
| **gk*NORday** | HFD_High | 1 | HFD_Low | 1 | 14.6530 | 6.2078 | 44 | 2.36 | 0.0228 |
| **gk*NORday** | HFD_High | 1 | HFD_Low | 3 | -7.3239 | 10.5134 | 44 | -0.70 | 0.4897 |
| **gk*NORday** | HFD_High | 1 | HFD_Low | 7 | 8.4299 | 6.1968 | 44 | 1.36 | 0.1806 |
| **gk*NORday** | HFD_High | 1 | SD_High | 0 | 1.5460 | 7.8539 | 44 | 0.20 | 0.8449 |
| **gk*NORday** | HFD_High | 1 | SD_High | 1 | -5.3111 | 6.0361 | 44 | -0.88 | 0.3837 |
| **gk*NORday** | HFD_High | 1 | SD_High | 3 | -51.8911 | 9.9413 | 44 | -5.22 | <.0001 |
| **gk*NORday** | HFD_High | 1 | SD_High | 7 | -4.8378 | 6.0263 | 44 | -0.80 | 0.4264 |
| **gk*NORday** | HFD_High | 1 | SD_Low | 0 | -1.2505 | 8.5272 | 44 | -0.15 | 0.8841 |
| **gk*NORday** | HFD_High | 1 | SD_Low | 1 | 5.6495 | 6.4345 | 44 | 0.88 | 0.3847 |
| **gk*NORday** | HFD_High | 1 | SD_Low | 3 | -1.8687 | 11.2466 | 44 | -0.17 | 0.8688 |
| **gk*NORday** | HFD_High | 1 | SD_Low | 7 | 2.2131 | 6.4220 | 44 | 0.34 | 0.7320 |
| **gk*NORday** | HFD_High | 3 | HFD_High | 7 | 49.1333 | 10.0299 | 44 | 4.90 | <.0001 |
| **gk*NORday** | HFD_High | 3 | HFD_Low | 0 | 73.8940 | 13.0008 | 44 | 5.68 | <.0001 |
| **gk*NORday** | HFD_High | 3 | HFD_Low | 1 | 77.7863 | 11.9385 | 44 | 6.52 | <.0001 |
| **gk*NORday** | HFD_High | 3 | HFD_Low | 3 | 55.8094 | 14.6466 | 44 | 3.81 | 0.0004 |
| **gk*NORday** | HFD_High | 3 | HFD_Low | 7 | 71.5632 | 11.9328 | 44 | 6.00 | <.0001 |
| **gk*NORday** | HFD_High | 3 | SD_High | 0 | 64.6794 | 12.8714 | 44 | 5.03 | <.0001 |
| **gk*NORday** | HFD_High | 3 | SD_High | 1 | 57.8222 | 11.8501 | 44 | 4.88 | <.0001 |
| **gk*NORday** | HFD_High | 3 | SD_High | 3 | 11.2422 | 14.2415 | 44 | 0.79 | 0.4341 |
| **gk*NORday** | HFD_High | 3 | SD_High | 7 | 58.2956 | 11.8452 | 44 | 4.92 | <.0001 |
| **gk*NORday** | HFD_High | 3 | SD_Low | 0 | 61.8828 | 13.2930 | 44 | 4.66 | <.0001 |
| **gk*NORday** | HFD_High | 3 | SD_Low | 1 | 68.7828 | 12.0580 | 44 | 5.70 | <.0001 |
| **gk*NORday** | HFD_High | 3 | SD_Low | 3 | 61.2646 | 15.1815 | 44 | 4.04 | 0.0002 |
| **gk*NORday** | HFD_High | 3 | SD_Low | 7 | 65.3465 | 12.0513 | 44 | 5.42 | <.0001 |
| **gk*NORday** | HFD_High | 7 | HFD_Low | 0 | 24.7607 | 8.0519 | 44 | 3.08 | 0.0036 |
| **gk*NORday** | HFD_High | 7 | HFD_Low | 1 | 28.6530 | 6.1919 | 44 | 4.63 | <.0001 |
| **gk*NORday** | HFD_High | 7 | HFD_Low | 3 | 6.6761 | 10.5040 | 44 | 0.64 | 0.5283 |
| **gk*NORday** | HFD_High | 7 | HFD_Low | 7 | 22.4299 | 6.1809 | 44 | 3.63 | 0.0007 |
| **gk*NORday** | HFD_High | 7 | SD_High | 0 | 15.5460 | 7.8413 | 44 | 1.98 | 0.0537 |
| **gk*NORday** | HFD_High | 7 | SD_High | 1 | 8.6889 | 6.0198 | 44 | 1.44 | 0.1560 |
| **gk*NORday** | HFD_High | 7 | SD_High | 3 | -37.8911 | 9.9314 | 44 | -3.82 | 0.0004 |
| **gk*NORday** | HFD_High | 7 | SD_High | 7 | 9.1622 | 6.0100 | 44 | 1.52 | 0.1345 |
| **gk*NORday** | HFD_High | 7 | SD_Low | 0 | 12.7495 | 8.5156 | 44 | 1.50 | 0.1415 |
| **gk*NORday** | HFD_High | 7 | SD_Low | 1 | 19.6495 | 6.4192 | 44 | 3.06 | 0.0038 |
| **gk*NORday** | HFD_High | 7 | SD_Low | 3 | 12.1313 | 11.2379 | 44 | 1.08 | 0.2862 |
| **gk*NORday** | HFD_High | 7 | SD_Low | 7 | 16.2131 | 6.4067 | 44 | 2.53 | 0.0150 |
| **gk*NORday** | HFD_Low | 0 | HFD_Low | 1 | 3.8923 | 7.2157 | 44 | 0.54 | 0.5923 |
| **gk*NORday** | HFD_Low | 0 | HFD_Low | 3 | -18.0846 | 9.6255 | 44 | -1.88 | 0.0669 |
| **gk*NORday** | HFD_Low | 0 | HFD_Low | 7 | -2.3308 | 6.5379 | 44 | -0.36 | 0.7232 |
| **gk*NORday** | HFD_Low | 0 | SD_High | 0 | -9.2147 | 9.0094 | 44 | -1.02 | 0.3120 |
| **gk*NORday** | HFD_Low | 0 | SD_High | 1 | -16.0718 | 7.4780 | 44 | -2.15 | 0.0372 |
| **gk*NORday** | HFD_Low | 0 | SD_High | 3 | -62.6518 | 10.8773 | 44 | -5.76 | <.0001 |
| **gk*NORday** | HFD_Low | 0 | SD_High | 7 | -15.5985 | 7.4701 | 44 | -2.09 | 0.0426 |
| **gk*NORday** | HFD_Low | 0 | SD_Low | 0 | -12.0112 | 9.6020 | 44 | -1.25 | 0.2176 |
| **gk*NORday** | HFD_Low | 0 | SD_Low | 1 | -5.1112 | 7.8031 | 44 | -0.66 | 0.5159 |
| **gk*NORday** | HFD_Low | 0 | SD_Low | 3 | -12.6294 | 12.0819 | 44 | -1.05 | 0.3016 |
| **gk*NORday** | HFD_Low | 0 | SD_Low | 7 | -8.5476 | 7.7928 | 44 | -1.10 | 0.2787 |
| **gk*NORday** | HFD_Low | 1 | HFD_Low | 3 | -21.9769 | 9.6499 | 44 | -2.28 | 0.0277 |
| **gk*NORday** | HFD_Low | 1 | HFD_Low | 7 | -6.2231 | 4.7250 | 44 | -1.32 | 0.1946 |
| **gk*NORday** | HFD_Low | 1 | SD_High | 0 | -13.1070 | 7.3944 | 44 | -1.77 | 0.0832 |
| **gk*NORday** | HFD_Low | 1 | SD_High | 1 | -19.9641 | 5.4248 | 44 | -3.68 | 0.0006 |
| **gk*NORday** | HFD_Low | 1 | SD_High | 3 | -66.5441 | 9.5824 | 44 | -6.94 | <.0001 |
| **gk*NORday** | HFD_Low | 1 | SD_High | 7 | -19.4908 | 5.4139 | 44 | -3.60 | 0.0008 |
| **gk*NORday** | HFD_Low | 1 | SD_Low | 0 | -15.9035 | 8.1059 | 44 | -1.96 | 0.0561 |
| **gk*NORday** | HFD_Low | 1 | SD_Low | 1 | -9.0035 | 5.8649 | 44 | -1.54 | 0.1319 |
| **gk*NORday** | HFD_Low | 1 | SD_Low | 3 | -16.5217 | 10.9307 | 44 | -1.51 | 0.1378 |
| **gk*NORday** | HFD_Low | 1 | SD_Low | 7 | -12.4399 | 5.8511 | 44 | -2.13 | 0.0391 |
| **gk*NORday** | HFD_Low | 3 | HFD_Low | 7 | 15.7538 | 8.3453 | 44 | 1.89 | 0.0657 |
| **gk*NORday** | HFD_Low | 3 | SD_High | 0 | 8.8700 | 11.2548 | 44 | 0.79 | 0.4349 |
| **gk*NORday** | HFD_Low | 3 | SD_High | 1 | 2.0128 | 10.0709 | 44 | 0.20 | 0.8425 |
| **gk*NORday** | HFD_Low | 3 | SD_High | 3 | -44.5672 | 12.7991 | 44 | -3.48 | 0.0011 |
| **gk*NORday** | HFD_Low | 3 | SD_High | 7 | 2.4862 | 10.0650 | 44 | 0.25 | 0.8060 |
| **gk*NORday** | HFD_Low | 3 | SD_Low | 0 | 6.0734 | 11.7345 | 44 | 0.52 | 0.6074 |
| **gk*NORday** | HFD_Low | 3 | SD_Low | 1 | 12.9734 | 10.3146 | 44 | 1.26 | 0.2151 |
| **gk*NORday** | HFD_Low | 3 | SD_Low | 3 | 5.4552 | 13.8374 | 44 | 0.39 | 0.6953 |
| **gk*NORday** | HFD_Low | 3 | SD_Low | 7 | 9.5371 | 10.3068 | 44 | 0.93 | 0.3598 |
| **gk*NORday** | HFD_Low | 7 | SD_High | 0 | -6.8839 | 7.3851 | 44 | -0.93 | 0.3564 |
| **gk*NORday** | HFD_Low | 7 | SD_High | 1 | -13.7410 | 5.4122 | 44 | -2.54 | 0.0147 |
| **gk*NORday** | HFD_Low | 7 | SD_High | 3 | -60.3210 | 9.5753 | 44 | -6.30 | <.0001 |
| **gk*NORday** | HFD_Low | 7 | SD_High | 7 | -13.2677 | 5.4013 | 44 | -2.46 | 0.0181 |
| **gk*NORday** | HFD_Low | 7 | SD_Low | 0 | -9.6804 | 8.0975 | 44 | -1.20 | 0.2383 |
| **gk*NORday** | HFD_Low | 7 | SD_Low | 1 | -2.7804 | 5.8532 | 44 | -0.48 | 0.6371 |
| **gk*NORday** | HFD_Low | 7 | SD_Low | 3 | -10.2986 | 10.9245 | 44 | -0.94 | 0.3510 |
| **gk*NORday** | HFD_Low | 7 | SD_Low | 7 | -6.2168 | 5.8395 | 44 | -1.06 | 0.2929 |
| **gk*NORday** | SD_High | 0 | SD_High | 1 | -6.8571 | 6.8857 | 44 | -1.00 | 0.3248 |
| **gk*NORday** | SD_High | 0 | SD_High | 3 | -53.4371 | 9.0876 | 44 | -5.88 | <.0001 |
| **gk*NORday** | SD_High | 0 | SD_High | 7 | -6.3838 | 6.2716 | 44 | -1.02 | 0.3143 |
| **gk*NORday** | SD_High | 0 | SD_Low | 0 | -2.7965 | 9.4261 | 44 | -0.30 | 0.7681 |
| **gk*NORday** | SD_High | 0 | SD_Low | 1 | 4.1035 | 7.5857 | 44 | 0.54 | 0.5913 |
| **gk*NORday** | SD_High | 0 | SD_Low | 3 | -3.4147 | 11.9426 | 44 | -0.29 | 0.7763 |
| **gk*NORday** | SD_High | 0 | SD_Low | 7 | 0.6671 | 7.5751 | 44 | 0.09 | 0.9302 |
| **gk*NORday** | SD_High | 1 | SD_High | 3 | -46.5800 | 8.9835 | 44 | -5.19 | <.0001 |
| **gk*NORday** | SD_High | 1 | SD_High | 7 | 0.4733 | 4.3987 | 44 | 0.11 | 0.9148 |
| **gk*NORday** | SD_High | 1 | SD_Low | 0 | 4.0606 | 7.9752 | 44 | 0.51 | 0.6132 |
| **gk*NORday** | SD_High | 1 | SD_Low | 1 | 10.9606 | 5.6828 | 44 | 1.93 | 0.0602 |
| **gk*NORday** | SD_High | 1 | SD_Low | 3 | 3.4424 | 10.8341 | 44 | 0.32 | 0.7522 |
| **gk*NORday** | SD_High | 1 | SD_Low | 7 | 7.5242 | 5.6686 | 44 | 1.33 | 0.1912 |
| **gk*NORday** | SD_High | 3 | SD_High | 7 | 47.0533 | 7.7691 | 44 | 6.06 | <.0001 |
| **gk*NORday** | SD_High | 3 | SD_Low | 0 | 50.6406 | 11.2249 | 44 | 4.51 | <.0001 |
| **gk*NORday** | SD_High | 3 | SD_Low | 1 | 57.5406 | 9.7308 | 44 | 5.91 | <.0001 |
| **gk*NORday** | SD_High | 3 | SD_Low | 3 | 50.0224 | 13.4079 | 44 | 3.73 | 0.0005 |
| **gk*NORday** | SD_High | 3 | SD_Low | 7 | 54.1042 | 9.7226 | 44 | 5.56 | <.0001 |
| **gk*NORday** | SD_High | 7 | SD_Low | 0 | 3.5873 | 7.9678 | 44 | 0.45 | 0.6548 |
| **gk*NORday** | SD_High | 7 | SD_Low | 1 | 10.4873 | 5.6724 | 44 | 1.85 | 0.0712 |
| **gk*NORday** | SD_High | 7 | SD_Low | 3 | 2.9691 | 10.8287 | 44 | 0.27 | 0.7852 |
| **gk*NORday** | SD_High | 7 | SD_Low | 7 | 7.0509 | 5.6582 | 44 | 1.25 | 0.2193 |
| **gk*NORday** | SD_Low | 0 | SD_Low | 1 | 6.9000 | 7.8443 | 44 | 0.88 | 0.3838 |
| **gk*NORday** | SD_Low | 0 | SD_Low | 3 | -0.6182 | 10.4640 | 44 | -0.06 | 0.9532 |
| **gk*NORday** | SD_Low | 0 | SD_Low | 7 | 3.4636 | 7.1074 | 44 | 0.49 | 0.6284 |
| **gk*NORday** | SD_Low | 1 | SD_Low | 3 | -7.5182 | 10.4905 | 44 | -0.72 | 0.4774 |
| **gk*NORday** | SD_Low | 1 | SD_Low | 7 | -3.4364 | 5.1366 | 44 | -0.67 | 0.5070 |
| **gk*NORday** | SD_Low | 3 | SD_Low | 7 | 4.0818 | 9.0723 | 44 | 0.45 | 0.6550 |

| **Model Information** | |
| --- | --- |
| **Data Set** | WORK.NOR2 |
| **Dependent Variable** | transratio |
| **Covariance Structure** | Unstructured |
| **Subject Effect** | ID(gk) |
| **Estimation Method** | REML |
| **Residual Variance Method** | None |
| **Fixed Effects SE Method** | Model-Based |
| **Degrees of Freedom Method** | Between-Within |

| **Class Level Information** | | |
| --- | --- | --- |
| **Class** | **Levels** | **Values** |
| **gk** | 4 | HFD_High HFD_Low SD_High SD_Low |
| **ID** | 48 | C1 C10 C11 C12 C13 C14 C15 C16 C17 C18 C19 C2 C20 C21 C22 C23 C24 C25 C26 C3 C4 C5 C6 C7 C8 C9 T1 T10 T11 T12 T13 T14 T15 T17 T2 T21 T22 T23 T24 T26 T27 T3 T4 T5 T6 T7 T8 T9 |
| **NORday** | 4 | 0 1 3 7 |

| **Dimensions** | |
| --- | --- |
| **Covariance Parameters** | 10 |
| **Columns in X** | 25 |
| **Columns in Z** | 0 |
| **Subjects** | 48 |
| **Max Obs per Subject** | 4 |

| **Number of Observations** | |
| --- | --- |
| **Number of Observations Read** | 192 |
| **Number of Observations Used** | 191 |
| **Number of Observations Not Used** | 1 |

| **Iteration History** | | | |
| --- | --- | --- | --- |
| **Iteration** | **Evaluations** | **-2 Res Log Like** | **Criterion** |
| **0** | 1 | 394.75005116 |  |
| **1** | 2 | 352.72194150 | 0.00001500 |
| **2** | 1 | 352.72170744 | 0.00000000 |

| Convergence criteria met. |
| --- |

| **Covariance Parameter Estimates** | | |
| --- | --- | --- |
| **Cov Parm** | **Subject** | **Estimate** |
| **UN(1,1)** | ID(gk) | 0.9132 |
| **UN(2,1)** | ID(gk) | -0.03532 |
| **UN(2,2)** | ID(gk) | 0.2057 |
| **UN(3,1)** | ID(gk) | 0.07661 |
| **UN(3,2)** | ID(gk) | -0.01436 |
| **UN(3,3)** | ID(gk) | 0.3492 |
| **UN(4,1)** | ID(gk) | 0.1676 |
| **UN(4,2)** | ID(gk) | 0.04474 |
| **UN(4,3)** | ID(gk) | 0.1389 |
| **UN(4,4)** | ID(gk) | 0.3330 |

| **Fit Statistics** | |
| --- | --- |
| **-2 Res Log Likelihood** | 352.7 |
| **AIC (Smaller is Better)** | 372.7 |
| **AICC (Smaller is Better)** | 374.1 |
| **BIC (Smaller is Better)** | 391.4 |

| **Null Model Likelihood Ratio Test** | | |
| --- | --- | --- |
| **DF** | **Chi-Square** | **Pr > ChiSq** |
| 9 | 42.03 | <.0001 |

| **Type 3 Tests of Fixed Effects** | | | | |
| --- | --- | --- | --- | --- |
| **Effect** | **Num DF** | **Den DF** | **F Value** | **Pr > F** |
| **gk** | 3 | 44 | 2.22 | 0.0986 |
| **NORday** | 3 | 44 | 19.23 | <.0001 |
| **gk*NORday** | 9 | 44 | 2.81 | 0.0106 |

| **Least Squares Means** | | | | | | |
| --- | --- | --- | --- | --- | --- | --- |
| **Effect** | **gk** | **Estimate** | **Standard Error** | **DF** | **t Value** | **Pr > |t|** |
| **gk** | HFD_High | 0.2872 | 0.1333 | 44 | 2.15 | 0.0367 |
| **gk** | HFD_Low | 0.2604 | 0.1109 | 44 | 2.35 | 0.0234 |
| **gk** | SD_High | 0.6025 | 0.1044 | 44 | 5.77 | <.0001 |
| **gk** | SD_Low | 0.2961 | 0.1205 | 44 | 2.46 | 0.0181 |

| **Differences of Least Squares Means** | | | | | | | |
| --- | --- | --- | --- | --- | --- | --- | --- |
| **Effect** | **gk** | **_gk** | **Estimate** | **Standard Error** | **DF** | **t Value** | **Pr > |t|** |
| **gk** | HFD_High | HFD_Low | 0.02674 | 0.1734 | 44 | 0.15 | 0.8781 |
| **gk** | HFD_High | SD_High | -0.3153 | 0.1693 | 44 | -1.86 | 0.0692 |
| **gk** | HFD_High | SD_Low | -0.00891 | 0.1797 | 44 | -0.05 | 0.9607 |
| **gk** | HFD_Low | SD_High | -0.3421 | 0.1523 | 44 | -2.25 | 0.0298 |
| **gk** | HFD_Low | SD_Low | -0.03565 | 0.1638 | 44 | -0.22 | 0.8287 |
| **gk** | SD_High | SD_Low | 0.3064 | 0.1595 | 44 | 1.92 | 0.0612 |

| **Obs** | **ID** | **Group** | **noresponse** | **Day** | **Week** | **wt** | **glucose** | **ketone** | **distance** | **distDay** | **timeA** | **timeX** |
| --- | --- | --- | --- | --- | --- | --- | --- | --- | --- | --- | --- | --- |
| **1** | C1 | SD | 0 | 21 | 3 | 305.40 | . | . | . | . | 61.0 | 44.9 |
| **2** | C1 | SD | 0 | 22 | 4 | 308.25 | 97 | 1.1 | . | . | 74.2 | 57.0 |
| **3** | C1 | SD | 0 | 24 | 4 | 322.00 | . | . | . | . | 22.5 | 178.4 |
| **4** | C1 | SD | 0 | 28 | 4 | 329.03 | . | . | . | . | 60.2 | 62.7 |
| **5** | C10 | SD | 0 | 31 | 5 | . | 103 | 0.6 | . | . | 40.4 | 42.7 |
| **6** | C10 | SD | 0 | 32 | 5 | . | . | . | . | . | 20.3 | 51.5 |
| **7** | C10 | SD | 0 | 34 | 5 | 359.00 | . | . | . | . | 27.7 | 61.5 |
| **8** | C10 | SD | 0 | 38 | 6 | 373.00 | 94 | 0.1 | . | . | 22.9 | 45.9 |
| **9** | C11 | SD | 0 | 31 | 5 | . | 96 | 0.1 | . | . | 29.6 | 43.6 |
| **10** | C11 | SD | 0 | 32 | 5 | . | . | . | . | . | 29.4 | 30.0 |
| **11** | C11 | SD | 0 | 34 | 5 | 337.00 | . | . | . | . | 19.9 | 41.2 |
| **12** | C11 | SD | 0 | 38 | 6 | 344.00 | 108 | 0.1 | . | . | 39.1 | 70.0 |
| **13** | C12 | SD | 0 | 31 | 5 | . | 100 | 0.1 | . | . | 19.1 | 35.0 |
| **14** | C12 | SD | 0 | 32 | 5 | . | . | . | . | . | 35.2 | 21.6 |
| **15** | C12 | SD | 0 | 34 | 5 | 340.00 | . | . | . | . | 27.5 | 35.0 |
| **16** | C12 | SD | 0 | 38 | 6 | 348.00 | 93 | 0.1 | . | . | 36.2 | 28.2 |
| **17** | C13 | SD | 0 | 31 | 5 | . | . | . | . | . | 48.5 | 16.3 |
| **18** | C13 | SD | 0 | 32 | 5 | . | . | . | . | . | 18.4 | 57.9 |
| **19** | C13 | SD | 0 | 34 | 5 | . | . | . | . | . | 45.7 | 58.5 |
| **20** | C13 | SD | 0 | 38 | 6 | . | . | . | . | . | 19.2 | 54.2 |
| **21** | C14 | SD | 0 | 31 | 5 | . | . | . | . | . | 4.9 | 28.3 |
| **22** | C14 | SD | 0 | 32 | 5 | . | . | . | . | . | 40.7 | 73.3 |
| **23** | C14 | SD | 0 | 34 | 5 | . | . | . | . | . | 26.7 | 78.6 |
| **24** | C14 | SD | 0 | 38 | 6 | . | . | . | . | . | 20.4 | 78.5 |
| **25** | C15 | SD | 0 | 31 | 5 | . | . | . | . | . | 31.0 | 30.0 |
| **26** | C15 | SD | 0 | 32 | 5 | . | . | . | . | . | 31.6 | 37.1 |
| **27** | C15 | SD | 0 | 34 | 5 | . | . | . | . | . | 38.0 | 94.5 |
| **28** | C15 | SD | 0 | 38 | 6 | . | . | . | . | . | 38.5 | 67.9 |
| **29** | C16 | SD | 0 | 31 | 5 | . | . | . | . | . | 24.5 | 58.3 |
| **30** | C16 | SD | 0 | 32 | 5 | . | . | . | . | . | 27.6 | 51.9 |
| **31** | C16 | SD | 0 | 34 | 5 | . | . | . | . | . | 25.7 | 123.2 |
| **32** | C16 | SD | 0 | 38 | 6 | . | . | . | . | . | 17.7 | 42.6 |
| **33** | C17 | SD | 0 | 31 | 5 | . | . | . | . | . | 13.1 | 33.0 |
| **34** | C17 | SD | 0 | 32 | 5 | . | . | . | . | . | 27.0 | 65.9 |
| **35** | C17 | SD | 0 | 34 | 5 | . | . | . | . | . | 31.7 | 97.4 |
| **36** | C17 | SD | 0 | 38 | 6 | . | . | . | . | . | 42.2 | 55.6 |
| **37** | C18 | SD | 0 | 31 | 5 | . | . | . | . | . | 45.5 | 27.4 |
| **38** | C18 | SD | 0 | 32 | 5 | . | . | . | . | . | 28.7 | 49.7 |
| **39** | C18 | SD | 0 | 34 | 5 | . | . | . | . | . | 31.2 | 109.5 |
| **40** | C18 | SD | 0 | 38 | 6 | . | . | . | . | . | 31.9 | 57.5 |
| **41** | C19 | SD | 0 | 31 | 5 | . | . | . | . | . | 34.7 | 26.6 |
| **42** | C19 | SD | 0 | 32 | 5 | . | . | . | . | . | 27.4 | 28.2 |
| **43** | C19 | SD | 0 | 34 | 5 | . | . | . | . | . | 40.9 | 67.8 |
| **44** | C19 | SD | 0 | 38 | 6 | . | . | . | . | . | 45.9 | 43.9 |
| **45** | C2 | SD | 0 | 21 | 3 | 310.03 | . | . | . | . | 14.1 | . |
| **46** | C2 | SD | 0 | 22 | 4 | 310.17 | 80 | 0.8 | . | . | 22.6 | 45.7 |
| **47** | C2 | SD | 0 | 24 | 4 | 320.00 | . | . | . | . | 24.0 | 115.8 |
| **48** | C2 | SD | 0 | 28 | 4 | 325.23 | . | . | . | . | 71.8 | 43.0 |
| **49** | C20 | SD | 0 | 31 | 5 | . | . | . | . | . | 28.4 | 32.5 |
| **50** | C20 | SD | 0 | 32 | 5 | . | . | . | . | . | 13.4 | 62.4 |

| **Obs** | **NORday** | **calintake** | **wk_wt** | **wk_glucose** | **wk_ketone** | **wk_distance** | **wk_timeA** | **wk_timeX** |
| --- | --- | --- | --- | --- | --- | --- | --- | --- |
| **1** | 0 | . | 303.831 | 106.0 | 0.95 | 4.31212 | 61.0000 | 44.9000 |
| **2** | 1 | . | 318.493 | 95.5 | 1.10 | . | 52.3000 | 99.3667 |
| **3** | 3 | . | 318.493 | 95.5 | 1.10 | . | 52.3000 | 99.3667 |
| **4** | 7 | . | 318.493 | 95.5 | 1.10 | . | 52.3000 | 99.3667 |
| **5** | 0 | . | 359.000 | 97.5 | 0.35 | 3.79365 | 29.4667 | 51.9000 |
| **6** | 1 | . | 359.000 | 97.5 | 0.35 | 3.79365 | 29.4667 | 51.9000 |
| **7** | 3 | 57.97 | 359.000 | 97.5 | 0.35 | 3.79365 | 29.4667 | 51.9000 |
| **8** | 7 | . | 373.000 | 94.0 | 0.10 | . | 22.9000 | 45.9000 |
| **9** | 0 | . | 337.000 | 95.0 | 0.10 | 3.97005 | 26.3000 | 38.2667 |
| **10** | 1 | . | 337.000 | 95.0 | 0.10 | 3.97005 | 26.3000 | 38.2667 |
| **11** | 3 | 37.51 | 337.000 | 95.0 | 0.10 | 3.97005 | 26.3000 | 38.2667 |
| **12** | 7 | . | 344.000 | 108.0 | 0.10 | . | 39.1000 | 70.0000 |
| **13** | 0 | . | 340.000 | 93.5 | 0.45 | 3.45975 | 27.2667 | 30.5333 |
| **14** | 1 | . | 340.000 | 93.5 | 0.45 | 3.45975 | 27.2667 | 30.5333 |
| **15** | 3 | 40.92 | 340.000 | 93.5 | 0.45 | 3.45975 | 27.2667 | 30.5333 |
| **16** | 7 | . | 348.000 | 93.0 | 0.10 | . | 36.2000 | 28.2000 |
| **17** | 0 | . | . | 90.0 | 1.10 | . | 37.5333 | 44.2333 |
| **18** | 1 | . | . | 90.0 | 1.10 | . | 37.5333 | 44.2333 |
| **19** | 3 | . | . | 90.0 | 1.10 | . | 37.5333 | 44.2333 |
| **20** | 7 | . | 368.000 | . | . | . | 19.2000 | 54.2000 |
| **21** | 0 | . | . | 100.0 | 1.60 | . | 24.1000 | 60.0667 |
| **22** | 1 | . | . | 100.0 | 1.60 | . | 24.1000 | 60.0667 |
| **23** | 3 | . | . | 100.0 | 1.60 | . | 24.1000 | 60.0667 |
| **24** | 7 | . | 355.000 | . | . | . | 20.4000 | 78.5000 |
| **25** | 0 | . | . | 95.0 | 1.30 | . | 33.5333 | 53.8667 |
| **26** | 1 | . | . | 95.0 | 1.30 | . | 33.5333 | 53.8667 |
| **27** | 3 | . | . | 95.0 | 1.30 | . | 33.5333 | 53.8667 |
| **28** | 7 | . | 374.000 | . | . | . | 38.5000 | 67.9000 |
| **29** | 0 | . | . | 81.0 | 1.10 | . | 25.9333 | 77.8000 |
| **30** | 1 | . | . | 81.0 | 1.10 | . | 25.9333 | 77.8000 |
| **31** | 3 | . | . | 81.0 | 1.10 | . | 25.9333 | 77.8000 |
| **32** | 7 | . | 368.000 | . | . | . | 17.7000 | 42.6000 |
| **33** | 0 | . | . | 89.0 | 1.40 | . | 23.9333 | 65.4333 |
| **34** | 1 | . | . | 89.0 | 1.40 | . | 23.9333 | 65.4333 |
| **35** | 3 | . | . | 89.0 | 1.40 | . | 23.9333 | 65.4333 |
| **36** | 7 | . | 395.000 | . | . | . | 42.2000 | 55.6000 |
| **37** | 0 | . | . | 95.0 | 1.00 | . | 35.1333 | 62.2000 |
| **38** | 1 | . | . | 95.0 | 1.00 | . | 35.1333 | 62.2000 |
| **39** | 3 | . | . | 95.0 | 1.00 | . | 35.1333 | 62.2000 |
| **40** | 7 | . | 366.000 | . | . | . | 31.9000 | 57.5000 |
| **41** | 0 | . | . | 87.0 | 1.10 | . | 34.3333 | 40.8667 |
| **42** | 1 | . | . | 87.0 | 1.10 | . | 34.3333 | 40.8667 |
| **43** | 3 | . | . | 87.0 | 1.10 | . | 34.3333 | 40.8667 |
| **44** | 7 | . | 396.000 | . | . | . | 45.9000 | 43.9000 |
| **45** | 0 | . | 302.083 | 106.0 | 1.25 | 5.27552 | 14.1000 | . |
| **46** | 1 | . | 316.848 | 87.0 | 0.90 | . | 39.4667 | 68.1667 |
| **47** | 3 | . | 316.848 | 87.0 | 0.90 | . | 39.4667 | 68.1667 |
| **48** | 7 | . | 316.848 | 87.0 | 0.90 | . | 39.4667 | 68.1667 |
| **49** | 0 | . | . | 87.0 | 0.90 | . | 20.8000 | 47.2333 |
| **50** | 1 | . | . | 87.0 | 0.90 | . | 20.8000 | 47.2333 |

| **Obs** | **wk_calintake** | **ratioxa** | **transratio** | **transa** | **transx** |
| --- | --- | --- | --- | --- | --- |
| **1** | . | 0.73607 | -0.30644 | 4.11087 | 3.80444 |
| **2** | . | 0.76819 | -0.26371 | 4.30676 | 4.04305 |
| **3** | . | 7.92889 | 2.07051 | 3.11352 | 5.18403 |
| **4** | . | 1.04153 | 0.04069 | 4.09767 | 4.13836 |
| **5** | 57.97 | 1.05693 | 0.05537 | 3.69883 | 3.75420 |
| **6** | 57.97 | 2.53695 | 0.93096 | 3.01062 | 3.94158 |
| **7** | 57.97 | 2.22022 | 0.79760 | 3.32143 | 4.11904 |
| **8** | . | 2.00437 | 0.69533 | 3.13114 | 3.82647 |
| **9** | 37.51 | 1.47297 | 0.38728 | 3.38777 | 3.77506 |
| **10** | 37.51 | 1.02041 | 0.02020 | 3.38099 | 3.40120 |
| **11** | 37.51 | 2.07035 | 0.72772 | 2.99072 | 3.71844 |
| **12** | . | 1.79028 | 0.58237 | 3.66612 | 4.24850 |
| **13** | 40.92 | 1.83246 | 0.60566 | 2.94969 | 3.55535 |
| **14** | 40.92 | 0.61364 | -0.48835 | 3.56105 | 3.07269 |
| **15** | 40.92 | 1.27273 | 0.24116 | 3.31419 | 3.55535 |
| **16** | . | 0.77901 | -0.24974 | 3.58906 | 3.33932 |
| **17** | . | 0.33608 | -1.09040 | 3.88156 | 2.79117 |
| **18** | . | 3.14674 | 1.14637 | 2.91235 | 4.05872 |
| **19** | . | 1.28009 | 0.24693 | 3.82210 | 4.06903 |
| **20** | . | 2.82292 | 1.03777 | 2.95491 | 3.99268 |
| **21** | . | 5.77551 | 1.75363 | 1.58924 | 3.34286 |
| **22** | . | 1.80098 | 0.58833 | 3.70623 | 4.29456 |
| **23** | . | 2.94382 | 1.07971 | 3.28466 | 4.36437 |
| **24** | . | 3.84804 | 1.34756 | 3.01553 | 4.36310 |
| **25** | . | 0.96774 | -0.03279 | 3.43399 | 3.40120 |
| **26** | . | 1.17405 | 0.16046 | 3.45316 | 3.61362 |
| **27** | . | 2.48684 | 0.91101 | 3.63759 | 4.54860 |
| **28** | . | 1.76364 | 0.56738 | 3.65066 | 4.21804 |
| **29** | . | 2.37959 | 0.86693 | 3.19867 | 4.06560 |
| **30** | . | 1.88043 | 0.63150 | 3.31782 | 3.94932 |
| **31** | . | 4.79377 | 1.56732 | 3.24649 | 4.81381 |
| **32** | . | 2.40678 | 0.87829 | 2.87356 | 3.75185 |
| **33** | . | 2.51908 | 0.92390 | 2.57261 | 3.49651 |
| **34** | . | 2.44074 | 0.89230 | 3.29584 | 4.18814 |
| **35** | . | 3.07256 | 1.12251 | 3.45632 | 4.57883 |
| **36** | . | 1.31754 | 0.27576 | 3.74242 | 4.01818 |
| **37** | . | 0.60220 | -0.50717 | 3.81771 | 3.31054 |
| **38** | . | 1.73171 | 0.54911 | 3.35690 | 3.90600 |
| **39** | . | 3.50962 | 1.25551 | 3.44042 | 4.69592 |
| **40** | . | 1.80251 | 0.58918 | 3.46261 | 4.05178 |
| **41** | . | 0.76657 | -0.26583 | 3.54674 | 3.28091 |
| **42** | . | 1.02920 | 0.02878 | 3.31054 | 3.33932 |
| **43** | . | 1.65770 | 0.50543 | 3.71113 | 4.21656 |
| **44** | . | 0.95643 | -0.04455 | 3.82647 | 3.78191 |
| **45** | . | . | . | 2.64617 | . |
| **46** | . | 2.02212 | 0.70415 | 3.11795 | 3.82210 |
| **47** | . | 4.82500 | 1.57381 | 3.17805 | 4.75186 |
| **48** | . | 0.59889 | -0.51268 | 4.27388 | 3.76120 |
| **49** | . | 1.14437 | 0.13485 | 3.34639 | 3.48124 |
| **50** | . | 4.65672 | 1.53831 | 2.59525 | 4.13357 |

| **Model Information** | |
| --- | --- |
| **Data Set** | WORK.NOR |
| **Dependent Variable** | timeX |
| **Covariance Structure** | Unstructured |
| **Subject Effect** | ID(Group) |
| **Estimation Method** | REML |
| **Residual Variance Method** | None |
| **Fixed Effects SE Method** | Model-Based |
| **Degrees of Freedom Method** | Between-Within |

| **Class Level Information** | | |
| --- | --- | --- |
| **Class** | **Levels** | **Values** |
| **Group** | 2 | HFD SD |
| **ID** | 48 | C1 C10 C11 C12 C13 C14 C15 C16 C17 C18 C19 C2 C20 C21 C22 C23 C24 C25 C26 C3 C4 C5 C6 C7 C8 C9 T1 T10 T11 T12 T13 T14 T15 T17 T2 T21 T22 T23 T24 T26 T27 T3 T4 T5 T6 T7 T8 T9 |
| **NORday** | 4 | 0 1 3 7 |

| **Dimensions** | |
| --- | --- |
| **Covariance Parameters** | 10 |
| **Columns in X** | 8 |
| **Columns in Z** | 0 |
| **Subjects** | 48 |
| **Max Obs per Subject** | 4 |

| **Number of Observations** | |
| --- | --- |
| **Number of Observations Read** | 192 |
| **Number of Observations Used** | 175 |
| **Number of Observations Not Used** | 17 |

| **Iteration History** | | | |
| --- | --- | --- | --- |
| **Iteration** | **Evaluations** | **-2 Res Log Like** | **Criterion** |
| **0** | 1 | 1570.33721677 |  |
| **1** | 2 | 1505.23630359 | 0.00159143 |
| **2** | 1 | 1504.12157903 | 0.00019155 |
| **3** | 1 | 1503.99598313 | 0.00000572 |
| **4** | 1 | 1503.99249621 | 0.00000001 |

| Convergence criteria met. |
| --- |

| **Covariance Parameter Estimates** | | |
| --- | --- | --- |
| **Cov Parm** | **Subject** | **Estimate** |
| **UN(1,1)** | ID(Group) | 580.58 |
| **UN(2,1)** | ID(Group) | 57.9401 |
| **UN(2,2)** | ID(Group) | 171.03 |
| **UN(3,1)** | ID(Group) | 55.6971 |
| **UN(3,2)** | ID(Group) | -101.98 |
| **UN(3,3)** | ID(Group) | 1175.56 |
| **UN(4,1)** | ID(Group) | 186.95 |
| **UN(4,2)** | ID(Group) | -3.9113 |
| **UN(4,3)** | ID(Group) | 116.04 |
| **UN(4,4)** | ID(Group) | 200.71 |

| **Fit Statistics** | |
| --- | --- |
| **-2 Res Log Likelihood** | 1504.0 |
| **AIC (Smaller is Better)** | 1524.0 |
| **AICC (Smaller is Better)** | 1525.4 |
| **BIC (Smaller is Better)** | 1542.7 |

| **Null Model Likelihood Ratio Test** | | |
| --- | --- | --- |
| **DF** | **Chi-Square** | **Pr > ChiSq** |
| 9 | 66.34 | <.0001 |

| **Type 3 Tests of Fixed Effects** | | | | |
| --- | --- | --- | --- | --- |
| **Effect** | **Num DF** | **Den DF** | **F Value** | **Pr > F** |
| **Group** | 1 | 46 | 0.04 | 0.8509 |
| **NORday** | 3 | 46 | 12.72 | <.0001 |
| **wk_ketone** | 1 | 46 | 80.59 | <.0001 |

| **Least Squares Means** | | | | | | |
| --- | --- | --- | --- | --- | --- | --- |
| **Effect** | **NORday** | **Estimate** | **Standard Error** | **DF** | **t Value** | **Pr > |t|** |
| **NORday** | 0 | 45.9815 | 3.5047 | 46 | 13.12 | <.0001 |
| **NORday** | 1 | 45.1499 | 1.8950 | 46 | 23.83 | <.0001 |
| **NORday** | 3 | 79.2187 | 4.9516 | 46 | 16.00 | <.0001 |
| **NORday** | 7 | 52.5999 | 2.3769 | 46 | 22.13 | <.0001 |

| **Differences of Least Squares Means** | | | | | | | |
| --- | --- | --- | --- | --- | --- | --- | --- |
| **Effect** | **NORday** | **_NORday** | **Estimate** | **Standard Error** | **DF** | **t Value** | **Pr > |t|** |
| **NORday** | 0 | 1 | 0.8315 | 3.6643 | 46 | 0.23 | 0.8215 |
| **NORday** | 0 | 3 | -33.2372 | 5.8693 | 46 | -5.66 | <.0001 |
| **NORday** | 0 | 7 | -6.6184 | 3.2027 | 46 | -2.07 | 0.0444 |
| **NORday** | 1 | 3 | -34.0687 | 5.6836 | 46 | -5.99 | <.0001 |
| **NORday** | 1 | 7 | -7.4499 | 3.0835 | 46 | -2.42 | 0.0197 |
| **NORday** | 3 | 7 | 26.6188 | 5.0436 | 46 | 5.28 | <.0001 |

| **Moments** | | | |
| --- | --- | --- | --- |
| **N** | 175 | **Sum Weights** | 175 |
| **Mean** | 0.13646935 | **Sum Observations** | 23.8821364 |
| **Std Deviation** | 23.3659535 | **Variance** | 545.967784 |
| **Skewness** | 0.8796606 | **Kurtosis** | 2.02791648 |
| **Uncorrected SS** | 95001.6537 | **Corrected SS** | 94998.3945 |
| **Coeff Variation** | 17121.7591 | **Std Error Mean** | 1.76630006 |

| **Basic Statistical Measures** | | | |
| --- | --- | --- | --- |
| **Location** | | **Variability** | |
| **Mean** | 0.136469 | **Std Deviation** | 23.36595 |
| **Median** | 0.234901 | **Variance** | 545.96778 |
| **Mode** | . | **Range** | 137.73478 |
|  |  | **Interquartile Range** | 24.16875 |

| **Tests for Location: Mu0=0** | | | | |
| --- | --- | --- | --- | --- |
| **Test** | **Statistic** | | **p Value** | |
| **Student's t** | **t** | 0.077263 | **Pr > |t|** | 0.9385 |
| **Sign** | **M** | 0.5 | **Pr >= |M|** | 1.0000 |
| **Signed Rank** | **S** | -376 | **Pr >= |S|** | 0.5768 |

| **Tests for Normality** | | | | |
| --- | --- | --- | --- | --- |
| **Test** | **Statistic** | | **p Value** | |
| **Shapiro-Wilk** | **W** | 0.94712 | **Pr < W** | <0.0001 |
| **Kolmogorov-Smirnov** | **D** | 0.095297 | **Pr > D** | <0.0100 |
| **Cramer-von Mises** | **W-Sq** | 0.330633 | **Pr > W-Sq** | <0.0050 |
| **Anderson-Darling** | **A-Sq** | 1.98763 | **Pr > A-Sq** | <0.0050 |

| **Quantiles (Definition 5)** | |
| --- | --- |
| **Level** | **Quantile** |
| **100% Max** | 89.752924 |
| **99%** | 81.209792 |
| **95%** | 36.536536 |
| **90%** | 29.795318 |
| **75% Q3** | 10.399475 |
| **50% Median** | 0.234901 |
| **25% Q1** | -13.769275 |
| **10%** | -29.955021 |
| **5%** | -36.573432 |
| **1%** | -41.013514 |
| **0% Min** | -47.981853 |

| **Extreme Observations** | | | |
| --- | --- | --- | --- |
| **Lowest** | | **Highest** | |
| **Value** | **Obs** | **Value** | **Obs** |
| -47.9819 | 185 | 58.4584 | 97 |
| -41.0135 | 106 | 71.6809 | 179 |
| -40.9140 | 131 | 76.9177 | 107 |
| -40.2940 | 21 | 81.2098 | 139 |
| -39.3140 | 155 | 89.7529 | 3 |

| **Missing Values** | | | |
| --- | --- | --- | --- |
| **Missing Value** | **Count** | **Percent Of** | |
|  |  | **All Obs** | **Missing Obs** |
| . | 17 | 8.85 | 100.00 |

**Group=HFD Day=31**

| **Variable** | **N** | **Mean** | **Std Dev** | **Minimum** | **Maximum** |
| --- | --- | --- | --- | --- | --- |
| timeA timeX ratioxa | 12 12 12 | 19.5916667 44.5416667 3.9708781 | 15.1268851 17.4236234 3.5270393 | 2.6000000 22.3000000 0.7644991 | 56.9000000 80.7000000 10.7600000 |

**Group=HFD Day=32**

| **Variable** | **N** | **Mean** | **Std Dev** | **Minimum** | **Maximum** |
| --- | --- | --- | --- | --- | --- |
| timeA timeX ratioxa | 12 12 12 | 42.0833333 35.0583333 0.9462860 | 20.2876063 7.8660904 0.3274633 | 21.8000000 21.3000000 0.5814815 | 81.0000000 47.1000000 1.4262948 |

**Group=HFD Day=34**

| **Variable** | **N** | **Mean** | **Std Dev** | **Minimum** | **Maximum** |
| --- | --- | --- | --- | --- | --- |
| timeA timeX ratioxa | 12 12 12 | 32.5666667 45.7666667 1.6861984 | 11.0900720 29.2104693 1.4961663 | 20.8000000 21.9000000 0.5488722 | 52.0000000 114.7000000 5.0087336 |

**Group=HFD Day=38**

| **Variable** | **N** | **Mean** | **Std Dev** | **Minimum** | **Maximum** |
| --- | --- | --- | --- | --- | --- |
| timeA timeX ratioxa | 12 12 12 | 42.0333333 40.9500000 1.2405434 | 22.0290855 10.3140240 0.6787224 | 17.2000000 26.0000000 0.4311774 | 88.7000000 64.2000000 2.3627451 |

**Group=SD Day=31**

| **Variable** | **N** | **Mean** | **Std Dev** | **Minimum** | **Maximum** |
| --- | --- | --- | --- | --- | --- |
| timeA timeX ratioxa | 20 20 20 | 26.1550000 49.8800000 2.9486163 | 13.1088751 27.7228502 3.2490853 | 4.9000000 16.3000000 0.3360825 | 48.5000000 105.7000000 14.4794521 |

**Group=SD Day=32**

| **Variable** | **N** | **Mean** | **Std Dev** | **Minimum** | **Maximum** |
| --- | --- | --- | --- | --- | --- |
| timeA timeX ratioxa | 20 20 20 | 34.9300000 49.3950000 1.6193622 | 12.1485585 17.6054260 0.9755519 | 13.4000000 21.6000000 0.6136364 | 62.6000000 79.1000000 4.6567164 |

**Group=SD Day=34**

| **Variable** | **N** | **Mean** | **Std Dev** | **Minimum** | **Maximum** |
| --- | --- | --- | --- | --- | --- |
| timeA timeX ratioxa | 20 20 20 | 29.4300000 75.3800000 2.7789372 | 9.4145520 31.8943750 1.5188021 | 14.5000000 26.4000000 0.8235294 | 49.4000000 127.6000000 7.1862069 |

**Group=SD Day=38**

| **Variable** | **N** | **Mean** | **Std Dev** | **Minimum** | **Maximum** |
| --- | --- | --- | --- | --- | --- |
| timeA timeX ratioxa | 20 20 20 | 35.7350000 51.5550000 1.6873956 | 14.1757493 15.6109771 0.8830547 | 17.7000000 27.8000000 0.5092593 | 75.6000000 78.5000000 3.8480392 |

| **Number of Observations Read** | 836 |
| --- | --- |
| **Number of Observations Used** | 490 |
| **Number of Observations with Missing Values** | 346 |

| **Analysis of Variance** | | | | | |
| --- | --- | --- | --- | --- | --- |
| **Source** | **DF** | **Sum of Squares** | **Mean Square** | **F Value** | **Pr > F** |
| **Model** | 1 | 962443 | 962443 | 3457.57 | <.0001 |
| **Error** | 488 | 135839 | 278.35804 |  |  |
| **Corrected Total** | 489 | 1098282 |  |  |  |

| **Root MSE** | 16.68407 | **R-Square** | 0.8763 |
| --- | --- | --- | --- |
| **Dependent Mean** | 310.46916 | **Adj R-Sq** | 0.8761 |
| **Coeff Var** | 5.37382 |  |  |

| **Parameter Estimates** | | | | | |
| --- | --- | --- | --- | --- | --- |
| **Variable** | **DF** | **Parameter Estimate** | **Standard Error** | **t Value** | **Pr > |t|** |
| **Intercept** | 1 | 242.22798 | 1.38381 | 175.04 | <.0001 |
| **Day** | 1 | 4.90296 | 0.08338 | 58.80 | <.0001 |

| **Number of Observations Read** | 836 |
| --- | --- |
| **Number of Observations Used** | 490 |
| **Number of Observations with Missing Values** | 346 |

| **Analysis of Variance** | | | | | |
| --- | --- | --- | --- | --- | --- |
| **Source** | **DF** | **Sum of Squares** | **Mean Square** | **F Value** | **Pr > F** |
| **Model** | 2 | 993228 | 496614 | 2302.16 | <.0001 |
| **Error** | 487 | 105054 | 215.71658 |  |  |
| **Corrected Total** | 489 | 1098282 |  |  |  |

| **Root MSE** | 14.68729 | **R-Square** | 0.9043 |
| --- | --- | --- | --- |
| **Dependent Mean** | 310.46916 | **Adj R-Sq** | 0.9040 |
| **Coeff Var** | 4.73068 |  |  |

| **Parameter Estimates** | | | | | |
| --- | --- | --- | --- | --- | --- |
| **Variable** | **DF** | **Parameter Estimate** | **Standard Error** | **t Value** | **Pr > |t|** |
| **Intercept** | 1 | 226.27869 | 1.80735 | 125.20 | <.0001 |
| **Day** | 1 | 7.69666 | 0.24511 | 31.40 | <.0001 |
| **d2** | 1 | -0.08327 | 0.00697 | -11.95 | <.0001 |

| **Number of Observations Read** | 988 |
| --- | --- |
| **Number of Observations Used** | 358 |
| **Number of Observations with Missing Values** | 630 |

| **Analysis of Variance** | | | | | |
| --- | --- | --- | --- | --- | --- |
| **Source** | **DF** | **Sum of Squares** | **Mean Square** | **F Value** | **Pr > F** |
| **Model** | 1 | 445068 | 445068 | 1860.87 | <.0001 |
| **Error** | 356 | 85145 | 239.17200 |  |  |
| **Corrected Total** | 357 | 530213 |  |  |  |

| **Root MSE** | 15.46519 | **R-Square** | 0.8394 |
| --- | --- | --- | --- |
| **Dependent Mean** | 297.86291 | **Adj R-Sq** | 0.8390 |
| **Coeff Var** | 5.19205 |  |  |

| **Parameter Estimates** | | | | | |
| --- | --- | --- | --- | --- | --- |
| **Variable** | **DF** | **Parameter Estimate** | **Standard Error** | **t Value** | **Pr > |t|** |
| **Intercept** | 1 | 241.93089 | 1.53272 | 157.84 | <.0001 |
| **Day** | 1 | 3.68353 | 0.08539 | 43.14 | <.0001 |

| **Number of Observations Read** | 988 |
| --- | --- |
| **Number of Observations Used** | 358 |
| **Number of Observations with Missing Values** | 630 |

| **Analysis of Variance** | | | | | |
| --- | --- | --- | --- | --- | --- |
| **Source** | **DF** | **Sum of Squares** | **Mean Square** | **F Value** | **Pr > F** |
| **Model** | 2 | 461210 | 230605 | 1186.39 | <.0001 |
| **Error** | 355 | 69003 | 194.37523 |  |  |
| **Corrected Total** | 357 | 530213 |  |  |  |

| **Root MSE** | 13.94185 | **R-Square** | 0.8699 |
| --- | --- | --- | --- |
| **Dependent Mean** | 297.86291 | **Adj R-Sq** | 0.8691 |
| **Coeff Var** | 4.68063 |  |  |

| **Parameter Estimates** | | | | | |
| --- | --- | --- | --- | --- | --- |
| **Variable** | **DF** | **Parameter Estimate** | **Standard Error** | **t Value** | **Pr > |t|** |
| **Intercept** | 1 | 227.21739 | 2.12510 | 106.92 | <.0001 |
| **Day** | 1 | 6.06111 | 0.27202 | 22.28 | <.0001 |
| **d2** | 1 | -0.06639 | 0.00728 | -9.11 | <.0001 |

| **Model Information** | |
| --- | --- |
| **Data Set** | WORK.DAILY2 |
| **Dependent Variable** | wt |
| **Covariance Structure** | Autoregressive |
| **Subject Effect** | ID(Group) |
| **Estimation Method** | REML |
| **Residual Variance Method** | Profile |
| **Fixed Effects SE Method** | Kenward-Roger |
| **Degrees of Freedom Method** | Kenward-Roger |

| **Class Level Information** | | |
| --- | --- | --- |
| **Class** | **Levels** | **Values** |
| **Group** | 2 | HFD SD |
| **ID** | 48 | C1 C10 C11 C12 C13 C14 C15 C16 C17 C18 C19 C2 C20 C21 C22 C23 C24 C25 C26 C3 C4 C5 C6 C7 C8 C9 T1 T10 T11 T12 T13 T14 T15 T17 T2 T21 T22 T23 T24 T26 T27 T3 T4 T5 T6 T7 T8 T9 |

| **Dimensions** | |
| --- | --- |
| **Covariance Parameters** | 2 |
| **Columns in X** | 7 |
| **Columns in Z** | 0 |
| **Subjects** | 48 |
| **Max Obs per Subject** | 28 |

| **Number of Observations** | |
| --- | --- |
| **Number of Observations Read** | 1824 |
| **Number of Observations Used** | 848 |
| **Number of Observations Not Used** | 976 |

| **Iteration History** | | | |
| --- | --- | --- | --- |
| **Iteration** | **Evaluations** | **-2 Res Log Like** | **Criterion** |
| **0** | 1 | 6937.41020785 |  |
| **1** | 2 | 5533.68071831 | 0.03907800 |
| **2** | 1 | 5479.71848541 | 0.00027136 |
| **3** | 1 | 5479.20440672 | 0.00000074 |
| **4** | 1 | 5479.20295238 | 0.00000000 |

| Convergence criteria met. |
| --- |

| **Covariance Parameter Estimates** | | |
| --- | --- | --- |
| **Cov Parm** | **Subject** | **Estimate** |
| **AR(1)** | ID(Group) | 0.9520 |
| **Residual** |  | 273.61 |

| **Fit Statistics** | |
| --- | --- |
| **-2 Res Log Likelihood** | 5479.2 |
| **AIC (Smaller is Better)** | 5483.2 |
| **AICC (Smaller is Better)** | 5483.2 |
| **BIC (Smaller is Better)** | 5486.9 |

| **Null Model Likelihood Ratio Test** | | |
| --- | --- | --- |
| **DF** | **Chi-Square** | **Pr > ChiSq** |
| 1 | 1458.21 | <.0001 |

| **Solution for Fixed Effects** | | | | | | |
| --- | --- | --- | --- | --- | --- | --- |
| **Effect** | **Group** | **Estimate** | **Standard Error** | **DF** | **t Value** | **Pr > |t|** |
| **Intercept** |  | 226.99 | 3.8472 | 154 | 59.00 | <.0001 |
| **Group** | HFD | -3.4900 | 5.0951 | 109 | -0.68 | 0.4948 |
| **Group** | SD | 0 | . | . | . | . |
| **Day** |  | 6.6556 | 0.2874 | 599 | 23.16 | <.0001 |
| **Day*Group** | HFD | 1.0816 | 0.1918 | 285 | 5.64 | <.0001 |
| **Day*Group** | SD | 0 | . | . | . | . |
| **d2** |  | -0.07949 | 0.006452 | 708 | -12.32 | <.0001 |

| **Type 3 Tests of Fixed Effects** | | | | |
| --- | --- | --- | --- | --- |
| **Effect** | **Num DF** | **Den DF** | **F Value** | **Pr > F** |
| **Group** | 1 | 109 | 0.47 | 0.4948 |
| **Day** | 1 | 651 | 751.05 | <.0001 |
| **Day*Group** | 1 | 285 | 31.79 | <.0001 |
| **d2** | 1 | 708 | 151.77 | <.0001 |

| **Least Squares Means** | | | | | | | | | | | |
| --- | --- | --- | --- | --- | --- | --- | --- | --- | --- | --- | --- |
| **Effect** | **Group** | **Day** | **d2** | **Estimate** | **Standard Error** | **DF** | **t Value** | **Pr > |t|** | **Alpha** | **Lower** | **Upper** |
| **Group** | HFD | 1.00 | 1.00 | 231.15 | 3.5475 | 107 | 65.16 | <.0001 | 0.05 | 224.12 | 238.19 |
| **Group** | SD | 1.00 | 1.00 | 233.56 | 3.6738 | 139 | 63.58 | <.0001 | 0.05 | 226.30 | 240.82 |
| **Group** | HFD | 2.00 | 4.00 | 238.65 | 3.4264 | 97.8 | 69.65 | <.0001 | 0.05 | 231.85 | 245.45 |
| **Group** | SD | 2.00 | 4.00 | 239.98 | 3.5176 | 126 | 68.22 | <.0001 | 0.05 | 233.02 | 246.94 |
| **Group** | HFD | 3.00 | 9.00 | 245.99 | 3.3215 | 90.3 | 74.06 | <.0001 | 0.05 | 239.39 | 252.59 |
| **Group** | SD | 3.00 | 9.00 | 246.24 | 3.3784 | 114 | 72.88 | <.0001 | 0.05 | 239.54 | 252.93 |
| **Group** | HFD | 4.00 | 16.00 | 253.17 | 3.2318 | 84.2 | 78.34 | <.0001 | 0.05 | 246.75 | 259.60 |
| **Group** | SD | 4.00 | 16.00 | 252.34 | 3.2558 | 104 | 77.50 | <.0001 | 0.05 | 245.88 | 258.79 |
| **Group** | HFD | 5.00 | 25.00 | 260.19 | 3.1563 | 79.2 | 82.44 | <.0001 | 0.05 | 253.91 | 266.48 |
| **Group** | SD | 5.00 | 25.00 | 258.28 | 3.1492 | 96.2 | 82.01 | <.0001 | 0.05 | 252.03 | 264.53 |
| **Group** | HFD | 6.00 | 36.00 | 267.06 | 3.0938 | 75.3 | 86.32 | <.0001 | 0.05 | 260.89 | 273.22 |
| **Group** | SD | 6.00 | 36.00 | 264.06 | 3.0576 | 89.4 | 86.36 | <.0001 | 0.05 | 257.98 | 270.13 |
| **Group** | HFD | 7.00 | 49.00 | 273.76 | 3.0431 | 72.3 | 89.96 | <.0001 | 0.05 | 267.70 | 279.83 |
| **Group** | SD | 7.00 | 49.00 | 269.68 | 2.9801 | 84 | 90.49 | <.0001 | 0.05 | 263.75 | 275.61 |
| **Group** | HFD | 8.00 | 64.00 | 280.31 | 3.0028 | 70 | 93.35 | <.0001 | 0.05 | 274.32 | 286.29 |
| **Group** | SD | 8.00 | 64.00 | 275.14 | 2.9155 | 79.6 | 94.37 | <.0001 | 0.05 | 269.34 | 280.95 |
| **Group** | HFD | 9.00 | 81.00 | 286.69 | 2.9716 | 68.3 | 96.48 | <.0001 | 0.05 | 280.76 | 292.62 |
| **Group** | SD | 9.00 | 81.00 | 280.45 | 2.8626 | 76.2 | 97.97 | <.0001 | 0.05 | 274.75 | 286.15 |
| **Group** | HFD | 10.00 | 100.00 | 292.92 | 2.9481 | 67.2 | 99.36 | <.0001 | 0.05 | 287.03 | 298.80 |
| **Group** | SD | 10.00 | 100.00 | 285.59 | 2.8200 | 73.6 | 101.27 | <.0001 | 0.05 | 279.97 | 291.21 |
| **Group** | HFD | 11.00 | 121.00 | 298.99 | 2.9309 | 66.5 | 102.01 | <.0001 | 0.05 | 293.14 | 304.84 |
| **Group** | SD | 11.00 | 121.00 | 290.58 | 2.7864 | 71.7 | 104.28 | <.0001 | 0.05 | 285.02 | 296.13 |
| **Group** | HFD | 12.00 | 144.00 | 304.90 | 2.9190 | 66.1 | 104.45 | <.0001 | 0.05 | 299.07 | 310.72 |
| **Group** | SD | 12.00 | 144.00 | 295.41 | 2.7605 | 70.3 | 107.01 | <.0001 | 0.05 | 289.90 | 300.91 |
| **Group** | HFD | 13.00 | 169.00 | 310.65 | 2.9112 | 65.9 | 106.71 | <.0001 | 0.05 | 304.83 | 316.46 |
| **Group** | SD | 13.00 | 169.00 | 300.07 | 2.7409 | 69.3 | 109.48 | <.0001 | 0.05 | 294.61 | 305.54 |
| **Group** | HFD | 14.00 | 196.00 | 316.24 | 2.9066 | 65.9 | 108.80 | <.0001 | 0.05 | 310.43 | 322.04 |
| **Group** | SD | 14.00 | 196.00 | 304.58 | 2.7264 | 68.7 | 111.72 | <.0001 | 0.05 | 299.14 | 310.02 |
| **Group** | HFD | 15.00 | 225.00 | 321.67 | 2.9044 | 65.9 | 110.75 | <.0001 | 0.05 | 315.87 | 327.47 |
| **Group** | SD | 15.00 | 225.00 | 308.93 | 2.7160 | 68.3 | 113.75 | <.0001 | 0.05 | 303.51 | 314.35 |
| **Group** | HFD | 16.00 | 256.00 | 326.94 | 2.9040 | 66.1 | 112.58 | <.0001 | 0.05 | 321.14 | 332.74 |
| **Group** | SD | 16.00 | 256.00 | 313.13 | 2.7088 | 68.1 | 115.60 | <.0001 | 0.05 | 307.72 | 318.53 |
| **Group** | HFD | 17.00 | 289.00 | 332.06 | 2.9049 | 66.3 | 114.31 | <.0001 | 0.05 | 326.26 | 337.86 |
| **Group** | SD | 17.00 | 289.00 | 317.16 | 2.7039 | 68 | 117.30 | <.0001 | 0.05 | 311.76 | 322.55 |
| **Group** | HFD | 18.00 | 324.00 | 337.01 | 2.9068 | 66.5 | 115.94 | <.0001 | 0.05 | 331.21 | 342.81 |
| **Group** | SD | 18.00 | 324.00 | 321.03 | 2.7009 | 67.9 | 118.86 | <.0001 | 0.05 | 315.64 | 326.42 |
| **Group** | HFD | 19.00 | 361.00 | 341.81 | 2.9097 | 66.6 | 117.47 | <.0001 | 0.05 | 336.00 | 347.62 |
| **Group** | SD | 19.00 | 361.00 | 324.75 | 2.6993 | 67.9 | 120.31 | <.0001 | 0.05 | 319.36 | 330.13 |
| **Group** | HFD | 20.00 | 400.00 | 346.44 | 2.9135 | 66.8 | 118.91 | <.0001 | 0.05 | 340.63 | 352.26 |
| **Group** | SD | 20.00 | 400.00 | 328.30 | 2.6988 | 67.9 | 121.65 | <.0001 | 0.05 | 322.92 | 333.69 |
| **Group** | HFD | 21.00 | 441.00 | 350.92 | 2.9185 | 67 | 120.24 | <.0001 | 0.05 | 345.10 | 356.75 |
| **Group** | SD | 21.00 | 441.00 | 331.70 | 2.6994 | 68 | 122.88 | <.0001 | 0.05 | 326.31 | 337.08 |
| **Group** | HFD | 22.00 | 484.00 | 355.24 | 2.9252 | 67.2 | 121.44 | <.0001 | 0.05 | 349.40 | 361.08 |
| **Group** | SD | 22.00 | 484.00 | 334.94 | 2.7013 | 68 | 123.99 | <.0001 | 0.05 | 329.55 | 340.33 |
| **Group** | HFD | 23.00 | 529.00 | 359.40 | 2.9339 | 67.5 | 122.50 | <.0001 | 0.05 | 353.55 | 365.26 |
| **Group** | SD | 23.00 | 529.00 | 338.01 | 2.7048 | 68.1 | 124.97 | <.0001 | 0.05 | 332.62 | 343.41 |
| **Group** | HFD | 24.00 | 576.00 | 363.40 | 2.9455 | 67.9 | 123.37 | <.0001 | 0.05 | 357.53 | 369.28 |
| **Group** | SD | 24.00 | 576.00 | 340.93 | 2.7104 | 68.3 | 125.79 | <.0001 | 0.05 | 335.53 | 346.34 |
| **Group** | HFD | 25.00 | 625.00 | 367.25 | 2.9609 | 68.5 | 124.03 | <.0001 | 0.05 | 361.34 | 373.15 |
| **Group** | SD | 25.00 | 625.00 | 343.69 | 2.7187 | 68.6 | 126.42 | <.0001 | 0.05 | 338.27 | 349.12 |
| **Group** | HFD | 26.00 | 676.00 | 370.93 | 2.9810 | 69.3 | 124.43 | <.0001 | 0.05 | 364.98 | 376.88 |
| **Group** | SD | 26.00 | 676.00 | 346.30 | 2.7306 | 69.1 | 126.82 | <.0001 | 0.05 | 340.85 | 351.74 |
| **Group** | HFD | 27.00 | 729.00 | 374.45 | 3.0069 | 70.5 | 124.53 | <.0001 | 0.05 | 368.46 | 380.45 |
| **Group** | SD | 27.00 | 729.00 | 348.74 | 2.7471 | 69.9 | 126.95 | <.0001 | 0.05 | 343.26 | 354.22 |
| **Group** | HFD | 28.00 | 784.00 | 377.82 | 3.0400 | 72.2 | 124.28 | <.0001 | 0.05 | 371.76 | 383.88 |
| **Group** | SD | 28.00 | 784.00 | 351.02 | 2.7693 | 71.1 | 126.75 | <.0001 | 0.05 | 345.50 | 356.54 |
| **Group** | HFD | 29.00 | 841.00 | 381.03 | 3.0814 | 74.3 | 123.65 | <.0001 | 0.05 | 374.89 | 387.16 |
| **Group** | SD | 29.00 | 841.00 | 353.15 | 2.7985 | 72.8 | 126.19 | <.0001 | 0.05 | 347.57 | 358.73 |
| **Group** | HFD | 30.00 | 900.00 | 384.07 | 3.1324 | 77.1 | 122.61 | <.0001 | 0.05 | 377.84 | 390.31 |
| **Group** | SD | 30.00 | 900.00 | 355.11 | 2.8360 | 75.1 | 125.22 | <.0001 | 0.05 | 349.46 | 360.76 |
| **Group** | HFD | 31.00 | 961.00 | 386.96 | 3.1945 | 80.7 | 121.13 | <.0001 | 0.05 | 380.60 | 393.32 |
| **Group** | SD | 31.00 | 961.00 | 356.92 | 2.8832 | 78.1 | 123.79 | <.0001 | 0.05 | 351.18 | 362.66 |
| **Group** | HFD | 32.00 | 1024.0 | 389.69 | 3.2688 | 85.1 | 119.21 | <.0001 | 0.05 | 383.19 | 396.19 |
| **Group** | SD | 32.00 | 1024.0 | 358.57 | 2.9414 | 82 | 121.91 | <.0001 | 0.05 | 352.72 | 364.42 |
| **Group** | HFD | 33.00 | 1089.0 | 392.26 | 3.3566 | 90.6 | 116.86 | <.0001 | 0.05 | 385.59 | 398.93 |
| **Group** | SD | 33.00 | 1089.0 | 360.06 | 3.0119 | 86.9 | 119.55 | <.0001 | 0.05 | 354.07 | 366.04 |
| **Group** | HFD | 34.00 | 1156.0 | 394.67 | 3.4588 | 97.3 | 114.11 | <.0001 | 0.05 | 387.81 | 401.54 |
| **Group** | SD | 34.00 | 1156.0 | 361.39 | 3.0960 | 93.1 | 116.73 | <.0001 | 0.05 | 355.24 | 367.53 |
| **Group** | HFD | 35.00 | 1225.0 | 396.93 | 3.5764 | 105 | 110.99 | <.0001 | 0.05 | 389.83 | 404.02 |
| **Group** | SD | 35.00 | 1225.0 | 362.56 | 3.1947 | 101 | 113.49 | <.0001 | 0.05 | 356.22 | 368.89 |
| **Group** | HFD | 36.00 | 1296.0 | 399.02 | 3.7101 | 115 | 107.55 | <.0001 | 0.05 | 391.67 | 406.37 |
| **Group** | SD | 36.00 | 1296.0 | 363.57 | 3.3089 | 110 | 109.88 | <.0001 | 0.05 | 357.01 | 370.13 |
| **Group** | HFD | 37.00 | 1369.0 | 400.95 | 3.8605 | 125 | 103.86 | <.0001 | 0.05 | 393.31 | 408.59 |
| **Group** | SD | 37.00 | 1369.0 | 364.42 | 3.4394 | 121 | 105.95 | <.0001 | 0.05 | 357.61 | 371.23 |
| **Group** | HFD | 38.00 | 1444.0 | 402.73 | 4.0279 | 138 | 99.98 | <.0001 | 0.05 | 394.76 | 410.69 |
| **Group** | SD | 38.00 | 1444.0 | 365.12 | 3.5868 | 133 | 101.79 | <.0001 | 0.05 | 358.02 | 372.21 |

| **Differences of Least Squares Means** | | | | | | | | | | | | |
| --- | --- | --- | --- | --- | --- | --- | --- | --- | --- | --- | --- | --- |
| **Effect** | **Group** | **_Group** | **Day** | **d2** | **Estimate** | **Standard Error** | **DF** | **t Value** | **Pr > |t|** | **Alpha** | **Lower** | **Upper** |
| **Group** | HFD | SD | 1.00 | 1.00 | -2.4083 | 4.9611 | 104 | -0.49 | 0.6284 | 0.05 | -12.2461 | 7.4295 |
| **Group** | HFD | SD | 2.00 | 4.00 | -1.3267 | 4.8310 | 99.7 | -0.27 | 0.7842 | 0.05 | -10.9116 | 8.2582 |
| **Group** | HFD | SD | 3.00 | 9.00 | -0.2450 | 4.7051 | 95.3 | -0.05 | 0.9586 | 0.05 | -9.5855 | 9.0954 |
| **Group** | HFD | SD | 4.00 | 16.00 | 0.8366 | 4.5838 | 91 | 0.18 | 0.8556 | 0.05 | -8.2685 | 9.9418 |
| **Group** | HFD | SD | 5.00 | 25.00 | 1.9183 | 4.4674 | 86.8 | 0.43 | 0.6687 | 0.05 | -6.9614 | 10.7980 |
| **Group** | HFD | SD | 6.00 | 36.00 | 2.9999 | 4.3564 | 82.8 | 0.69 | 0.4930 | 0.05 | -5.6650 | 11.6649 |
| **Group** | HFD | SD | 7.00 | 49.00 | 4.0816 | 4.2511 | 79 | 0.96 | 0.3399 | 0.05 | -4.3800 | 12.5432 |
| **Group** | HFD | SD | 8.00 | 64.00 | 5.1632 | 4.1520 | 75.4 | 1.24 | 0.2175 | 0.05 | -3.1073 | 13.4338 |
| **Group** | HFD | SD | 9.00 | 81.00 | 6.2449 | 4.0596 | 72 | 1.54 | 0.1284 | 0.05 | -1.8477 | 14.3375 |
| **Group** | HFD | SD | 10.00 | 100.00 | 7.3265 | 3.9742 | 68.9 | 1.84 | 0.0696 | 0.05 | -0.6021 | 15.2551 |
| **Group** | HFD | SD | 11.00 | 121.00 | 8.4082 | 3.8965 | 66.1 | 2.16 | 0.0346 | 0.05 | 0.6287 | 16.1876 |
| **Group** | HFD | SD | 12.00 | 144.00 | 9.4898 | 3.8268 | 63.6 | 2.48 | 0.0158 | 0.05 | 1.8438 | 17.1358 |
| **Group** | HFD | SD | 13.00 | 169.00 | 10.5715 | 3.7656 | 61.4 | 2.81 | 0.0067 | 0.05 | 3.0425 | 18.1004 |
| **Group** | HFD | SD | 14.00 | 196.00 | 11.6531 | 3.7134 | 59.5 | 3.14 | 0.0026 | 0.05 | 4.2240 | 19.0822 |
| **Group** | HFD | SD | 15.00 | 225.00 | 12.7348 | 3.6704 | 58 | 3.47 | 0.0010 | 0.05 | 5.3876 | 20.0819 |
| **Group** | HFD | SD | 16.00 | 256.00 | 13.8164 | 3.6370 | 56.8 | 3.80 | 0.0004 | 0.05 | 6.5329 | 21.1000 |
| **Group** | HFD | SD | 17.00 | 289.00 | 14.8981 | 3.6135 | 55.9 | 4.12 | 0.0001 | 0.05 | 7.6592 | 22.1370 |
| **Group** | HFD | SD | 18.00 | 324.00 | 15.9797 | 3.6001 | 55.5 | 4.44 | <.0001 | 0.05 | 8.7663 | 23.1931 |
| **Group** | HFD | SD | 19.00 | 361.00 | 17.0614 | 3.5969 | 55.4 | 4.74 | <.0001 | 0.05 | 9.8541 | 24.2687 |
| **Group** | HFD | SD | 20.00 | 400.00 | 18.1430 | 3.6039 | 55.6 | 5.03 | <.0001 | 0.05 | 10.9224 | 25.3637 |
| **Group** | HFD | SD | 21.00 | 441.00 | 19.2247 | 3.6211 | 56.2 | 5.31 | <.0001 | 0.05 | 11.9713 | 26.4780 |
| **Group** | HFD | SD | 22.00 | 484.00 | 20.3063 | 3.6483 | 57.2 | 5.57 | <.0001 | 0.05 | 13.0012 | 27.6114 |
| **Group** | HFD | SD | 23.00 | 529.00 | 21.3880 | 3.6853 | 58.5 | 5.80 | <.0001 | 0.05 | 14.0124 | 28.7636 |
| **Group** | HFD | SD | 24.00 | 576.00 | 22.4696 | 3.7318 | 60.2 | 6.02 | <.0001 | 0.05 | 15.0053 | 29.9339 |
| **Group** | HFD | SD | 25.00 | 625.00 | 23.5513 | 3.7875 | 62.2 | 6.22 | <.0001 | 0.05 | 15.9806 | 31.1219 |
| **Group** | HFD | SD | 26.00 | 676.00 | 24.6329 | 3.8519 | 64.5 | 6.39 | <.0001 | 0.05 | 16.9390 | 32.3268 |
| **Group** | HFD | SD | 27.00 | 729.00 | 25.7146 | 3.9247 | 67.1 | 6.55 | <.0001 | 0.05 | 17.8812 | 33.5479 |
| **Group** | HFD | SD | 28.00 | 784.00 | 26.7962 | 4.0053 | 70.1 | 6.69 | <.0001 | 0.05 | 18.8081 | 34.7844 |
| **Group** | HFD | SD | 29.00 | 841.00 | 27.8779 | 4.0933 | 73.3 | 6.81 | <.0001 | 0.05 | 19.7204 | 36.0353 |
| **Group** | HFD | SD | 30.00 | 900.00 | 28.9595 | 4.1883 | 76.7 | 6.91 | <.0001 | 0.05 | 20.6191 | 37.2999 |
| **Group** | HFD | SD | 31.00 | 961.00 | 30.0412 | 4.2897 | 80.4 | 7.00 | <.0001 | 0.05 | 21.5050 | 38.5773 |
| **Group** | HFD | SD | 32.00 | 1024.0 | 31.1228 | 4.3972 | 84.3 | 7.08 | <.0001 | 0.05 | 22.3790 | 39.8667 |
| **Group** | HFD | SD | 33.00 | 1089.0 | 32.2045 | 4.5103 | 88.4 | 7.14 | <.0001 | 0.05 | 23.2418 | 41.1672 |
| **Group** | HFD | SD | 34.00 | 1156.0 | 33.2861 | 4.6286 | 92.6 | 7.19 | <.0001 | 0.05 | 24.0942 | 42.4780 |
| **Group** | HFD | SD | 35.00 | 1225.0 | 34.3678 | 4.7516 | 97 | 7.23 | <.0001 | 0.05 | 24.9371 | 43.7985 |
| **Group** | HFD | SD | 36.00 | 1296.0 | 35.4494 | 4.8792 | 101 | 7.27 | <.0001 | 0.05 | 25.7710 | 45.1278 |
| **Group** | HFD | SD | 37.00 | 1369.0 | 36.5311 | 5.0108 | 106 | 7.29 | <.0001 | 0.05 | 26.5967 | 46.4654 |
| **Group** | HFD | SD | 38.00 | 1444.0 | 37.6127 | 5.1462 | 111 | 7.31 | <.0001 | 0.05 | 27.4148 | 47.8106 |

| **Obs** | **ID** | **Group** | **noresponse** | **Day** | **Week** | **wt** | **glucose** | **ketone** | **distance** | **distDay** | **timeA** | **timeX** |
| --- | --- | --- | --- | --- | --- | --- | --- | --- | --- | --- | --- | --- |
| **1** | T1 | HFD | 0 | 1 | 1 | 230.67 | 103 | 1.0 | . | . | . | . |
| **2** | T1 | HFD | 0 | 2 | 1 | 242.43 | . | . | . | . | . | . |
| **3** | T1 | HFD | 0 | 3 | 1 | 254.01 | . | . | . | . | . | . |
| **4** | T1 | HFD | 0 | 4 | 1 | 262.39 | . | . | . | . | . | . |
| **5** | T1 | HFD | 0 | 5 | 1 | 265.74 | 96 | 0.5 | . | . | . | . |
| **6** | T1 | HFD | 0 | 6 | 1 | 271.32 | . | . | . | . | . | . |
| **7** | T1 | HFD | 0 | 7 | 1 | 288.36 | . | . | . | . | . | . |
| **8** | T1 | HFD | 0 | 8 | 2 | 290.35 | 109 | 0.7 | . | . | . | . |
| **9** | T1 | HFD | 0 | 9 | 2 | 302.28 | . | . | . | . | . | . |
| **10** | T1 | HFD | 0 | 10 | 2 | 310.84 | . | . | . | . | . | . |

| **Obs** | **NORday** | **calintake** | **wk_wt** | **wk_glucose** | **wk_ketone** | **wk_distance** | **wk_timeA** | **wk_timeX** | **wk_calintake** |
| --- | --- | --- | --- | --- | --- | --- | --- | --- | --- |
| **1** | . | . | 259.274 | 99.5 | 0.75 | . | . | . | . |
| **2** | . | . | 259.274 | 99.5 | 0.75 | . | . | . | . |
| **3** | . | . | 259.274 | 99.5 | 0.75 | . | . | . | . |
| **4** | . | . | 259.274 | 99.5 | 0.75 | . | . | . | . |
| **5** | . | . | 259.274 | 99.5 | 0.75 | . | . | . | . |
| **6** | . | . | 259.274 | 99.5 | 0.75 | . | . | . | . |
| **7** | . | . | 259.274 | 99.5 | 0.75 | . | . | . | . |
| **8** | . | . | 313.487 | 97.0 | 0.75 | 3.76845 | . | . | . |
| **9** | . | . | 313.487 | 97.0 | 0.75 | 3.76845 | . | . | . |
| **10** | . | . | 313.487 | 97.0 | 0.75 | 3.76845 | . | . | . |

| **Obs** | **d2** | **Pred** | **StdErrPred** | **DF** | **Alpha** | **Lower** | **Upper** | **Resid** |
| --- | --- | --- | --- | --- | --- | --- | --- | --- |
| **1** | 1 | 231.153 | 3.54746 | 106.888 | 0.05 | 224.120 | 238.185 | -0.4830 |
| **2** | 4 | 238.652 | 3.42644 | 97.833 | 0.05 | 231.852 | 245.452 | 3.7782 |
| **3** | 9 | 245.992 | 3.32151 | 90.310 | 0.05 | 239.393 | 252.590 | 8.0184 |
| **4** | 16 | 253.172 | 3.23180 | 84.155 | 0.05 | 246.746 | 259.599 | 9.2176 |
| **5** | 25 | 260.194 | 3.15629 | 79.202 | 0.05 | 253.912 | 266.476 | 5.5458 |
| **6** | 36 | 267.057 | 3.09382 | 75.290 | 0.05 | 260.894 | 273.220 | 4.2629 |
| **7** | 49 | 273.761 | 3.04312 | 72.268 | 0.05 | 267.695 | 279.827 | 14.5990 |
| **8** | 64 | 280.306 | 3.00283 | 69.997 | 0.05 | 274.317 | 286.295 | 10.0441 |
| **9** | 81 | 286.692 | 2.97159 | 68.349 | 0.05 | 280.763 | 292.621 | 15.5882 |
| **10** | 100 | 292.919 | 2.94805 | 67.209 | 0.05 | 287.035 | 298.803 | 17.9212 |

| **Moments** | | | |
| --- | --- | --- | --- |
| **N** | 848 | **Sum Weights** | 848 |
| **Mean** | -1.2500104 | **Sum Observations** | -1060.0088 |
| **Std Deviation** | 14.7079204 | **Variance** | 216.322921 |
| **Skewness** | -0.2632694 | **Kurtosis** | 0.18601334 |
| **Uncorrected SS** | 184550.536 | **Corrected SS** | 183225.514 |
| **Coeff Variation** | -1176.6238 | **Std Error Mean** | 0.50507206 |

| **Basic Statistical Measures** | | | |
| --- | --- | --- | --- |
| **Location** | | **Variability** | |
| **Mean** | -1.2500 | **Std Deviation** | 14.70792 |
| **Median** | -0.1833 | **Variance** | 216.32292 |
| **Mode** | -11.9292 | **Range** | 100.81723 |
|  |  | **Interquartile Range** | 17.48522 |

| ***Note: The mode displayed is the smallest of 4 modes with a count of 3.*** |
| --- |

| **Tests for Location: Mu0=0** | | | | |
| --- | --- | --- | --- | --- |
| **Test** | **Statistic** | | **p Value** | |
| **Student's t** | **t** | -2.47492 | **Pr > |t|** | 0.0135 |
| **Sign** | **M** | -7 | **Pr >= |M|** | 0.6553 |
| **Signed Rank** | **S** | -11713 | **Pr >= |S|** | 0.1007 |

| **Tests for Normality** | | | | |
| --- | --- | --- | --- | --- |
| **Test** | **Statistic** | | **p Value** | |
| **Shapiro-Wilk** | **W** | 0.992161 | **Pr < W** | 0.0002 |
| **Kolmogorov-Smirnov** | **D** | 0.046161 | **Pr > D** | <0.0100 |
| **Cramer-von Mises** | **W-Sq** | 0.412786 | **Pr > W-Sq** | <0.0050 |
| **Anderson-Darling** | **A-Sq** | 2.460043 | **Pr > A-Sq** | <0.0050 |

| **Quantiles (Definition 5)** | |
| --- | --- |
| **Level** | **Quantile** |
| **100% Max** | 46.430791 |
| **99%** | 31.293884 |
| **95%** | 22.192583 |
| **90%** | 17.193164 |
| **75% Q3** | 8.059968 |
| **50% Median** | -0.183295 |
| **25% Q1** | -9.425253 |
| **10%** | -22.180974 |
| **5%** | -28.091255 |
| **1%** | -35.402807 |
| **0% Min** | -54.386438 |

| **Extreme Observations** | | | |
| --- | --- | --- | --- |
| **Lowest** | | **Highest** | |
| **Value** | **Obs** | **Value** | **Obs** |
| -54.3864 | 1174 | 32.4308 | 1556 |
| -50.1161 | 1178 | 34.7012 | 162 |
| -48.2963 | 1166 | 35.2712 | 494 |
| -40.5623 | 365 | 40.2610 | 1737 |
| -38.1989 | 370 | 46.4308 | 1746 |

| **Missing Values** | | | |
| --- | --- | --- | --- |
| **Missing Value** | **Count** | **Percent Of** | |
|  |  | **All Obs** | **Missing Obs** |
| . | 976 | 53.51 | 100.00 |

| **Model Information** | |
| --- | --- |
| **Data Set** | WORK.DAYSHORT |
| **Dependent Variable** | transratio |
| **Covariance Structure** | Unstructured |
| **Subject Effect** | ID(Group) |
| **Estimation Method** | REML |
| **Residual Variance Method** | None |
| **Fixed Effects SE Method** | Model-Based |
| **Degrees of Freedom Method** | Between-Within |

| **Class Level Information** | | |
| --- | --- | --- |
| **Class** | **Levels** | **Values** |
| **Group** | 2 | HFD SD |
| **ID** | 32 | C10 C11 C12 C13 C14 C15 C16 C17 C18 C19 C20 C21 C22 C23 C24 C25 C26 C7 C8 C9 T11 T12 T13 T14 T15 T17 T21 T22 T23 T24 T26 T27 |

| **Dimensions** | |
| --- | --- |
| **Covariance Parameters** | 10 |
| **Columns in X** | 6 |
| **Columns in Z** | 0 |
| **Subjects** | 32 |
| **Max Obs per Subject** | 4 |

| **Number of Observations** | |
| --- | --- |
| **Number of Observations Read** | 128 |
| **Number of Observations Used** | 128 |
| **Number of Observations Not Used** | 0 |

| **Iteration History** | | | |
| --- | --- | --- | --- |
| **Iteration** | **Evaluations** | **-2 Res Log Like** | **Criterion** |
| **0** | 1 | 285.87354915 |  |
| **1** | 4 | 252.79190369 | 0.01869048 |
| **2** | 1 | 252.53824868 | 0.00070092 |
| **3** | 1 | 252.52935980 | 0.00000150 |
| **4** | 1 | 252.52934125 | 0.00000000 |

| Convergence criteria met. |
| --- |

| **Covariance Parameter Estimates** | | |
| --- | --- | --- |
| **Cov Parm** | **Subject** | **Estimate** |
| **UN(1,1)** | ID(Group) | 1.0267 |
| **UN(2,1)** | ID(Group) | -0.1714 |
| **UN(2,2)** | ID(Group) | 0.2634 |
| **UN(3,1)** | ID(Group) | 0.2817 |
| **UN(3,2)** | ID(Group) | -0.03752 |
| **UN(3,3)** | ID(Group) | 0.4655 |
| **UN(4,1)** | ID(Group) | 0.1574 |
| **UN(4,2)** | ID(Group) | 0.09085 |
| **UN(4,3)** | ID(Group) | 0.1376 |
| **UN(4,4)** | ID(Group) | 0.3208 |

| **Fit Statistics** | |
| --- | --- |
| **-2 Res Log Likelihood** | 252.5 |
| **AIC (Smaller is Better)** | 272.5 |
| **AICC (Smaller is Better)** | 274.5 |
| **BIC (Smaller is Better)** | 287.2 |

| **Null Model Likelihood Ratio Test** | | |
| --- | --- | --- |
| **DF** | **Chi-Square** | **Pr > ChiSq** |
| 9 | 33.34 | 0.0001 |

| **Type 3 Tests of Fixed Effects** | | | | |
| --- | --- | --- | --- | --- |
| **Effect** | **Num DF** | **Den DF** | **F Value** | **Pr > F** |
| **Group** | 1 | 30 | 0.10 | 0.7541 |
| **Day** | 1 | 30 | 2.46 | 0.1276 |
| **Day*Group** | 1 | 30 | 0.49 | 0.4910 |

| **Least Squares Means** | | | | | | | |
| --- | --- | --- | --- | --- | --- | --- | --- |
| **Effect** | **Group** | **Day** | **Estimate** | **Standard Error** | **DF** | **t Value** | **Pr > |t|** |
| **Group** | HFD | 31.00 | 0.2044 | 0.1085 | 30 | 1.88 | 0.0692 |
| **Group** | SD | 31.00 | 0.5265 | 0.08402 | 30 | 6.27 | <.0001 |
| **Group** | HFD | 32.00 | 0.1719 | 0.1030 | 30 | 1.67 | 0.1055 |
| **Group** | SD | 32.00 | 0.5140 | 0.07976 | 30 | 6.44 | <.0001 |
| **Group** | HFD | 34.00 | 0.1068 | 0.1067 | 30 | 1.00 | 0.3249 |
| **Group** | SD | 34.00 | 0.4890 | 0.08261 | 30 | 5.92 | <.0001 |
| **Group** | HFD | 38.00 | -0.02347 | 0.1591 | 30 | -0.15 | 0.8838 |
| **Group** | SD | 38.00 | 0.4390 | 0.1233 | 30 | 3.56 | 0.0013 |

| **Differences of Least Squares Means** | | | | | | | | |
| --- | --- | --- | --- | --- | --- | --- | --- | --- |
| **Effect** | **Group** | **_Group** | **Day** | **Estimate** | **Standard Error** | **DF** | **t Value** | **Pr > |t|** |
| **Group** | HFD | SD | 31.00 | -0.3221 | 0.1372 | 30 | -2.35 | 0.0257 |
| **Group** | HFD | SD | 32.00 | -0.3422 | 0.1302 | 30 | -2.63 | 0.0134 |
| **Group** | HFD | SD | 34.00 | -0.3823 | 0.1349 | 30 | -2.83 | 0.0082 |
| **Group** | HFD | SD | 38.00 | -0.4625 | 0.2013 | 30 | -2.30 | 0.0287 |

| **Moments** | | | |
| --- | --- | --- | --- |
| **N** | 128 | **Sum Weights** | 128 |
| **Mean** | 0.12438929 | **Sum Observations** | 15.9218297 |
| **Std Deviation** | 0.70457909 | **Variance** | 0.49643169 |
| **Skewness** | 0.54698553 | **Kurtosis** | 0.544775 |
| **Uncorrected SS** | 65.0273303 | **Corrected SS** | 63.0468252 |
| **Coeff Variation** | 566.430651 | **Std Error Mean** | 0.06227658 |

| **Basic Statistical Measures** | | | |
| --- | --- | --- | --- |
| **Location** | | **Variability** | |
| **Mean** | 0.124389 | **Std Deviation** | 0.70458 |
| **Median** | 0.077967 | **Variance** | 0.49643 |
| **Mode** | . | **Range** | 3.78836 |
|  |  | **Interquartile Range** | 0.93402 |

| **Tests for Location: Mu0=0** | | | | |
| --- | --- | --- | --- | --- |
| **Test** | **Statistic** | | **p Value** | |
| **Student's t** | **t** | 1.997369 | **Pr > |t|** | 0.0479 |
| **Sign** | **M** | 8 | **Pr >= |M|** | 0.1847 |
| **Signed Rank** | **S** | 623 | **Pr >= |S|** | 0.1391 |

| **Tests for Normality** | | | | |
| --- | --- | --- | --- | --- |
| **Test** | **Statistic** | | **p Value** | |
| **Shapiro-Wilk** | **W** | 0.977929 | **Pr < W** | 0.0347 |
| **Kolmogorov-Smirnov** | **D** | 0.054731 | **Pr > D** | >0.1500 |
| **Cramer-von Mises** | **W-Sq** | 0.075209 | **Pr > W-Sq** | 0.2412 |
| **Anderson-Darling** | **A-Sq** | 0.597205 | **Pr > A-Sq** | 0.1216 |

| **Quantiles (Definition 5)** | |
| --- | --- |
| **Level** | **Quantile** |
| **100% Max** | 2.1714192 |
| **99%** | 2.1461909 |
| **95%** | 1.4555388 |
| **90%** | 1.0242765 |
| **75% Q3** | 0.5292554 |
| **50% Median** | 0.0779666 |
| **25% Q1** | -0.4047694 |
| **10%** | -0.7066423 |
| **5%** | -0.9183991 |
| **1%** | -1.1137981 |
| **0% Min** | -1.6169384 |

| **Extreme Observations** | | | |
| --- | --- | --- | --- |
| **Lowest** | | **Highest** | |
| **Value** | **Obs** | **Value** | **Obs** |
| -1.61694 | 19 | 1.50443 | 75 |
| -1.11380 | 111 | 1.90448 | 9 |
| -1.08379 | 110 | 2.07875 | 7 |
| -1.03371 | 24 | 2.14619 | 27 |
| -1.00239 | 50 | 2.17142 | 11 |

| **Model Information** | |
| --- | --- |
| **Data Set** | WORK.DAYSHORT |
| **Dependent Variable** | timeX |
| **Covariance Structure** | Toeplitz |
| **Subject Effect** | ID(Group) |
| **Estimation Method** | REML |
| **Residual Variance Method** | Profile |
| **Fixed Effects SE Method** | Model-Based |
| **Degrees of Freedom Method** | Between-Within |

| **Class Level Information** | | |
| --- | --- | --- |
| **Class** | **Levels** | **Values** |
| **Group** | 2 | HFD SD |
| **ID** | 32 | C10 C11 C12 C13 C14 C15 C16 C17 C18 C19 C20 C21 C22 C23 C24 C25 C26 C7 C8 C9 T11 T12 T13 T14 T15 T17 T21 T22 T23 T24 T26 T27 |

| **Dimensions** | |
| --- | --- |
| **Covariance Parameters** | 4 |
| **Columns in X** | 6 |
| **Columns in Z** | 0 |
| **Subjects** | 32 |
| **Max Obs per Subject** | 4 |

| **Number of Observations** | |
| --- | --- |
| **Number of Observations Read** | 128 |
| **Number of Observations Used** | 128 |
| **Number of Observations Not Used** | 0 |

| **Iteration History** | | | |
| --- | --- | --- | --- |
| **Iteration** | **Evaluations** | **-2 Res Log Like** | **Criterion** |
| **0** | 1 | 1156.61194815 |  |
| **1** | 2 | 1141.78755323 | 0.00049650 |
| **2** | 1 | 1141.53087734 | 0.00002767 |
| **3** | 1 | 1141.51774805 | 0.00000010 |
| **4** | 1 | 1141.51770083 | 0.00000000 |

| Convergence criteria met. |
| --- |

| **Covariance Parameter Estimates** | | |
| --- | --- | --- |
| **Cov Parm** | **Subject** | **Estimate** |
| **TOEP(2)** | ID(Group) | 132.14 |
| **TOEP(3)** | ID(Group) | 236.62 |
| **TOEP(4)** | ID(Group) | 191.43 |
| **Residual** |  | 568.70 |

| **Fit Statistics** | |
| --- | --- |
| **-2 Res Log Likelihood** | 1141.5 |
| **AIC (Smaller is Better)** | 1149.5 |
| **AICC (Smaller is Better)** | 1149.9 |
| **BIC (Smaller is Better)** | 1155.4 |

| **Null Model Likelihood Ratio Test** | | |
| --- | --- | --- |
| **DF** | **Chi-Square** | **Pr > ChiSq** |
| 3 | 15.09 | 0.0017 |

| **Type 3 Tests of Fixed Effects** | | | | |
| --- | --- | --- | --- | --- |
| **Effect** | **Num DF** | **Den DF** | **F Value** | **Pr > F** |
| **Group** | 1 | 30 | 0.07 | 0.7884 |
| **Day** | 1 | 94 | 1.16 | 0.2838 |
| **Day*Group** | 1 | 94 | 0.43 | 0.5152 |

| **Least Squares Means** | | | | | | | |
| --- | --- | --- | --- | --- | --- | --- | --- |
| **Effect** | **Group** | **Day** | **Estimate** | **Standard Error** | **DF** | **t Value** | **Pr > |t|** |
| **Group** | HFD | 31.00 | 40.7895 | 5.4341 | 30 | 7.51 | <.0001 |
| **Group** | SD | 31.00 | 54.2010 | 4.2092 | 30 | 12.88 | <.0001 |
| **Group** | HFD | 32.00 | 41.0477 | 5.0482 | 30 | 8.13 | <.0001 |
| **Group** | SD | 32.00 | 55.2535 | 3.9104 | 30 | 14.13 | <.0001 |
| **Group** | HFD | 34.00 | 41.5640 | 4.7896 | 30 | 8.68 | <.0001 |
| **Group** | SD | 34.00 | 57.3585 | 3.7100 | 30 | 15.46 | <.0001 |
| **Group** | HFD | 38.00 | 42.5966 | 6.3266 | 30 | 6.73 | <.0001 |
| **Group** | SD | 38.00 | 61.5684 | 4.9006 | 30 | 12.56 | <.0001 |

| **Differences of Least Squares Means** | | | | | | | | |
| --- | --- | --- | --- | --- | --- | --- | --- | --- |
| **Effect** | **Group** | **_Group** | **Day** | **Estimate** | **Standard Error** | **DF** | **t Value** | **Pr > |t|** |
| **Group** | HFD | SD | 31.00 | -13.4115 | 6.8736 | 30 | -1.95 | 0.0604 |
| **Group** | HFD | SD | 32.00 | -14.2059 | 6.3856 | 30 | -2.22 | 0.0338 |
| **Group** | HFD | SD | 34.00 | -15.7945 | 6.0584 | 30 | -2.61 | 0.0141 |
| **Group** | HFD | SD | 38.00 | -18.9718 | 8.0026 | 30 | -2.37 | 0.0244 |

| **7 Variables:** | wk_wt wk_glucose wk_ketone wk_distance wk_timeA wk_timeX ratioxa |
| --- | --- |

| **Simple Statistics** | | | | | | |
| --- | --- | --- | --- | --- | --- | --- |
| **Variable** | **N** | **Mean** | **Std Dev** | **Sum** | **Minimum** | **Maximum** |
| **wk_wt** | 157 | 0 | 0 | 0 | 0 | 0 |
| **wk_glucose** | 157 | 0 | 0 | 0 | 0 | 0 |
| **wk_ketone** | 157 | 0 | 0 | 0 | 0 | 0 |
| **wk_distance** | 157 | 0 | 0 | 0 | 0 | 0 |
| **wk_timeA** | 157 | 0 | 0 | 0 | 0 | 0 |
| **wk_timeX** | 157 | 0 | 0 | 0 | 0 | 0 |
| **ratioxa** | 0 | . | . | . | . | . |

| **Pearson Correlation Coefficients Prob > |r| under H0: Rho=0 Number of Observations** | | | | | | | |
| --- | --- | --- | --- | --- | --- | --- | --- |
|  | **wk_wt** | **wk_glucose** | **wk_ketone** | **wk_distance** | **wk_timeA** | **wk_timeX** | **ratioxa** |
| **wk_wt** | . . 157 | . . 157 | . . 157 | . . 157 | . . 157 | . . 157 | . . 0 |
| **wk_glucose** | . . 157 | . . 157 | . . 157 | . . 157 | . . 157 | . . 157 | . . 0 |
| **wk_ketone** | . . 157 | . . 157 | . . 157 | . . 157 | . . 157 | . . 157 | . . 0 |
| **wk_distance** | . . 157 | . . 157 | . . 157 | . . 157 | . . 157 | . . 157 | . . 0 |
| **wk_timeA** | . . 157 | . . 157 | . . 157 | . . 157 | . . 157 | . . 157 | . . 0 |
| **wk_timeX** | . . 157 | . . 157 | . . 157 | . . 157 | . . 157 | . . 157 | . . 0 |
| **ratioxa** | . . 0 | . . 0 | . . 0 | . . 0 | . . 0 | . . 0 | . . 0 |

| **7 Variables:** | wk_wt wk_glucose wk_ketone wk_distance wk_timeA wk_timeX ratioxa |
| --- | --- |

| **Simple Statistics** | | | | | | |
| --- | --- | --- | --- | --- | --- | --- |
| **Variable** | **N** | **Mean** | **Std Dev** | **Sum** | **Minimum** | **Maximum** |
| **wk_wt** | 118 | 336.14410 | 50.86861 | 39665 | 225.29000 | 438.00000 |
| **wk_glucose** | 120 | 98.62361 | 7.78422 | 11835 | 78.00000 | 118.00000 |
| **wk_ketone** | 120 | 0.59597 | 0.41910 | 71.51667 | 0.10000 | 1.50000 |
| **wk_distance** | 44 | 3.73516 | 0.63561 | 164.34716 | 2.23128 | 5.35762 |
| **wk_timeA** | 44 | 42.96742 | 18.24777 | 1891 | 17.20000 | 88.70000 |
| **wk_timeX** | 44 | 49.41061 | 21.47489 | 2174 | 10.60000 | 93.10000 |
| **ratioxa** | 44 | 1.30951 | 0.62257 | 57.61861 | 0.20229 | 2.81269 |

| **Pearson Correlation Coefficients Prob > |r| under H0: Rho=0 Number of Observations** | | | | | | | |
| --- | --- | --- | --- | --- | --- | --- | --- |
|  | **wk_wt** | **wk_glucose** | **wk_ketone** | **wk_distance** | **wk_timeA** | **wk_timeX** | **ratioxa** |
| **wk_wt** | 1.00000  118 | 0.32019 0.0005 116 | -0.19060 0.0404 116 | -0.01618 0.9170 44 | -0.13811 0.3831 42 | -0.15582 0.3244 42 | 0.00142 0.9929 42 |
| **wk_glucose** | 0.32019 0.0005 116 | 1.00000  120 | 0.15736 0.0861 120 | 0.22926 0.1344 44 | 0.25450 0.1038 42 | -0.20750 0.1873 42 | -0.41730 0.0060 42 |
| **wk_ketone** | -0.19060 0.0404 116 | 0.15736 0.0861 120 | 1.00000  120 | 0.43821 0.0029 44 | 0.29604 0.0570 42 | 0.62249 <.0001 42 | 0.17934 0.2558 42 |
| **wk_distance** | -0.01618 0.9170 44 | 0.22926 0.1344 44 | 0.43821 0.0029 44 | 1.00000  44 | 0.05389 0.8215 20 | -0.28060 0.2308 20 | -0.19983 0.3983 20 |
| **wk_timeA** | -0.13811 0.3831 42 | 0.25450 0.1038 42 | 0.29604 0.0570 42 | 0.05389 0.8215 20 | 1.00000  44 | 0.12452 0.4206 44 | -0.61751 <.0001 44 |
| **wk_timeX** | -0.15582 0.3244 42 | -0.20750 0.1873 42 | 0.62249 <.0001 42 | -0.28060 0.2308 20 | 0.12452 0.4206 44 | 1.00000  44 | 0.62271 <.0001 44 |
| **ratioxa** | 0.00142 0.9929 42 | -0.41730 0.0060 42 | 0.17934 0.2558 42 | -0.19983 0.3983 20 | -0.61751 <.0001 44 | 0.62271 <.0001 44 | 1.00000  44 |

| **7 Variables:** | wk_wt wk_glucose wk_ketone wk_distance wk_timeA wk_timeX ratioxa |
| --- | --- |

| **Simple Statistics** | | | | | | |
| --- | --- | --- | --- | --- | --- | --- |
| **Variable** | **N** | **Mean** | **Std Dev** | **Sum** | **Minimum** | **Maximum** |
| **wk_wt** | 122 | 322.58182 | 38.73708 | 39355 | 228.71571 | 410.00000 |
| **wk_glucose** | 136 | 98.40196 | 7.44402 | 13383 | 79.00000 | 121.50000 |
| **wk_ketone** | 136 | 0.90637 | 0.47345 | 123.26667 | 0.10000 | 2.10000 |
| **wk_distance** | 52 | 3.94795 | 0.73641 | 205.29330 | 1.84290 | 5.78317 |
| **wk_timeA** | 52 | 36.87244 | 14.16889 | 1917 | 14.10000 | 75.60000 |
| **wk_timeX** | 51 | 55.99673 | 18.81033 | 2856 | 27.80000 | 99.36667 |
| **ratioxa** | 51 | 1.69366 | 0.81714 | 86.37673 | 0.50926 | 4.11229 |

| **Pearson Correlation Coefficients Prob > |r| under H0: Rho=0 Number of Observations** | | | | | | | |
| --- | --- | --- | --- | --- | --- | --- | --- |
|  | **wk_wt** | **wk_glucose** | **wk_ketone** | **wk_distance** | **wk_timeA** | **wk_timeX** | **ratioxa** |
| **wk_wt** | 1.00000  122 | -0.07867 0.4183 108 | 0.07595 0.4346 108 | -0.11123 0.4324 52 | -0.34427 0.0343 38 | -0.01024 0.9520 37 | 0.34863 0.0345 37 |
| **wk_glucose** | -0.07867 0.4183 108 | 1.00000  136 | 0.05654 0.5132 136 | 0.11329 0.4239 52 | 0.16142 0.3329 38 | 0.05064 0.7660 37 | -0.07242 0.6701 37 |
| **wk_ketone** | 0.07595 0.4346 108 | 0.05654 0.5132 136 | 1.00000  136 | 0.33032 0.0168 52 | -0.04589 0.7844 38 | 0.56590 0.0003 37 | 0.46726 0.0035 37 |
| **wk_distance** | -0.11123 0.4324 52 | 0.11329 0.4239 52 | 0.33032 0.0168 52 | 1.00000  52 | 0.39712 0.2012 12 | 0.64914 0.0307 11 | -0.07401 0.8288 11 |
| **wk_timeA** | -0.34427 0.0343 38 | 0.16142 0.3329 38 | -0.04589 0.7844 38 | 0.39712 0.2012 12 | 1.00000  52 | 0.03370 0.8144 51 | -0.64574 <.0001 51 |
| **wk_timeX** | -0.01024 0.9520 37 | 0.05064 0.7660 37 | 0.56590 0.0003 37 | 0.64914 0.0307 11 | 0.03370 0.8144 51 | 1.00000  51 | 0.62380 <.0001 51 |
| **ratioxa** | 0.34863 0.0345 37 | -0.07242 0.6701 37 | 0.46726 0.0035 37 | -0.07401 0.8288 11 | -0.64574 <.0001 51 | 0.62380 <.0001 51 | 1.00000  51 |

| **Variable** | **Mean** | **Standard Deviation** | **Skewness** | **Kurtosis** | **Bimodality** |
| --- | --- | --- | --- | --- | --- |
| **wk_wt** | 0 | 1.0000 | -0.0649 | -0.6681 | 0.3964 |
| **wk_glucose** | 0 | 1.0000 | 0.3069 | 0.0650 | 0.3350 |
| **wk_ketone** | 0 | 1.0000 | -0.1842 | -1.4553 | 0.5921 |

| **Root-Mean-Square Total-Sample Standard Deviation** | 1 |
| --- | --- |

| **Cluster History** | | | | | | | | |
| --- | --- | --- | --- | --- | --- | --- | --- | --- |
| **Number of Clusters** | **Clusters Joined** | | **Freq** | **New Cluster RMS Std Dev** | **Semipartial R-Square** | **R-Square** | **Between Cluster Sum of Squares** | **Tie** |
| **47** | T24 | C7 | 2 | 0.0305 | 0.0000 | 1.00 | 0.0028 |  |
| **46** | T15 | C10 | 2 | 0.0452 | 0.0000 | 1.00 | 0.0061 |  |
| **45** | T8 | T26 | 2 | 0.0552 | 0.0001 | 1.00 | 0.0091 |  |
| **44** | C4 | C6 | 2 | 0.0731 | 0.0001 | 1.00 | 0.016 |  |
| **43** | T1 | C20 | 2 | 0.1146 | 0.0003 | .999 | 0.0394 |  |
| **42** | T14 | CL46 | 3 | 0.0932 | 0.0003 | .999 | 0.0459 |  |
| **41** | T21 | T22 | 2 | 0.1316 | 0.0004 | .999 | 0.0519 |  |
| **40** | CL44 | C5 | 3 | 0.1106 | 0.0004 | .998 | 0.0573 |  |
| **39** | T27 | C19 | 2 | 0.1394 | 0.0004 | .998 | 0.0583 |  |
| **38** | C11 | C12 | 2 | 0.1601 | 0.0005 | .997 | 0.0769 |  |
| **37** | C13 | C16 | 2 | 0.1728 | 0.0006 | .997 | 0.0895 |  |
| **36** | T12 | T23 | 2 | 0.1971 | 0.0008 | .996 | 0.1166 |  |
| **35** | CL39 | C17 | 3 | 0.1830 | 0.0010 | .995 | 0.1427 |  |
| **34** | T17 | CL47 | 3 | 0.1624 | 0.0011 | .994 | 0.1554 |  |
| **33** | T2 | C1 | 2 | 0.2289 | 0.0011 | .993 | 0.1572 |  |
| **32** | T4 | T7 | 2 | 0.2309 | 0.0011 | .992 | 0.16 |  |
| **31** | T10 | C21 | 2 | 0.2581 | 0.0014 | .990 | 0.1998 |  |
| **30** | C8 | CL38 | 3 | 0.2258 | 0.0016 | .989 | 0.2289 |  |
| **29** | T11 | CL34 | 4 | 0.2083 | 0.0016 | .987 | 0.2322 |  |
| **28** | C14 | C26 | 2 | 0.3087 | 0.0020 | .985 | 0.2858 |  |
| **27** | T3 | CL32 | 3 | 0.2733 | 0.0020 | .983 | 0.2882 |  |
| **26** | C15 | C18 | 2 | 0.3139 | 0.0021 | .981 | 0.2956 |  |
| **25** | CL43 | CL35 | 5 | 0.2302 | 0.0028 | .978 | 0.3954 |  |
| **24** | CL33 | C2 | 3 | 0.3052 | 0.0028 | .975 | 0.4017 |  |
| **23** | CL36 | CL41 | 4 | 0.2652 | 0.0033 | .972 | 0.4645 |  |
| **22** | CL45 | C25 | 3 | 0.2891 | 0.0035 | .968 | 0.4922 |  |
| **21** | CL37 | C22 | 3 | 0.3183 | 0.0037 | .965 | 0.5183 |  |
| **20** | CL29 | CL42 | 7 | 0.2388 | 0.0041 | .960 | 0.5842 |  |
| **19** | CL27 | T6 | 4 | 0.3576 | 0.0050 | .955 | 0.7028 |  |
| **18** | T5 | T9 | 2 | 0.4936 | 0.0052 | .950 | 0.7309 |  |
| **17** | CL22 | C24 | 4 | 0.3734 | 0.0053 | .945 | 0.7537 |  |
| **16** | CL31 | C3 | 3 | 0.4042 | 0.0055 | .939 | 0.7806 |  |
| **15** | CL21 | CL28 | 5 | 0.3919 | 0.0067 | .933 | 0.9492 |  |
| **14** | CL24 | CL40 | 6 | 0.3396 | 0.0078 | .925 | 1.0981 |  |
| **13** | CL23 | T13 | 5 | 0.3891 | 0.0084 | .917 | 1.184 |  |
| **12** | CL19 | CL18 | 6 | 0.4589 | 0.0091 | .907 | 1.2768 |  |
| **11** | CL26 | C23 | 3 | 0.5372 | 0.0102 | .897 | 1.4359 |  |
| **10** | CL30 | C9 | 4 | 0.4526 | 0.0109 | .886 | 1.5377 |  |
| **9** | CL25 | CL17 | 9 | 0.3939 | 0.0130 | .873 | 1.8334 |  |
| **8** | CL16 | CL15 | 8 | 0.5182 | 0.0200 | .853 | 2.8154 |  |
| **7** | CL20 | CL13 | 12 | 0.4552 | 0.0283 | .825 | 3.9938 |  |
| **6** | CL14 | CL8 | 14 | 0.5543 | 0.0327 | .792 | 4.6116 |  |
| **5** | CL9 | CL11 | 12 | 0.6094 | 0.0482 | .744 | 6.8007 |  |
| **4** | CL7 | CL10 | 16 | 0.5962 | 0.0519 | .692 | 7.3156 |  |
| **3** | CL6 | CL12 | 20 | 0.7934 | 0.1471 | .545 | 20.741 |  |
| **2** | CL5 | CL3 | 32 | 0.9067 | 0.2008 | .344 | 28.316 |  |
| **1** | CL2 | CL4 | 48 | 1.0000 | 0.3443 | .000 | 48.551 |  |

| **Obs** | **_NAME_** | **_PARENT_** | **_NCL_** | **_FREQ_** | **_HEIGHT_** | **_RMSSTD_** | **_SPRSQ_** | **_RSQ_** | **_PSF_** | **_PST2_** |
| --- | --- | --- | --- | --- | --- | --- | --- | --- | --- | --- |
| **1** | T24 | CL47 | 48 | 1 | 0.000000 | 0.000000 | 0 | 1.00000 | . | . |
| **2** | C7 | CL47 | 48 | 1 | 0.000000 | 0.000000 | 0 | 1.00000 | . | . |
| **3** | T15 | CL46 | 48 | 1 | 0.000000 | 0.000000 | 0 | 1.00000 | . | . |
| **4** | C10 | CL46 | 48 | 1 | 0.000000 | 0.000000 | 0 | 1.00000 | . | . |
| **5** | T8 | CL45 | 48 | 1 | 0.000000 | 0.000000 | 0 | 1.00000 | . | . |
| **6** | T26 | CL45 | 48 | 1 | 0.000000 | 0.000000 | 0 | 1.00000 | . | . |
| **7** | C4 | CL44 | 48 | 1 | 0.000000 | 0.000000 | 0 | 1.00000 | . | . |
| **8** | C6 | CL44 | 48 | 1 | 0.000000 | 0.000000 | 0 | 1.00000 | . | . |
| **9** | T1 | CL43 | 48 | 1 | 0.000000 | 0.000000 | 0 | 1.00000 | . | . |
| **10** | C20 | CL43 | 48 | 1 | 0.000000 | 0.000000 | 0 | 1.00000 | . | . |
| **11** | T14 | CL42 | 48 | 1 | 0.000000 | 0.000000 | 0 | 1.00000 | . | . |
| **12** | CL46 | CL42 | 46 | 2 | 0.006124 | 0.045182 | .000043434 | 0.99994 | 703.197 | . |
| **13** | T21 | CL41 | 48 | 1 | 0.000000 | 0.000000 | 0 | 1.00000 | . | . |
| **14** | T22 | CL41 | 48 | 1 | 0.000000 | 0.000000 | 0 | 1.00000 | . | . |
| **15** | CL44 | CL40 | 44 | 2 | 0.016021 | 0.073079 | .000113627 | 0.99976 | 384.945 | . |
| **16** | C5 | CL40 | 48 | 1 | 0.000000 | 0.000000 | 0 | 1.00000 | . | . |
| **17** | T27 | CL39 | 48 | 1 | 0.000000 | 0.000000 | 0 | 1.00000 | . | . |
| **18** | C19 | CL39 | 48 | 1 | 0.000000 | 0.000000 | 0 | 1.00000 | . | . |
| **19** | C11 | CL38 | 48 | 1 | 0.000000 | 0.000000 | 0 | 1.00000 | . | . |
| **20** | C12 | CL38 | 48 | 1 | 0.000000 | 0.000000 | 0 | 1.00000 | . | . |

| **Obs** | **_ERSQ_** | **_RATIO_** | **_LOGR_** | **_CCC_** | **wk_wt** | **wk_glucose** | **wk_ketone** | **_DIST_** | **_AVLINK_** | **ID** |
| --- | --- | --- | --- | --- | --- | --- | --- | --- | --- | --- |
| **1** | . | . | . | . | -0.40663 | -1.14379 | -1.30968 | 0.00000 | 0.00000 | T24 |
| **2** | . | . | . | . | -0.44228 | -1.07820 | -1.30968 | 0.00000 | 0.00000 | C7 |
| **3** | . | . | . | . | -0.65622 | -0.94701 | -1.30968 | 0.00000 | 0.00000 | T15 |
| **4** | . | . | . | . | -0.74536 | -0.88141 | -1.30968 | 0.00000 | 0.00000 | C10 |
| **5** | . | . | . | . | 1.72954 | 0.16808 | 0.81152 | 0.00000 | 0.00000 | T8 |
| **6** | . | . | . | . | 1.69707 | 0.29927 | 0.81152 | 0.00000 | 0.00000 | T26 |
| **7** | . | . | . | . | -0.65614 | 0.69283 | 0.13659 | 0.00000 | 0.00000 | C4 |
| **8** | . | . | . | . | -0.53435 | 0.82402 | 0.13659 | 0.00000 | 0.00000 | C6 |
| **9** | . | . | . | . | 1.32253 | -1.07820 | 0.52227 | 0.00000 | 0.00000 | T1 |
| **10** | . | . | . | . | 1.26920 | -0.88141 | 0.71510 | 0.00000 | 0.00000 | C20 |
| **11** | . | . | . | . | -0.90581 | -0.75023 | -1.30968 | 0.00000 | 0.00000 | T14 |
| **12** | . | . | . | . | -0.70079 | -0.91421 | -1.30968 | 0.11067 | 0.11067 |  |
| **13** | . | . | . | . | -0.21052 | -0.42226 | -1.30968 | 0.00000 | 0.00000 | T21 |
| **14** | . | . | . | . | -0.05007 | -0.15988 | -1.21326 | 0.00000 | 0.00000 | T22 |
| **15** | . | . | . | . | -0.59525 | 0.75843 | 0.13659 | 0.17901 | 0.17901 |  |
| **16** | . | . | . | . | -0.37787 | 0.56165 | 0.13659 | 0.00000 | 0.00000 | C5 |
| **17** | . | . | . | . | 1.26920 | -0.42226 | 0.71510 | 0.00000 | 0.00000 | T27 |
| **18** | . | . | . | . | 0.94829 | -0.35667 | 0.81152 | 0.00000 | 0.00000 | C19 |
| **19** | . | . | . | . | -1.35150 | 0.03690 | -1.30968 | 0.00000 | 0.00000 | C11 |
| **20** | . | . | . | . | -1.31585 | 0.29927 | -1.02043 | 0.00000 | 0.00000 | C12 |

| **Obs** | **_NAME_** | **wk_wt** | **wk_glucose** | **wk_ketone** | **CLUSTER** |
| --- | --- | --- | --- | --- | --- |
| **1** | C1 | -1.30704 | 1.08639 | 0.32943 | 3 |
| **2** | C10 | -0.74536 | -0.88141 | -1.30968 | 1 |
| **3** | C11 | -1.35150 | 0.03690 | -1.30968 | 1 |
| **4** | C12 | -1.31585 | 0.29927 | -1.02043 | 1 |
| **5** | C13 | 0.03907 | 0.10249 | 0.90794 | 3 |
| **6** | C14 | -0.44228 | 0.16808 | 1.39003 | 3 |
| **7** | C15 | 0.09255 | -0.94701 | 1.39003 | 2 |
| **8** | C16 | 0.30649 | 0.43046 | 0.90794 | 3 |
| **9** | C17 | 0.94829 | -0.75023 | 1.00436 | 2 |
| **10** | C18 | -0.12138 | -1.40616 | 0.81152 | 2 |
| **11** | C19 | 0.94829 | -0.35667 | 0.81152 | 2 |
| **12** | C2 | -1.40056 | 1.08639 | 0.90794 | 3 |
| **13** | C20 | 1.26920 | -0.88141 | 0.71510 | 2 |
| **14** | C21 | -0.70970 | -0.48785 | 0.71510 | 3 |
| **15** | C22 | 0.19952 | -0.15988 | 1.67929 | 3 |
| **16** | C23 | 0.41346 | -2.45566 | 0.52227 | 2 |
| **17** | C24 | 1.69707 | -0.15988 | 1.58287 | 2 |
| **18** | C25 | 0.94829 | 0.16808 | 0.42585 | 2 |
| **19** | C26 | -0.92364 | 0.49605 | 0.90794 | 3 |
| **20** | C3 | -1.66079 | -0.48785 | 1.19719 | 3 |
| **21** | C4 | -0.65614 | 0.69283 | 0.13659 | 3 |
| **22** | C5 | -0.37787 | 0.56165 | 0.13659 | 3 |
| **23** | C6 | -0.53435 | 0.82402 | 0.13659 | 3 |
| **24** | C7 | -0.44228 | -1.07820 | -1.30968 | 1 |
| **25** | C8 | -0.87015 | 0.49605 | -1.30968 | 1 |
| **26** | C9 | -2.38552 | -0.48785 | -1.30968 | 1 |
| **27** | T1 | 1.32253 | -1.07820 | 0.52227 | 2 |
| **28** | T10 | -1.02342 | -0.22548 | 0.23301 | 3 |
| **29** | T11 | -0.12138 | -0.88141 | -0.92401 | 1 |
| **30** | T12 | 0.69870 | -0.61904 | -1.11685 | 1 |
| **31** | T13 | 1.09092 | 0.43046 | -1.30968 | 1 |
| **32** | T14 | -0.90581 | -0.75023 | -1.30968 | 1 |
| **33** | T15 | -0.65622 | -0.94701 | -1.30968 | 1 |
| **34** | T17 | -0.10355 | -1.47176 | -1.30968 | 1 |
| **35** | T2 | -1.16622 | 0.62724 | 0.04017 | 3 |
| **36** | T21 | -0.21052 | -0.42226 | -1.30968 | 1 |
| **37** | T22 | -0.05007 | -0.15988 | -1.21326 | 1 |
| **38** | T23 | 0.34215 | -0.35667 | -1.30968 | 1 |
| **39** | T24 | -0.40663 | -1.14379 | -1.30968 | 1 |
| **40** | T26 | 1.69707 | 0.29927 | 0.81152 | 2 |
| **41** | T27 | 1.26920 | -0.42226 | 0.71510 | 2 |
| **42** | T3 | 0.53899 | 1.54555 | -0.05624 | 4 |
| **43** | T4 | 0.32903 | 2.07030 | 0.42585 | 4 |
| **44** | T5 | 1.51323 | 1.54555 | -0.24908 | 4 |
| **45** | T6 | 0.58545 | 2.00471 | -0.82759 | 4 |
| **46** | T7 | 0.72152 | 2.20149 | 0.04017 | 4 |
| **47** | T8 | 1.72954 | 0.16808 | 0.81152 | 2 |
| **48** | T9 | 1.18767 | 1.67674 | 0.90794 | 4 |

| **Obs** | **CLUSTER** | **_TYPE_** | **_FREQ_** | **_STAT_** | **wk_wt** | **wk_glucose** | **wk_ketone** |
| --- | --- | --- | --- | --- | --- | --- | --- |
| **1** | 1 | 0 | 16 | MEAN | -0.46457 | -0.49605 | -1.24942 |
| **2** | 2 | 0 | 12 | MEAN | 1.01784 | -0.65184 | 0.84366 |
| **3** | 3 | 0 | 14 | MEAN | -0.68978 | 0.33675 | 0.68755 |
| **4** | 4 | 0 | 6 | MEAN | 0.81265 | 1.84072 | 0.04017 |

| **Total Sample Size** | 48 | **DF Total** | 47 |
| --- | --- | --- | --- |
| **Variables** | 3 | **DF Within Classes** | 46 |
| **Classes** | 2 | **DF Between Classes** | 1 |

| **Number of Observations Read** | 48 |
| --- | --- |
| **Number of Observations Used** | 48 |

| **Class Level Information** | | | | | |
| --- | --- | --- | --- | --- | --- |
| **Group** | **Variable Name** | **Frequency** | **Weight** | **Proportion** | **Prior Probability** |
| **HFD** | HFD | 22 | 22.0000 | 0.458333 | 0.500000 |
| **SD** | SD | 26 | 26.0000 | 0.541667 | 0.500000 |

| **Within Covariance Matrix Information** | | |
| --- | --- | --- |
| **Group** | **Covariance Matrix Rank** | **Natural Log of the Determinant of the Covariance Matrix** |
| **HFD** | 3 | -0.75991 |
| **SD** | 3 | -0.87626 |
| **Pooled** | 3 | -0.62428 |

| **Chi-Square** | **DF** | **Pr > ChiSq** |
| --- | --- | --- |
| 8.494878 | 6 | 0.2040 |

| ***Since the Chi-Square value is not significant at the 0.1 level, a pooled covariance matrix will be used in the discriminant function. Reference: Morrison, D.F. (1976) Multivariate Statistical Methods p252.*** |
| --- |

| **Generalized Squared Distance to Group** | | |
| --- | --- | --- |
| **From Group** | **HFD** | **SD** |
| **HFD** | 0 | 2.95895 |
| **SD** | 2.95895 | 0 |

| **Linear Discriminant Function for Group** | | |
| --- | --- | --- |
| **Variable** | **HFD** | **SD** |
| **Constant** | -0.43408 | -0.31079 |
| **wk_wt** | 0.95663 | -0.80945 |
| **wk_glucose** | 0.37428 | -0.31670 |
| **wk_ketone** | -1.05553 | 0.89314 |

| **Number of Observations and Percent Classified into Group** | | | |
| --- | --- | --- | --- |
| **From Group** | **HFD** | **SD** | **Total** |
| **HFD** | 20 90.91 | 2 9.09 | 22 100.00 |
| **SD** | 6 23.08 | 20 76.92 | 26 100.00 |
| **Total** | 26 54.17 | 22 45.83 | 48 100.00 |
| **Priors** | 0.5 | 0.5 |  |

| **Error Count Estimates for Group** | | | |
| --- | --- | --- | --- |
|  | **HFD** | **SD** | **Total** |
| **Rate** | 0.0909 | 0.2308 | 0.1608 |
| **Priors** | 0.5000 | 0.5000 |  |

| **Posterior Probability of Membership in Group** | | | | | |
| --- | --- | --- | --- | --- | --- |
| **Obs** | **From Group** | **Classified into Group** | | **HFD** | **SD** |
| **1** | **HFD** | **HFD** |  | 0.5469 | 0.4531 |
| **2** | **HFD** | **SD** | * | 0.0869 | 0.9131 |
| **3** | **HFD** | **HFD** |  | 0.8693 | 0.1307 |
| **4** | **HFD** | **HFD** |  | 0.6867 | 0.3133 |
| **5** | **HFD** | **HFD** |  | 0.9844 | 0.0156 |
| **6** | **HFD** | **HFD** |  | 0.9807 | 0.0193 |
| **7** | **HFD** | **HFD** |  | 0.9203 | 0.0797 |
| **8** | **HFD** | **HFD** |  | 0.7868 | 0.2132 |
| **9** | **HFD** | **HFD** |  | 0.7574 | 0.2426 |
| **10** | **HFD** | **SD** | * | 0.0398 | 0.9602 |
| **11** | **HFD** | **HFD** |  | 0.6792 | 0.3208 |
| **12** | **HFD** | **HFD** |  | 0.9422 | 0.0578 |
| **13** | **HFD** | **HFD** |  | 0.9915 | 0.0085 |
| **14** | **HFD** | **HFD** |  | 0.5272 | 0.4728 |
| **15** | **HFD** | **HFD** |  | 0.6070 | 0.3930 |
| **16** | **HFD** | **HFD** |  | 0.7349 | 0.2651 |
| **17** | **HFD** | **HFD** |  | 0.8428 | 0.1572 |
| **18** | **HFD** | **HFD** |  | 0.8780 | 0.1220 |
| **19** | **HFD** | **HFD** |  | 0.9382 | 0.0618 |
| **20** | **HFD** | **HFD** |  | 0.6775 | 0.3225 |
| **21** | **HFD** | **HFD** |  | 0.7934 | 0.2066 |
| **22** | **HFD** | **HFD** |  | 0.5654 | 0.4346 |
| **23** | **SD** | **SD** |  | 0.0982 | 0.9018 |
| **24** | **SD** | **SD** |  | 0.0259 | 0.9741 |
| **25** | **SD** | **SD** |  | 0.0020 | 0.9980 |
| **26** | **SD** | **SD** |  | 0.2687 | 0.7313 |
| **27** | **SD** | **SD** |  | 0.3496 | 0.6504 |
| **28** | **SD** | **SD** |  | 0.3349 | 0.6651 |
| **29** | **SD** | **HFD** | * | 0.7767 | 0.2233 |
| **30** | **SD** | **HFD** | * | 0.8347 | 0.1653 |
| **31** | **SD** | **SD** |  | 0.1274 | 0.8726 |
| **32** | **SD** | **HFD** | * | 0.6831 | 0.3169 |
| **33** | **SD** | **HFD** | * | 0.5792 | 0.4208 |
| **34** | **SD** | **SD** |  | 0.4846 | 0.5154 |
| **35** | **SD** | **SD** |  | 0.1542 | 0.8458 |
| **36** | **SD** | **SD** |  | 0.0300 | 0.9700 |
| **37** | **SD** | **SD** |  | 0.0358 | 0.9642 |
| **38** | **SD** | **SD** |  | 0.2704 | 0.7296 |
| **39** | **SD** | **SD** |  | 0.3132 | 0.6868 |
| **40** | **SD** | **SD** |  | 0.0557 | 0.9443 |
| **41** | **SD** | **SD** |  | 0.4612 | 0.5388 |
| **42** | **SD** | **HFD** | * | 0.5927 | 0.4073 |
| **43** | **SD** | **SD** |  | 0.0445 | 0.9555 |
| **44** | **SD** | **SD** |  | 0.0426 | 0.9574 |
| **45** | **SD** | **SD** |  | 0.1321 | 0.8679 |
| **46** | **SD** | **HFD** | * | 0.5023 | 0.4977 |
| **47** | **SD** | **HFD** | * | 0.7315 | 0.2685 |
| **48** | **SD** | **SD** |  | 0.0414 | 0.9586 |

| **** Misclassified observation*** |
| --- |

| **Number of Observations and Percent Classified into Group** | | | |
| --- | --- | --- | --- |
| **From Group** | **HFD** | **SD** | **Total** |
| **HFD** | 20 90.91 | 2 9.09 | 22 100.00 |
| **SD** | 7 26.92 | 19 73.08 | 26 100.00 |
| **Total** | 27 56.25 | 21 43.75 | 48 100.00 |
| **Priors** | 0.5 | 0.5 |  |

| **Error Count Estimates for Group** | | | |
| --- | --- | --- | --- |
|  | **HFD** | **SD** | **Total** |
| **Rate** | 0.0909 | 0.2692 | 0.1801 |
| **Priors** | 0.5000 | 0.5000 |  |

**Group=HFD res=Hit**

| **Variable** | **N** | **Mean** | **Std Dev** | **Minimum** | **Maximum** |
| --- | --- | --- | --- | --- | --- |
| wk_wt wk_glucose wk_ketone | 20 20 20 | 338.1526905 99.1250000 0.5375000 | 14.7906772 9.2748599 0.4622243 | 311.3333333 86.5000000 0.1000000 | 360.6071429 114.5000000 1.2500000 |

**Group=HFD res=Miss**

| **Variable** | **N** | **Mean** | **Std Dev** | **Minimum** | **Maximum** |
| --- | --- | --- | --- | --- | --- |
| wk_wt wk_glucose wk_ketone | 2 2 2 | 307.7992857 99.2500000 0.8500000 | 1.8879751 4.5961941 0.0707107 | 306.4642857 96.0000000 0.8000000 | 309.1342857 102.5000000 0.9000000 |

**Group=SD res=Hit**

| **Variable** | **N** | **Mean** | **Std Dev** | **Minimum** | **Maximum** |
| --- | --- | --- | --- | --- | --- |
| wk_wt wk_glucose wk_ketone | 19 19 19 | 319.5236341 97.0000000 1.0947368 | 16.6257578 6.8556546 0.3943682 | 283.6666667 79.0000000 0.1000000 | 346.0000000 106.0000000 1.6500000 |

**Group=SD res=Miss**

| **Variable** | **N** | **Mean** | **Std Dev** | **Minimum** | **Maximum** |
| --- | --- | --- | --- | --- | --- |
| wk_wt wk_glucose wk_ketone | 7 7 7 | 329.6190476 95.2142857 0.5928571 | 22.4997355 4.6802523 0.6405912 | 303.0000000 89.5000000 0.1000000 | 360.0000000 101.5000000 1.6000000 |

| **Observations** | 48 |
| --- | --- |
| **Variables** | 3 |

| **Simple Statistics** | | | |
| --- | --- | --- | --- |
|  | **wk_wt** | **wk_glucose** | **wk_ketone** |
| **Mean** | 328.2694742 | 97.71875000 | 0.7791666667 |
| **StD** | 18.6972901 | 7.62268887 | 0.5185734661 |

| **Correlation Matrix** | | | |
| --- | --- | --- | --- |
|  | **wk_wt** | **wk_glucose** | **wk_ketone** |
| **wk_wt** | 1.0000 | 0.0823 | 0.3114 |
| **wk_glucose** | 0.0823 | 1.0000 | 0.1246 |
| **wk_ketone** | 0.3114 | 0.1246 | 1.0000 |

| **Eigenvalues of the Correlation Matrix** | | | | |
| --- | --- | --- | --- | --- |
|  | **Eigenvalue** | **Difference** | **Proportion** | **Cumulative** |
| **1** | 1.36952336 | 0.42446275 | 0.4565 | 0.4565 |
| **2** | 0.94506061 | 0.25964458 | 0.3150 | 0.7715 |
| **3** | 0.68541603 |  | 0.2285 | 1.0000 |

| **Eigenvectors** | | | |
| --- | --- | --- | --- |
|  | **Prin1** | **Prin2** | **Prin3** |
| **wk_wt** | 0.645600 | -.336774 | 0.685407 |
| **wk_glucose** | 0.369183 | 0.923301 | 0.105922 |
| **wk_ketone** | 0.668509 | -.184657 | -.720415 |

| **Number of Observations Read** | 157 |
| --- | --- |
| **Number of Observations Used** | 157 |

| **Analysis of Variance** | | | | | |
| --- | --- | --- | --- | --- | --- |
| **Source** | **DF** | **Sum of Squares** | **Mean Square** | **F Value** | **Pr > F** |
| **Model** | 0 | 0 | . | . | . |
| **Error** | 156 | 0 | 0 |  |  |
| **Corrected Total** | 156 | 0 |  |  |  |

| **Root MSE** | 0 | **R-Square** | . |
| --- | --- | --- | --- |
| **Dependent Mean** | 0 | **Adj R-Sq** | . |
| **Coeff Var** | . |  |  |

| **Note:** | Model is not full rank. Least-squares solutions for the parameters are not unique. Some statistics will be misleading. A reported DF of 0 or B means that the estimate is biased. |
| --- | --- |

| **Note:** | The following parameters have been set to 0, since the variables are a linear combination of other variables as shown. |
| --- | --- |

| **Week =** | 0 |
| --- | --- |

| **Parameter Estimates** | | | | | |
| --- | --- | --- | --- | --- | --- |
| **Variable** | **DF** | **Parameter Estimate** | **Standard Error** | **t Value** | **Pr > |t|** |
| **Intercept** | 1 | 0 | 0 | . | . |
| **Week** | 0 | 0 | . | . | . |

| **Number of Observations Read** | 157 |
| --- | --- |
| **Number of Observations Used** | 157 |

| **Analysis of Variance** | | | | | |
| --- | --- | --- | --- | --- | --- |
| **Source** | **DF** | **Sum of Squares** | **Mean Square** | **F Value** | **Pr > F** |
| **Model** | 0 | 0 | . | . | . |
| **Error** | 156 | 0 | 0 |  |  |
| **Corrected Total** | 156 | 0 |  |  |  |

| **Root MSE** | 0 | **R-Square** | . |
| --- | --- | --- | --- |
| **Dependent Mean** | 0 | **Adj R-Sq** | . |
| **Coeff Var** | . |  |  |

| **Note:** | Model is not full rank. Least-squares solutions for the parameters are not unique. Some statistics will be misleading. A reported DF of 0 or B means that the estimate is biased. |
| --- | --- |

| **Note:** | The following parameters have been set to 0, since the variables are a linear combination of other variables as shown. |
| --- | --- |

| **Week =** | 0 |
| --- | --- |
| **w2 =** | 0 |

| **Parameter Estimates** | | | | | |
| --- | --- | --- | --- | --- | --- |
| **Variable** | **DF** | **Parameter Estimate** | **Standard Error** | **t Value** | **Pr > |t|** |
| **Intercept** | 1 | 0 | 0 | . | . |
| **Week** | 0 | 0 | . | . | . |
| **w2** | 0 | 0 | . | . | . |

| **Number of Observations Read** | 132 |
| --- | --- |
| **Number of Observations Used** | 120 |
| **Number of Observations with Missing Values** | 12 |

| **Analysis of Variance** | | | | | |
| --- | --- | --- | --- | --- | --- |
| **Source** | **DF** | **Sum of Squares** | **Mean Square** | **F Value** | **Pr > F** |
| **Model** | 1 | 571.05501 | 571.05501 | 10.15 | 0.0018 |
| **Error** | 118 | 6639.63920 | 56.26813 |  |  |
| **Corrected Total** | 119 | 7210.69421 |  |  |  |

| **Root MSE** | 7.50121 | **R-Square** | 0.0792 |
| --- | --- | --- | --- |
| **Dependent Mean** | 98.62361 | **Adj R-Sq** | 0.0714 |
| **Coeff Var** | 7.60590 |  |  |

| **Parameter Estimates** | | | | | |
| --- | --- | --- | --- | --- | --- |
| **Variable** | **DF** | **Parameter Estimate** | **Standard Error** | **t Value** | **Pr > |t|** |
| **Intercept** | 1 | 94.15822 | 1.56001 | 60.36 | <.0001 |
| **Week** | 1 | 1.37397 | 0.43129 | 3.19 | 0.0018 |

| **Number of Observations Read** | 132 |
| --- | --- |
| **Number of Observations Used** | 120 |
| **Number of Observations with Missing Values** | 12 |

| **Analysis of Variance** | | | | | |
| --- | --- | --- | --- | --- | --- |
| **Source** | **DF** | **Sum of Squares** | **Mean Square** | **F Value** | **Pr > F** |
| **Model** | 2 | 630.64602 | 315.32301 | 5.61 | 0.0047 |
| **Error** | 117 | 6580.04819 | 56.23973 |  |  |
| **Corrected Total** | 119 | 7210.69421 |  |  |  |

| **Root MSE** | 7.49932 | **R-Square** | 0.0875 |
| --- | --- | --- | --- |
| **Dependent Mean** | 98.62361 | **Adj R-Sq** | 0.0719 |
| **Coeff Var** | 7.60398 |  |  |

| **Parameter Estimates** | | | | | |
| --- | --- | --- | --- | --- | --- |
| **Variable** | **DF** | **Parameter Estimate** | **Standard Error** | **t Value** | **Pr > |t|** |
| **Intercept** | 1 | 91.55829 | 2.96848 | 30.84 | <.0001 |
| **Week** | 1 | 3.40748 | 2.02202 | 1.69 | 0.0946 |
| **w2** | 1 | -0.30642 | 0.29768 | -1.03 | 0.3054 |

| **Number of Observations Read** | 156 |
| --- | --- |
| **Number of Observations Used** | 136 |
| **Number of Observations with Missing Values** | 20 |

| **Analysis of Variance** | | | | | |
| --- | --- | --- | --- | --- | --- |
| **Source** | **DF** | **Sum of Squares** | **Mean Square** | **F Value** | **Pr > F** |
| **Model** | 1 | 173.57222 | 173.57222 | 3.18 | 0.0767 |
| **Error** | 134 | 7307.23170 | 54.53158 |  |  |
| **Corrected Total** | 135 | 7480.80392 |  |  |  |

| **Root MSE** | 7.38455 | **R-Square** | 0.0232 |
| --- | --- | --- | --- |
| **Dependent Mean** | 98.40196 | **Adj R-Sq** | 0.0159 |
| **Coeff Var** | 7.50447 |  |  |

| **Parameter Estimates** | | | | | |
| --- | --- | --- | --- | --- | --- |
| **Variable** | **DF** | **Parameter Estimate** | **Standard Error** | **t Value** | **Pr > |t|** |
| **Intercept** | 1 | 100.73972 | 1.45532 | 69.22 | <.0001 |
| **Week** | 1 | -0.74633 | 0.41832 | -1.78 | 0.0767 |

| **Number of Observations Read** | 156 |
| --- | --- |
| **Number of Observations Used** | 136 |
| **Number of Observations with Missing Values** | 20 |

| **Analysis of Variance** | | | | | |
| --- | --- | --- | --- | --- | --- |
| **Source** | **DF** | **Sum of Squares** | **Mean Square** | **F Value** | **Pr > F** |
| **Model** | 2 | 341.83947 | 170.91974 | 3.18 | 0.0446 |
| **Error** | 133 | 7138.96445 | 53.67642 |  |  |
| **Corrected Total** | 135 | 7480.80392 |  |  |  |

| **Root MSE** | 7.32642 | **R-Square** | 0.0457 |
| --- | --- | --- | --- |
| **Dependent Mean** | 98.40196 | **Adj R-Sq** | 0.0313 |
| **Coeff Var** | 7.44540 |  |  |

| **Parameter Estimates** | | | | | |
| --- | --- | --- | --- | --- | --- |
| **Variable** | **DF** | **Parameter Estimate** | **Standard Error** | **t Value** | **Pr > |t|** |
| **Intercept** | 1 | 104.92962 | 2.77214 | 37.85 | <.0001 |
| **Week** | 1 | -4.13273 | 1.95714 | -2.11 | 0.0366 |
| **w2** | 1 | 0.53024 | 0.29948 | 1.77 | 0.0789 |

| **Model Information** | |
| --- | --- |
| **Data Set** | WORK.WEEKLY2 |
| **Dependent Variable** | wk_glucose |
| **Covariance Structure** | Autoregressive |
| **Subject Effect** | ID(Group) |
| **Estimation Method** | REML |
| **Residual Variance Method** | Profile |
| **Fixed Effects SE Method** | Kenward-Roger |
| **Degrees of Freedom Method** | Kenward-Roger |

| **Class Level Information** | | |
| --- | --- | --- |
| **Class** | **Levels** | **Values** |
| **Group** | 3 | 0 HFD SD |
| **ID** | 49 | 0 C1 C10 C11 C12 C13 C14 C15 C16 C17 C18 C19 C2 C20 C21 C22 C23 C24 C25 C26 C3 C4 C5 C6 C7 C8 C9 T1 T10 T11 T12 T13 T14 T15 T17 T2 T21 T22 T23 T24 T26 T27 T3 T4 T5 T6 T7 T8 T9 |

| **Dimensions** | |
| --- | --- |
| **Covariance Parameters** | 2 |
| **Columns in X** | 8 |
| **Columns in Z** | 0 |
| **Subjects** | 49 |
| **Max Obs per Subject** | 157 |

| **Number of Observations** | |
| --- | --- |
| **Number of Observations Read** | 445 |
| **Number of Observations Used** | 413 |
| **Number of Observations Not Used** | 32 |

| **Iteration History** | | | |
| --- | --- | --- | --- |
| **Iteration** | **Evaluations** | **-2 Res Log Like** | **Criterion** |
| **0** | 1 | 2625.01535194 |  |
| **1** | 2 | 2608.12360230 | 0.00000019 |
| **2** | 1 | 2608.12342269 | 0.00000000 |

| Convergence criteria met. |
| --- |

| **Covariance Parameter Estimates** | | |
| --- | --- | --- |
| **Cov Parm** | **Subject** | **Estimate** |
| **AR(1)** | ID(Group) | 0.2462 |
| **Residual** |  | 34.8172 |

| **Fit Statistics** | |
| --- | --- |
| **-2 Res Log Likelihood** | 2608.1 |
| **AIC (Smaller is Better)** | 2612.1 |
| **AICC (Smaller is Better)** | 2612.2 |
| **BIC (Smaller is Better)** | 2615.9 |

| **Null Model Likelihood Ratio Test** | | |
| --- | --- | --- |
| **DF** | **Chi-Square** | **Pr > ChiSq** |
| 1 | 16.89 | <.0001 |

| **Solution for Fixed Effects** | | | | | | |
| --- | --- | --- | --- | --- | --- | --- |
| **Effect** | **Group** | **Estimate** | **Standard Error** | **DF** | **t Value** | **Pr > |t|** |
| **Intercept** |  | 100.55 | 1.2924 | 209 | 77.80 | <.0001 |
| **Group** | 0 | -100.55 | 1.4251 | 169 | -70.56 | <.0001 |
| **Group** | HFD | -6.5130 | 1.8851 | 204 | -3.46 | 0.0007 |
| **Group** | SD | 0 | . | . | . | . |
| **Week** |  | -0.6523 | 0.3637 | 255 | -1.79 | 0.0741 |
| **Week*Group** | 0 | 0 | . | . | . | . |
| **Week*Group** | HFD | 2.0716 | 0.5200 | 248 | 3.98 | <.0001 |
| **Week*Group** | SD | 0 | . | . | . | . |

| **Type 3 Tests of Fixed Effects** | | | | |
| --- | --- | --- | --- | --- |
| **Effect** | **Num DF** | **Den DF** | **F Value** | **Pr > F** |
| **Group** | 2 | 171 | 3820.31 | <.0001 |
| **Week** | 1 | 248 | 2.18 | 0.1415 |
| **Week*Group** | 1 | 248 | 15.87 | <.0001 |

| **Least Squares Means** | | | | | | | |
| --- | --- | --- | --- | --- | --- | --- | --- |
| **Effect** | **Group** | **Week** | **Estimate** | **Standard Error** | **DF** | **t Value** | **Pr > |t|** |
| **Group** | 0 | 1.00 | Non-est | . | . | . | . |
| **Group** | HFD | 1.00 | 95.4519 | 1.0602 | 174 | 90.04 | <.0001 |
| **Group** | SD | 1.00 | 99.8932 | 0.9872 | 180 | 101.19 | <.0001 |
| **Group** | 0 | 2.00 | Non-est | . | . | . | . |
| **Group** | HFD | 2.00 | 96.8713 | 0.8006 | 137 | 120.99 | <.0001 |
| **Group** | SD | 2.00 | 99.2410 | 0.7371 | 137 | 134.64 | <.0001 |
| **Group** | 0 | 3.00 | Non-est | . | . | . | . |
| **Group** | HFD | 3.00 | 98.2906 | 0.6591 | 106 | 149.12 | <.0001 |
| **Group** | SD | 3.00 | 98.5887 | 0.6136 | 106 | 160.68 | <.0001 |
| **Group** | 0 | 4.00 | Non-est | . | . | . | . |
| **Group** | HFD | 4.00 | 99.7100 | 0.7101 | 117 | 140.42 | <.0001 |
| **Group** | SD | 4.00 | 97.9364 | 0.6886 | 125 | 142.22 | <.0001 |
| **Group** | 0 | 5.00 | Non-est | . | . | . | . |
| **Group** | HFD | 5.00 | 101.13 | 0.9221 | 155 | 109.67 | <.0001 |
| **Group** | SD | 5.00 | 97.2842 | 0.9146 | 169 | 106.37 | <.0001 |
| **Group** | 0 | 6.00 | Non-est | . | . | . | . |
| **Group** | HFD | 6.00 | 102.55 | 1.2135 | 186 | 84.50 | <.0001 |
| **Group** | SD | 6.00 | 96.6319 | 1.2097 | 201 | 79.88 | <.0001 |

| **Differences of Least Squares Means** | | | | | | | | |
| --- | --- | --- | --- | --- | --- | --- | --- | --- |
| **Effect** | **Group** | **_Group** | **Week** | **Estimate** | **Standard Error** | **DF** | **t Value** | **Pr > |t|** |
| **Group** | 0 | HFD | 1.00 | Non-est | . | . | . | . |
| **Group** | 0 | SD | 1.00 | Non-est | . | . | . | . |
| **Group** | HFD | SD | 1.00 | -4.4414 | 1.4486 | 177 | -3.07 | 0.0025 |
| **Group** | 0 | HFD | 2.00 | Non-est | . | . | . | . |
| **Group** | 0 | SD | 2.00 | Non-est | . | . | . | . |
| **Group** | HFD | SD | 2.00 | -2.3697 | 1.0883 | 137 | -2.18 | 0.0312 |
| **Group** | 0 | HFD | 3.00 | Non-est | . | . | . | . |
| **Group** | 0 | SD | 3.00 | Non-est | . | . | . | . |
| **Group** | HFD | SD | 3.00 | -0.2981 | 0.9005 | 106 | -0.33 | 0.7413 |
| **Group** | 0 | HFD | 4.00 | Non-est | . | . | . | . |
| **Group** | 0 | SD | 4.00 | Non-est | . | . | . | . |
| **Group** | HFD | SD | 4.00 | 1.7735 | 0.9892 | 121 | 1.79 | 0.0755 |
| **Group** | 0 | HFD | 5.00 | Non-est | . | . | . | . |
| **Group** | 0 | SD | 5.00 | Non-est | . | . | . | . |
| **Group** | HFD | SD | 5.00 | 3.8452 | 1.2988 | 162 | 2.96 | 0.0035 |
| **Group** | 0 | HFD | 6.00 | Non-est | . | . | . | . |
| **Group** | 0 | SD | 6.00 | Non-est | . | . | . | . |
| **Group** | HFD | SD | 6.00 | 5.9168 | 1.7135 | 194 | 3.45 | 0.0007 |

| **Model Information** | |
| --- | --- |
| **Data Set** | WORK.WEEKLY2 |
| **Dependent Variable** | wk_ketone |
| **Covariance Structure** | Autoregressive |
| **Subject Effect** | ID(Group) |
| **Estimation Method** | REML |
| **Residual Variance Method** | Profile |
| **Fixed Effects SE Method** | Kenward-Roger |
| **Degrees of Freedom Method** | Kenward-Roger |

| **Class Level Information** | | |
| --- | --- | --- |
| **Class** | **Levels** | **Values** |
| **Group** | 3 | 0 HFD SD |
| **ID** | 49 | 0 C1 C10 C11 C12 C13 C14 C15 C16 C17 C18 C19 C2 C20 C21 C22 C23 C24 C25 C26 C3 C4 C5 C6 C7 C8 C9 T1 T10 T11 T12 T13 T14 T15 T17 T2 T21 T22 T23 T24 T26 T27 T3 T4 T5 T6 T7 T8 T9 |

| **Dimensions** | |
| --- | --- |
| **Covariance Parameters** | 2 |
| **Columns in X** | 8 |
| **Columns in Z** | 0 |
| **Subjects** | 49 |
| **Max Obs per Subject** | 157 |

| **Number of Observations** | |
| --- | --- |
| **Number of Observations Read** | 445 |
| **Number of Observations Used** | 413 |
| **Number of Observations Not Used** | 32 |

| **Iteration History** | | | |
| --- | --- | --- | --- |
| **Iteration** | **Evaluations** | **-2 Res Log Like** | **Criterion** |
| **0** | 1 | 311.35190297 |  |
| **1** | 2 | 51.02149537 | 0.00005194 |
| **2** | 1 | 51.00325041 | 0.00000000 |

| Convergence criteria met. |
| --- |

| **Covariance Parameter Estimates** | | |
| --- | --- | --- |
| **Cov Parm** | **Subject** | **Estimate** |
| **AR(1)** | ID(Group) | 0.7407 |
| **Residual** |  | 0.1278 |

| **Fit Statistics** | |
| --- | --- |
| **-2 Res Log Likelihood** | 51.0 |
| **AIC (Smaller is Better)** | 55.0 |
| **AICC (Smaller is Better)** | 55.0 |
| **BIC (Smaller is Better)** | 58.8 |

| **Null Model Likelihood Ratio Test** | | |
| --- | --- | --- |
| **DF** | **Chi-Square** | **Pr > ChiSq** |
| 1 | 260.35 | <.0001 |

| **Solution for Fixed Effects** | | | | | | |
| --- | --- | --- | --- | --- | --- | --- |
| **Effect** | **Group** | **Estimate** | **Standard Error** | **DF** | **t Value** | **Pr > |t|** |
| **Intercept** |  | 1.0507 | 0.08273 | 171 | 12.70 | <.0001 |
| **Group** | 0 | -1.0507 | 0.1093 | 72.8 | -9.61 | <.0001 |
| **Group** | HFD | -0.3221 | 0.1217 | 168 | -2.65 | 0.0089 |
| **Group** | SD | 0 | . | . | . | . |
| **Week** |  | -0.03795 | 0.01987 | 360 | -1.91 | 0.0569 |
| **Week*Group** | 0 | 0 | . | . | . | . |
| **Week*Group** | HFD | -0.00505 | 0.02872 | 353 | -0.18 | 0.8604 |
| **Week*Group** | SD | 0 | . | . | . | . |

| **Type 3 Tests of Fixed Effects** | | | | |
| --- | --- | --- | --- | --- |
| **Effect** | **Num DF** | **Den DF** | **F Value** | **Pr > F** |
| **Group** | 2 | 88.4 | 49.70 | <.0001 |
| **Week** | 1 | 353 | 7.95 | 0.0051 |
| **Week*Group** | 1 | 353 | 0.03 | 0.8604 |

| **Least Squares Means** | | | | | | | |
| --- | --- | --- | --- | --- | --- | --- | --- |
| **Effect** | **Group** | **Week** | **Estimate** | **Standard Error** | **DF** | **t Value** | **Pr > |t|** |
| **Group** | 0 | 1.00 | Non-est | . | . | . | . |
| **Group** | HFD | 1.00 | 0.6856 | 0.07496 | 121 | 9.15 | <.0001 |
| **Group** | SD | 1.00 | 1.0127 | 0.06909 | 123 | 14.66 | <.0001 |
| **Group** | 0 | 2.00 | Non-est | . | . | . | . |
| **Group** | HFD | 2.00 | 0.6426 | 0.06422 | 85.7 | 10.01 | <.0001 |
| **Group** | SD | 2.00 | 0.9748 | 0.05909 | 85.6 | 16.50 | <.0001 |
| **Group** | 0 | 3.00 | Non-est | . | . | . | . |
| **Group** | HFD | 3.00 | 0.5996 | 0.05907 | 69.2 | 10.15 | <.0001 |
| **Group** | SD | 3.00 | 0.9368 | 0.05476 | 70 | 17.11 | <.0001 |
| **Group** | 0 | 4.00 | Non-est | . | . | . | . |
| **Group** | HFD | 4.00 | 0.5566 | 0.06095 | 74.8 | 9.13 | <.0001 |
| **Group** | SD | 4.00 | 0.8989 | 0.05741 | 79.2 | 15.66 | <.0001 |
| **Group** | 0 | 5.00 | Non-est | . | . | . | . |
| **Group** | HFD | 5.00 | 0.5136 | 0.06928 | 102 | 7.41 | <.0001 |
| **Group** | SD | 5.00 | 0.8609 | 0.06621 | 111 | 13.00 | <.0001 |
| **Group** | 0 | 6.00 | Non-est | . | . | . | . |
| **Group** | HFD | 6.00 | 0.4705 | 0.08212 | 143 | 5.73 | <.0001 |
| **Group** | SD | 6.00 | 0.8230 | 0.07912 | 158 | 10.40 | <.0001 |

| **Differences of Least Squares Means** | | | | | | | | |
| --- | --- | --- | --- | --- | --- | --- | --- | --- |
| **Effect** | **Group** | **_Group** | **Week** | **Estimate** | **Standard Error** | **DF** | **t Value** | **Pr > |t|** |
| **Group** | 0 | HFD | 1.00 | Non-est | . | . | . | . |
| **Group** | 0 | SD | 1.00 | Non-est | . | . | . | . |
| **Group** | HFD | SD | 1.00 | -0.3272 | 0.1019 | 122 | -3.21 | 0.0017 |
| **Group** | 0 | HFD | 2.00 | Non-est | . | . | . | . |
| **Group** | 0 | SD | 2.00 | Non-est | . | . | . | . |
| **Group** | HFD | SD | 2.00 | -0.3322 | 0.08727 | 85.7 | -3.81 | 0.0003 |
| **Group** | 0 | HFD | 3.00 | Non-est | . | . | . | . |
| **Group** | 0 | SD | 3.00 | Non-est | . | . | . | . |
| **Group** | HFD | SD | 3.00 | -0.3373 | 0.08055 | 69.6 | -4.19 | <.0001 |
| **Group** | 0 | HFD | 4.00 | Non-est | . | . | . | . |
| **Group** | 0 | SD | 4.00 | Non-est | . | . | . | . |
| **Group** | HFD | SD | 4.00 | -0.3423 | 0.08373 | 76.8 | -4.09 | 0.0001 |
| **Group** | 0 | HFD | 5.00 | Non-est | . | . | . | . |
| **Group** | 0 | SD | 5.00 | Non-est | . | . | . | . |
| **Group** | HFD | SD | 5.00 | -0.3474 | 0.09583 | 106 | -3.63 | 0.0004 |
| **Group** | 0 | HFD | 6.00 | Non-est | . | . | . | . |
| **Group** | 0 | SD | 6.00 | Non-est | . | . | . | . |
| **Group** | HFD | SD | 6.00 | -0.3524 | 0.1140 | 150 | -3.09 | 0.0024 |

**Week=5 Group=HFD**

| **Variable** | **N** | **Mean** | **Std Dev** | **Minimum** | **Maximum** |
| --- | --- | --- | --- | --- | --- |
| wk_timeX wk_timeA ratioxa | 12 12 12 | 41.7888889 31.4138889 1.4021417 | 16.3169388 11.1656260 0.4737145 | 26.4000000 17.3666667 0.7904825 | 80.8333333 50.4333333 2.2821497 |

**Week=5 Group=SD**

| **Variable** | **N** | **Mean** | **Std Dev** | **Minimum** | **Maximum** |
| --- | --- | --- | --- | --- | --- |
| wk_timeX wk_timeA ratioxa | 20 20 20 | 58.2183333 30.1716667 1.9897525 | 20.1996655 6.4255182 0.7833476 | 29.4666667 20.8000000 1.1198044 | 98.3000000 47.0000000 4.1122913 |

**Week=6 Group=HFD**

| **Variable** | **N** | **Mean** | **Std Dev** | **Minimum** | **Maximum** |
| --- | --- | --- | --- | --- | --- |
| wk_timeX wk_timeA ratioxa | 12 12 12 | 40.9500000 42.0333333 1.2405434 | 10.3140240 22.0290855 0.6787224 | 26.0000000 17.2000000 0.4311774 | 64.2000000 88.7000000 2.3627451 |

**Week=6 Group=SD**

| **Variable** | **N** | **Mean** | **Std Dev** | **Minimum** | **Maximum** |
| --- | --- | --- | --- | --- | --- |
| wk_timeX wk_timeA ratioxa | 20 20 20 | 51.5550000 35.7350000 1.6873956 | 15.6109771 14.1757493 0.8830547 | 27.8000000 17.7000000 0.5092593 | 78.5000000 75.6000000 3.8480392 |

| **11 Variables:** | noresponse Week wk_wt wk_glucose wk_ketone wk_distance wk_timeA wk_timeX wk_calintake ratioxa w2 |
| --- | --- |

| **Simple Statistics** | | | | | | |
| --- | --- | --- | --- | --- | --- | --- |
| **Variable** | **N** | **Mean** | **Std Dev** | **Sum** | **Minimum** | **Maximum** |
| **noresponse** | 12 | 0 | 0 | 0 | 0 | 0 |
| **Week** | 12 | 5.00000 | 0 | 60.00000 | 5.00000 | 5.00000 |
| **wk_wt** | 10 | 386.40000 | 16.11900 | 3864 | 365.00000 | 417.00000 |
| **wk_glucose** | 12 | 100.29167 | 6.31542 | 1204 | 88.00000 | 107.50000 |
| **wk_ketone** | 12 | 0.28333 | 0.36328 | 3.40000 | 0.10000 | 1.10000 |
| **wk_distance** | 10 | 3.72694 | 0.69973 | 37.26935 | 2.60465 | 4.77855 |
| **wk_timeA** | 12 | 31.41389 | 11.16563 | 376.96667 | 17.36667 | 50.43333 |
| **wk_timeX** | 12 | 41.78889 | 16.31694 | 501.46667 | 26.40000 | 80.83333 |
| **wk_calintake** | 10 | 57.31000 | 47.75044 | 573.10000 | 0 | 125.04000 |
| **ratioxa** | 12 | 1.40214 | 0.47371 | 16.82570 | 0.79048 | 2.28215 |
| **w2** | 12 | 25.00000 | 0 | 300.00000 | 25.00000 | 25.00000 |

| **Pearson Correlation Coefficients Prob > |r| under H0: Rho=0 Number of Observations** | | | | | | | |
| --- | --- | --- | --- | --- | --- | --- | --- |
|  | **noresponse** | **Week** | **wk_wt** | **wk_glucose** | **wk_ketone** | **wk_distance** | **wk_timeA** |
| **noresponse** | . . 12 | . . 12 | . . 10 | . . 12 | . . 12 | . . 10 | . . 12 |
| **Week** | . . 12 | . . 12 | . . 10 | . . 12 | . . 12 | . . 10 | . . 12 |
| **wk_wt** | . . 10 | . . 10 | 1.00000  10 | 0.14559 0.6882 10 | 0.49373 0.1470 10 | 0.25000 0.4860 10 | 0.12679 0.7271 10 |
| **wk_glucose** | . . 12 | . . 12 | 0.14559 0.6882 10 | 1.00000  12 | 0.21133 0.5097 12 | -0.37395 0.2871 10 | 0.77855 0.0029 12 |
| **wk_ketone** | . . 12 | . . 12 | 0.49373 0.1470 10 | 0.21133 0.5097 12 | 1.00000  12 | 0.34035 0.3359 10 | 0.35817 0.2530 12 |
| **wk_distance** | . . 10 | . . 10 | 0.25000 0.4860 10 | -0.37395 0.2871 10 | 0.34035 0.3359 10 | 1.00000  10 | -0.55593 0.0952 10 |
| **wk_timeA** | . . 12 | . . 12 | 0.12679 0.7271 10 | 0.77855 0.0029 12 | 0.35817 0.2530 12 | -0.55593 0.0952 10 | 1.00000  12 |
| **wk_timeX** | . . 12 | . . 12 | 0.39303 0.2612 10 | 0.24405 0.4446 12 | 0.89384 <.0001 12 | -0.25661 0.4742 10 | 0.49883 0.0988 12 |
| **wk_calintake** | . . 10 | . . 10 | 0.46631 0.1743 10 | -0.10281 0.7775 10 | 0.25877 0.4703 10 | 0.84491 0.0021 10 | -0.36032 0.3064 10 |
| **ratioxa** | . . 12 | . . 12 | -0.10871 0.7650 10 | -0.51469 0.0869 12 | 0.43505 0.1575 12 | 0.27434 0.4430 10 | -0.46567 0.1271 12 |
| **w2** | . . 12 | . . 12 | . . 10 | . . 12 | . . 12 | . . 10 | . . 12 |

| **Pearson Correlation Coefficients Prob > |r| under H0: Rho=0 Number of Observations** | | | | |
| --- | --- | --- | --- | --- |
|  | **wk_timeX** | **wk_calintake** | **ratioxa** | **w2** |
| **noresponse** | . . 12 | . . 10 | . . 12 | . . 12 |
| **Week** | . . 12 | . . 10 | . . 12 | . . 12 |
| **wk_wt** | 0.39303 0.2612 10 | 0.46631 0.1743 10 | -0.10871 0.7650 10 | . . 10 |
| **wk_glucose** | 0.24405 0.4446 12 | -0.10281 0.7775 10 | -0.51469 0.0869 12 | . . 12 |
| **wk_ketone** | 0.89384 <.0001 12 | 0.25877 0.4703 10 | 0.43505 0.1575 12 | . . 12 |
| **wk_distance** | -0.25661 0.4742 10 | 0.84491 0.0021 10 | 0.27434 0.4430 10 | . . 10 |
| **wk_timeA** | 0.49883 0.0988 12 | -0.36032 0.3064 10 | -0.46567 0.1271 12 | . . 12 |
| **wk_timeX** | 1.00000  12 | -0.31135 0.3812 10 | 0.48056 0.1138 12 | . . 12 |
| **wk_calintake** | -0.31135 0.3812 10 | 1.00000  10 | -0.03477 0.9240 10 | . . 10 |
| **ratioxa** | 0.48056 0.1138 12 | -0.03477 0.9240 10 | 1.00000  12 | . . 12 |
| **w2** | . . 12 | . . 10 | . . 12 | . . 12 |

| **11 Variables:** | noresponse Week wk_wt wk_glucose wk_ketone wk_distance wk_timeA wk_timeX wk_calintake ratioxa w2 |
| --- | --- |

**Group=HFD Week=5**

| **Variable** | **N** | **Mean** | **Std Dev** | **Minimum** | **Maximum** |
| --- | --- | --- | --- | --- | --- |
| wk_timeX wk_timeA ratioxa | 12 12 12 | 41.7888889 31.4138889 1.4021417 | 16.3169388 11.1656260 0.4737145 | 26.4000000 17.3666667 0.7904825 | 80.8333333 50.4333333 2.2821497 |

**Group=HFD Week=6**

| **Variable** | **N** | **Mean** | **Std Dev** | **Minimum** | **Maximum** |
| --- | --- | --- | --- | --- | --- |
| wk_timeX wk_timeA ratioxa | 12 12 12 | 40.9500000 42.0333333 1.2405434 | 10.3140240 22.0290855 0.6787224 | 26.0000000 17.2000000 0.4311774 | 64.2000000 88.7000000 2.3627451 |

**Group=SD Week=5**

| **Variable** | **N** | **Mean** | **Std Dev** | **Minimum** | **Maximum** |
| --- | --- | --- | --- | --- | --- |
| wk_timeX wk_timeA ratioxa | 20 20 20 | 58.2183333 30.1716667 1.9897525 | 20.1996655 6.4255182 0.7833476 | 29.4666667 20.8000000 1.1198044 | 98.3000000 47.0000000 4.1122913 |

**Group=SD Week=6**

| **Variable** | **N** | **Mean** | **Std Dev** | **Minimum** | **Maximum** |
| --- | --- | --- | --- | --- | --- |
| wk_timeX wk_timeA ratioxa | 20 20 20 | 51.5550000 35.7350000 1.6873956 | 15.6109771 14.1757493 0.8830547 | 27.8000000 17.7000000 0.5092593 | 78.5000000 75.6000000 3.8480392 |

| **Model Information** | |
| --- | --- |
| **Data Set** | WORK.SHORT |
| **Dependent Variable** | wk_timeX |
| **Covariance Structure** | Diagonal |
| **Estimation Method** | REML |
| **Residual Variance Method** | Profile |
| **Fixed Effects SE Method** | Model-Based |
| **Degrees of Freedom Method** | Residual |

| **Class Level Information** | | |
| --- | --- | --- |
| **Class** | **Levels** | **Values** |
| **Group** | 2 | HFD SD |
| **ID** | 32 | C10 C11 C12 C13 C14 C15 C16 C17 C18 C19 C20 C21 C22 C23 C24 C25 C26 C7 C8 C9 T11 T12 T13 T14 T15 T17 T21 T22 T23 T24 T26 T27 |

| **Dimensions** | |
| --- | --- |
| **Covariance Parameters** | 1 |
| **Columns in X** | 3 |
| **Columns in Z** | 0 |
| **Subjects** | 1 |
| **Max Obs per Subject** | 32 |

| **Number of Observations** | |
| --- | --- |
| **Number of Observations Read** | 32 |
| **Number of Observations Used** | 32 |
| **Number of Observations Not Used** | 0 |

| **Covariance Parameter Estimates** | |
| --- | --- |
| **Cov Parm** | **Estimate** |
| **Residual** | 356.04 |

| **Fit Statistics** | |
| --- | --- |
| **-2 Res Log Likelihood** | 266.9 |
| **AIC (Smaller is Better)** | 268.9 |
| **AICC (Smaller is Better)** | 269.0 |
| **BIC (Smaller is Better)** | 270.3 |

| **Type 3 Tests of Fixed Effects** | | | | |
| --- | --- | --- | --- | --- |
| **Effect** | **Num DF** | **Den DF** | **F Value** | **Pr > F** |
| **Group** | 1 | 30 | 5.69 | 0.0236 |

| **Least Squares Means** | | | | | | |
| --- | --- | --- | --- | --- | --- | --- |
| **Effect** | **Group** | **Estimate** | **Standard Error** | **DF** | **t Value** | **Pr > |t|** |
| **Group** | HFD | 41.7889 | 5.4470 | 30 | 7.67 | <.0001 |
| **Group** | SD | 58.2183 | 4.2192 | 30 | 13.80 | <.0001 |

| **Differences of Least Squares Means** | | | | | | | |
| --- | --- | --- | --- | --- | --- | --- | --- |
| **Effect** | **Group** | **_Group** | **Estimate** | **Standard Error** | **DF** | **t Value** | **Pr > |t|** |
| **Group** | HFD | SD | -16.4294 | 6.8900 | 30 | -2.38 | 0.0236 |

| **Model Information** | |
| --- | --- |
| **Data Set** | WORK.SHORT |
| **Dependent Variable** | wk_timeX |
| **Covariance Structure** | Diagonal |
| **Estimation Method** | REML |
| **Residual Variance Method** | Profile |
| **Fixed Effects SE Method** | Model-Based |
| **Degrees of Freedom Method** | Residual |

| **Class Level Information** | | |
| --- | --- | --- |
| **Class** | **Levels** | **Values** |
| **Group** | 2 | HFD SD |
| **ID** | 32 | C10 C11 C12 C13 C14 C15 C16 C17 C18 C19 C20 C21 C22 C23 C24 C25 C26 C7 C8 C9 T11 T12 T13 T14 T15 T17 T21 T22 T23 T24 T26 T27 |

| **Dimensions** | |
| --- | --- |
| **Covariance Parameters** | 1 |
| **Columns in X** | 3 |
| **Columns in Z** | 0 |
| **Subjects** | 1 |
| **Max Obs per Subject** | 32 |

| **Number of Observations** | |
| --- | --- |
| **Number of Observations Read** | 32 |
| **Number of Observations Used** | 32 |
| **Number of Observations Not Used** | 0 |

| **Covariance Parameter Estimates** | |
| --- | --- |
| **Cov Parm** | **Estimate** |
| **Residual** | 193.35 |

| **Fit Statistics** | |
| --- | --- |
| **-2 Res Log Likelihood** | 248.6 |
| **AIC (Smaller is Better)** | 250.6 |
| **AICC (Smaller is Better)** | 250.7 |
| **BIC (Smaller is Better)** | 252.0 |

| **Type 3 Tests of Fixed Effects** | | | | |
| --- | --- | --- | --- | --- |
| **Effect** | **Num DF** | **Den DF** | **F Value** | **Pr > F** |
| **Group** | 1 | 30 | 4.36 | 0.0453 |

| **Least Squares Means** | | | | | | |
| --- | --- | --- | --- | --- | --- | --- |
| **Effect** | **Group** | **Estimate** | **Standard Error** | **DF** | **t Value** | **Pr > |t|** |
| **Group** | HFD | 40.9500 | 4.0140 | 30 | 10.20 | <.0001 |
| **Group** | SD | 51.5550 | 3.1093 | 30 | 16.58 | <.0001 |

| **Differences of Least Squares Means** | | | | | | | |
| --- | --- | --- | --- | --- | --- | --- | --- |
| **Effect** | **Group** | **_Group** | **Estimate** | **Standard Error** | **DF** | **t Value** | **Pr > |t|** |
| **Group** | HFD | SD | -10.6050 | 5.0774 | 30 | -2.09 | 0.0453 |

| **Model Information** | |
| --- | --- |
| **Data Set** | WORK.SHORT |
| **Dependent Variable** | transratio |
| **Covariance Structure** | Diagonal |
| **Estimation Method** | REML |
| **Residual Variance Method** | Profile |
| **Fixed Effects SE Method** | Model-Based |
| **Degrees of Freedom Method** | Residual |

| **Class Level Information** | | |
| --- | --- | --- |
| **Class** | **Levels** | **Values** |
| **Group** | 2 | HFD SD |
| **ID** | 32 | C10 C11 C12 C13 C14 C15 C16 C17 C18 C19 C20 C21 C22 C23 C24 C25 C26 C7 C8 C9 T11 T12 T13 T14 T15 T17 T21 T22 T23 T24 T26 T27 |

| **Dimensions** | |
| --- | --- |
| **Covariance Parameters** | 1 |
| **Columns in X** | 3 |
| **Columns in Z** | 0 |
| **Subjects** | 1 |
| **Max Obs per Subject** | 32 |

| **Number of Observations** | |
| --- | --- |
| **Number of Observations Read** | 32 |
| **Number of Observations Used** | 32 |
| **Number of Observations Not Used** | 0 |

| **Covariance Parameter Estimates** | |
| --- | --- |
| **Cov Parm** | **Estimate** |
| **Residual** | 0.1282 |

| **Fit Statistics** | |
| --- | --- |
| **-2 Res Log Likelihood** | 29.0 |
| **AIC (Smaller is Better)** | 31.0 |
| **AICC (Smaller is Better)** | 31.1 |
| **BIC (Smaller is Better)** | 32.4 |

| **Type 3 Tests of Fixed Effects** | | | | |
| --- | --- | --- | --- | --- |
| **Effect** | **Num DF** | **Den DF** | **F Value** | **Pr > F** |
| **Group** | 1 | 30 | 6.50 | 0.0162 |

| **Least Squares Means** | | | | | | |
| --- | --- | --- | --- | --- | --- | --- |
| **Effect** | **Group** | **Estimate** | **Standard Error** | **DF** | **t Value** | **Pr > |t|** |
| **Group** | HFD | 0.2872 | 0.1033 | 30 | 2.78 | 0.0093 |
| **Group** | SD | 0.6204 | 0.08005 | 30 | 7.75 | <.0001 |

| **Differences of Least Squares Means** | | | | | | | |
| --- | --- | --- | --- | --- | --- | --- | --- |
| **Effect** | **Group** | **_Group** | **Estimate** | **Standard Error** | **DF** | **t Value** | **Pr > |t|** |
| **Group** | HFD | SD | -0.3332 | 0.1307 | 30 | -2.55 | 0.0162 |

| **Model Information** | |
| --- | --- |
| **Data Set** | WORK.SHORT |
| **Dependent Variable** | transratio |
| **Covariance Structure** | Diagonal |
| **Estimation Method** | REML |
| **Residual Variance Method** | Profile |
| **Fixed Effects SE Method** | Model-Based |
| **Degrees of Freedom Method** | Residual |

| **Class Level Information** | | |
| --- | --- | --- |
| **Class** | **Levels** | **Values** |
| **Group** | 2 | HFD SD |
| **ID** | 32 | C10 C11 C12 C13 C14 C15 C16 C17 C18 C19 C20 C21 C22 C23 C24 C25 C26 C7 C8 C9 T11 T12 T13 T14 T15 T17 T21 T22 T23 T24 T26 T27 |

| **Dimensions** | |
| --- | --- |
| **Covariance Parameters** | 1 |
| **Columns in X** | 3 |
| **Columns in Z** | 0 |
| **Subjects** | 1 |
| **Max Obs per Subject** | 32 |

| **Number of Observations** | |
| --- | --- |
| **Number of Observations Read** | 32 |
| **Number of Observations Used** | 32 |
| **Number of Observations Not Used** | 0 |

| **Covariance Parameter Estimates** | |
| --- | --- |
| **Cov Parm** | **Estimate** |
| **Residual** | 0.3172 |

| **Fit Statistics** | |
| --- | --- |
| **-2 Res Log Likelihood** | 56.2 |
| **AIC (Smaller is Better)** | 58.2 |
| **AICC (Smaller is Better)** | 58.3 |
| **BIC (Smaller is Better)** | 59.6 |

| **Type 3 Tests of Fixed Effects** | | | | |
| --- | --- | --- | --- | --- |
| **Effect** | **Num DF** | **Den DF** | **F Value** | **Pr > F** |
| **Group** | 1 | 30 | 2.52 | 0.1231 |

| **Least Squares Means** | | | | | | |
| --- | --- | --- | --- | --- | --- | --- |
| **Effect** | **Group** | **Estimate** | **Standard Error** | **DF** | **t Value** | **Pr > |t|** |
| **Group** | HFD | 0.06387 | 0.1626 | 30 | 0.39 | 0.6972 |
| **Group** | SD | 0.3901 | 0.1259 | 30 | 3.10 | 0.0042 |

| **Differences of Least Squares Means** | | | | | | | |
| --- | --- | --- | --- | --- | --- | --- | --- |
| **Effect** | **Group** | **_Group** | **Estimate** | **Standard Error** | **DF** | **t Value** | **Pr > |t|** |
| **Group** | HFD | SD | -0.3263 | 0.2057 | 30 | -1.59 | 0.1231 |

| **Moments** | | | |
| --- | --- | --- | --- |
| **N** | 64 | **Sum Weights** | 64 |
| **Mean** | 0 | **Sum Observations** | 0 |
| **Std Deviation** | 0.46053856 | **Variance** | 0.21209577 |
| **Skewness** | -0.1491146 | **Kurtosis** | -0.2733324 |
| **Uncorrected SS** | 13.3620334 | **Corrected SS** | 13.3620334 |
| **Coeff Variation** | . | **Std Error Mean** | 0.05756732 |

| **Basic Statistical Measures** | | | |
| --- | --- | --- | --- |
| **Location** | | **Variability** | |
| **Mean** | 0.00000 | **Std Deviation** | 0.46054 |
| **Median** | -0.00987 | **Variance** | 0.21210 |
| **Mode** | . | **Range** | 2.02236 |
|  |  | **Interquartile Range** | 0.64889 |

| **Tests for Location: Mu0=0** | | | | |
| --- | --- | --- | --- | --- |
| **Test** | **Statistic** | | **p Value** | |
| **Student's t** | **t** | 0 | **Pr > |t|** | 1.0000 |
| **Sign** | **M** | 0 | **Pr >= |M|** | 1.0000 |
| **Signed Rank** | **S** | 24 | **Pr >= |S|** | 0.8740 |

| **Tests for Normality** | | | | |
| --- | --- | --- | --- | --- |
| **Test** | **Statistic** | | **p Value** | |
| **Shapiro-Wilk** | **W** | 0.987721 | **Pr < W** | 0.7771 |
| **Kolmogorov-Smirnov** | **D** | 0.06842 | **Pr > D** | >0.1500 |
| **Cramer-von Mises** | **W-Sq** | 0.024546 | **Pr > W-Sq** | >0.2500 |
| **Anderson-Darling** | **A-Sq** | 0.186567 | **Pr > A-Sq** | >0.2500 |

| **Quantiles (Definition 5)** | |
| --- | --- |
| **Level** | **Quantile** |
| **100% Max** | 0.95743606 |
| **99%** | 0.95743606 |
| **95%** | 0.79357096 |
| **90%** | 0.61381640 |
| **75% Q3** | 0.29901717 |
| **50% Median** | -0.00987042 |
| **25% Q1** | -0.34986803 |
| **10%** | -0.52229840 |
| **5%** | -0.83556235 |
| **1%** | -1.06492571 |
| **0% Min** | -1.06492571 |

| **Extreme Observations** | | | |
| --- | --- | --- | --- |
| **Lowest** | | **Highest** | |
| **Value** | **Obs** | **Value** | **Obs** |
| -1.064926 | 47 | 0.661224 | 44 |
| -1.034922 | 46 | 0.793571 | 27 |
| -0.905110 | 34 | 0.795950 | 42 |
| -0.835562 | 37 | 0.823795 | 63 |
| -0.639865 | 50 | 0.957436 | 52 |

| **Model Information** | |
| --- | --- |
| **Data Set** | WORK.DISTANCE |
| **Dependent Variable** | distance |
| **Covariance Structure** | Autoregressive |
| **Subject Effect** | ID(Group) |
| **Estimation Method** | REML |
| **Residual Variance Method** | Profile |
| **Fixed Effects SE Method** | Kenward-Roger |
| **Degrees of Freedom Method** | Kenward-Roger |

| **Class Level Information** | | |
| --- | --- | --- |
| **Class** | **Levels** | **Values** |
| **Group** | 2 | HFD SD |
| **ID** | 48 | C1 C10 C11 C12 C13 C14 C15 C16 C17 C18 C19 C2 C20 C21 C22 C23 C24 C25 C26 C3 C4 C5 C6 C7 C8 C9 T1 T10 T11 T12 T13 T14 T15 T17 T2 T21 T22 T23 T24 T26 T27 T3 T4 T5 T6 T7 T8 T9 |

| **Dimensions** | |
| --- | --- |
| **Covariance Parameters** | 2 |
| **Columns in X** | 6 |
| **Columns in Z** | 0 |
| **Subjects** | 48 |
| **Max Obs per Subject** | 8 |

| **Number of Observations** | |
| --- | --- |
| **Number of Observations Read** | 383 |
| **Number of Observations Used** | 383 |
| **Number of Observations Not Used** | 0 |

| **Iteration History** | | | |
| --- | --- | --- | --- |
| **Iteration** | **Evaluations** | **-2 Res Log Like** | **Criterion** |
| **0** | 1 | 994.76932336 |  |
| **1** | 2 | 824.72980878 | 0.00004554 |
| **2** | 1 | 824.72688718 | 0.00000000 |

| Convergence criteria met. |
| --- |

| **Covariance Parameter Estimates** | | |
| --- | --- | --- |
| **Cov Parm** | **Subject** | **Estimate** |
| **AR(1)** | ID(Group) | 0.6111 |
| **Residual** |  | 0.7375 |

| **Fit Statistics** | |
| --- | --- |
| **-2 Res Log Likelihood** | 824.7 |
| **AIC (Smaller is Better)** | 828.7 |
| **AICC (Smaller is Better)** | 828.8 |
| **BIC (Smaller is Better)** | 832.5 |

| **Null Model Likelihood Ratio Test** | | |
| --- | --- | --- |
| **DF** | **Chi-Square** | **Pr > ChiSq** |
| 1 | 170.04 | <.0001 |

| **Solution for Fixed Effects** | | | | | | |
| --- | --- | --- | --- | --- | --- | --- |
| **Effect** | **Group** | **Estimate** | **Standard Error** | **DF** | **t Value** | **Pr > |t|** |
| **Intercept** |  | 3.9133 | 0.1521 | 130 | 25.72 | <.0001 |
| **Group** | HFD | -0.4733 | 0.2263 | 130 | -2.09 | 0.0384 |
| **Group** | SD | 0 | . | . | . | . |
| **distDay** |  | 0.008007 | 0.03290 | 214 | 0.24 | 0.8079 |
| **distDay*Group** | HFD | 0.06748 | 0.04863 | 212 | 1.39 | 0.1667 |
| **distDay*Group** | SD | 0 | . | . | . | . |

| **Type 3 Tests of Fixed Effects** | | | | |
| --- | --- | --- | --- | --- |
| **Effect** | **Num DF** | **Den DF** | **F Value** | **Pr > F** |
| **Group** | 1 | 130 | 4.37 | 0.0384 |
| **distDay** | 1 | 212 | 2.95 | 0.0875 |
| **distDay*Group** | 1 | 212 | 1.93 | 0.1667 |

| **Least Squares Means** | | | | | | | |
| --- | --- | --- | --- | --- | --- | --- | --- |
| **Effect** | **Group** | **distDay** | **Estimate** | **Standard Error** | **DF** | **t Value** | **Pr > |t|** |
| **Group** | HFD | 3.46 | 3.7012 | 0.1112 | 76.1 | 33.29 | <.0001 |
| **Group** | SD | 3.46 | 3.9410 | 0.1024 | 76.3 | 38.47 | <.0001 |
| **Group** | HFD | 7.00 | 3.9684 | 0.1675 | 130 | 23.69 | <.0001 |
| **Group** | SD | 7.00 | 3.9694 | 0.1561 | 132 | 25.43 | <.0001 |

| **Differences of Least Squares Means** | | | | | | | | |
| --- | --- | --- | --- | --- | --- | --- | --- | --- |
| **Effect** | **Group** | **_Group** | **distDay** | **Estimate** | **Standard Error** | **DF** | **t Value** | **Pr > |t|** |
| **Group** | HFD | SD | 3.46 | -0.2398 | 0.1512 | 76.2 | -1.59 | 0.1168 |
| **Group** | HFD | SD | 7.00 | -0.00093 | 0.2290 | 131 | -0.00 | 0.9968 |

| **Moments** | | | |
| --- | --- | --- | --- |
| **N** | 383 | **Sum Weights** | 383 |
| **Mean** | -0.0316765 | **Sum Observations** | -12.132081 |
| **Std Deviation** | 0.86766368 | **Variance** | 0.75284026 |
| **Skewness** | -0.2236073 | **Kurtosis** | 0.74049691 |
| **Uncorrected SS** | 287.969279 | **Corrected SS** | 287.584978 |
| **Coeff Variation** | -2739.1441 | **Std Error Mean** | 0.04433554 |

| **Basic Statistical Measures** | | | |
| --- | --- | --- | --- |
| **Location** | | **Variability** | |
| **Mean** | -0.03168 | **Std Deviation** | 0.86766 |
| **Median** | -0.02212 | **Variance** | 0.75284 |
| **Mode** | . | **Range** | 5.58337 |
|  |  | **Interquartile Range** | 1.06603 |

| **Tests for Location: Mu0=0** | | | | |
| --- | --- | --- | --- | --- |
| **Test** | **Statistic** | | **p Value** | |
| **Student's t** | **t** | -0.71447 | **Pr > |t|** | 0.4754 |
| **Sign** | **M** | -3.5 | **Pr >= |M|** | 0.7592 |
| **Signed Rank** | **S** | -1042 | **Pr >= |S|** | 0.6314 |

| **Tests for Normality** | | | | |
| --- | --- | --- | --- | --- |
| **Test** | **Statistic** | | **p Value** | |
| **Shapiro-Wilk** | **W** | 0.991581 | **Pr < W** | 0.0285 |
| **Kolmogorov-Smirnov** | **D** | 0.042327 | **Pr > D** | 0.0929 |
| **Cramer-von Mises** | **W-Sq** | 0.08867 | **Pr > W-Sq** | 0.1630 |
| **Anderson-Darling** | **A-Sq** | 0.652827 | **Pr > A-Sq** | 0.0904 |

| **Quantiles (Definition 5)** | |
| --- | --- |
| **Level** | **Quantile** |
| **100% Max** | 2.4166527 |
| **99%** | 2.1377111 |
| **95%** | 1.3435785 |
| **90%** | 0.9995454 |
| **75% Q3** | 0.5132893 |
| **50% Median** | -0.0221215 |
| **25% Q1** | -0.5527400 |
| **10%** | -1.1458253 |
| **5%** | -1.4173755 |
| **1%** | -2.2638180 |
| **0% Min** | -3.1667159 |

| **Extreme Observations** | | | |
| --- | --- | --- | --- |
| **Lowest** | | **Highest** | |
| **Value** | **Obs** | **Value** | **Obs** |
| -3.16672 | 165 | 2.08568 | 45 |
| -2.82083 | 302 | 2.13771 | 40 |
| -2.80364 | 334 | 2.29862 | 56 |
| -2.26382 | 239 | 2.31586 | 93 |
| -2.16665 | 364 | 2.41665 | 109 |
